# Supplementary figures and images for: Lactate transporter MCT1 in hepatic stellate cells promotes fibrotic collagen expression in nonalcoholic steatohepatitis
Source: eLife. 2024 Apr 2;12:RP89136. doi: 10.7554/eLife.89136 (PMC10987092; doi:10.7554/eLife.89136)

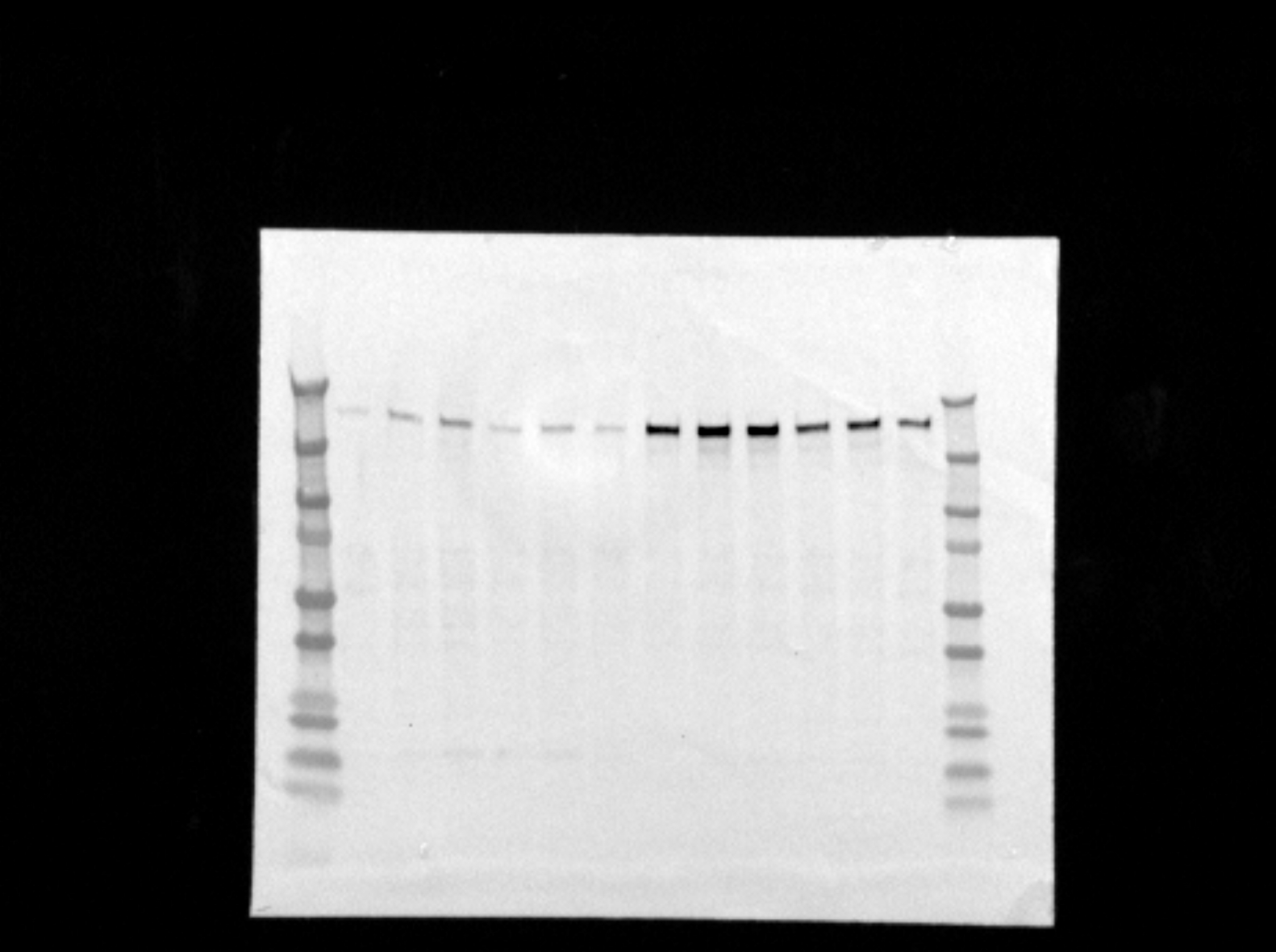

Supplement: Figure 1—source data 1. [file elife-89136-fig1-data1.zip › Figure 1-Source Data/Figure 1-Source Data-2 (raw WB images)/Figure 1B COL1.jpg]

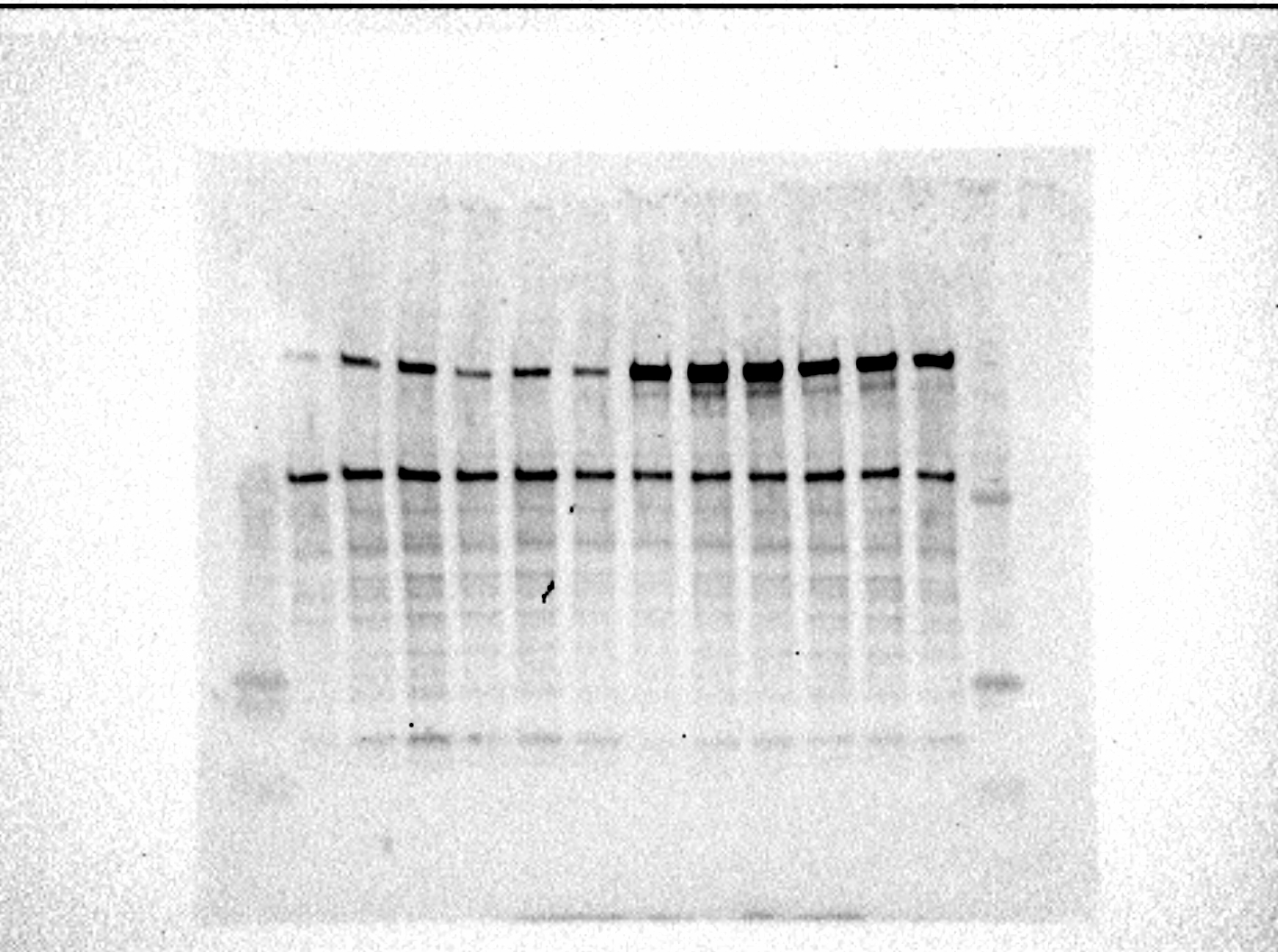

Supplement: Figure 1—source data 1. [file elife-89136-fig1-data1.zip › Figure 1-Source Data/Figure 1-Source Data-2 (raw WB images)/Figure 1B HSP90.jpg]

## Slide 1
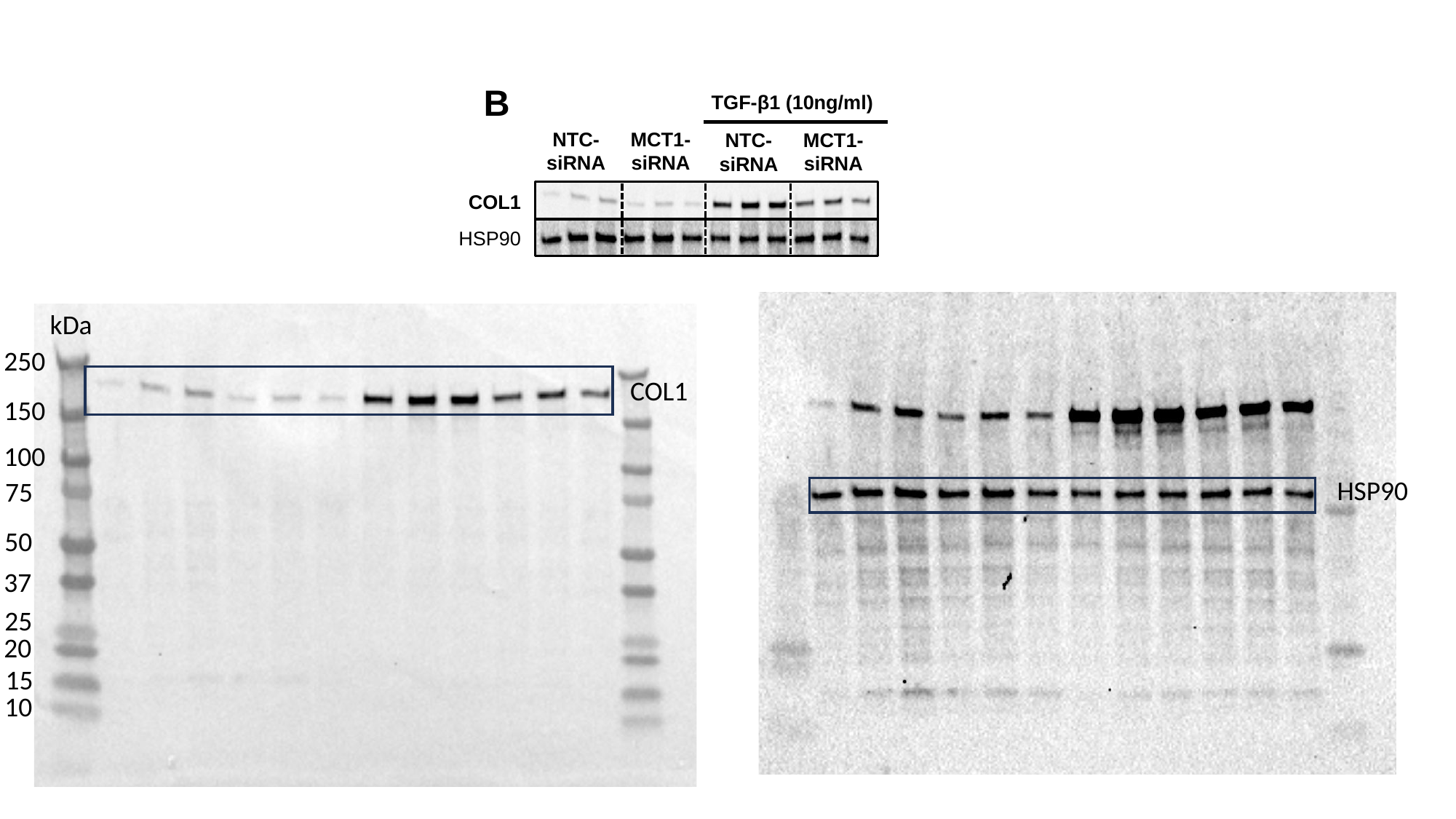

B
TGF-β1 (10ng/ml)
MCT1-
siRNA
NTC-
siRNA
COL1
HSP90
MCT1-
siRNA
NTC-
siRNA
kDa
250
COL1
150
100
HSP90
75
50
37
25
20
15
10

Supplement: Figure 1—source data 1. [file elife-89136-fig1-data1.zip › Figure 1-Source Data/Figure 1-Source Data-3 (labeled WB images).pptx]

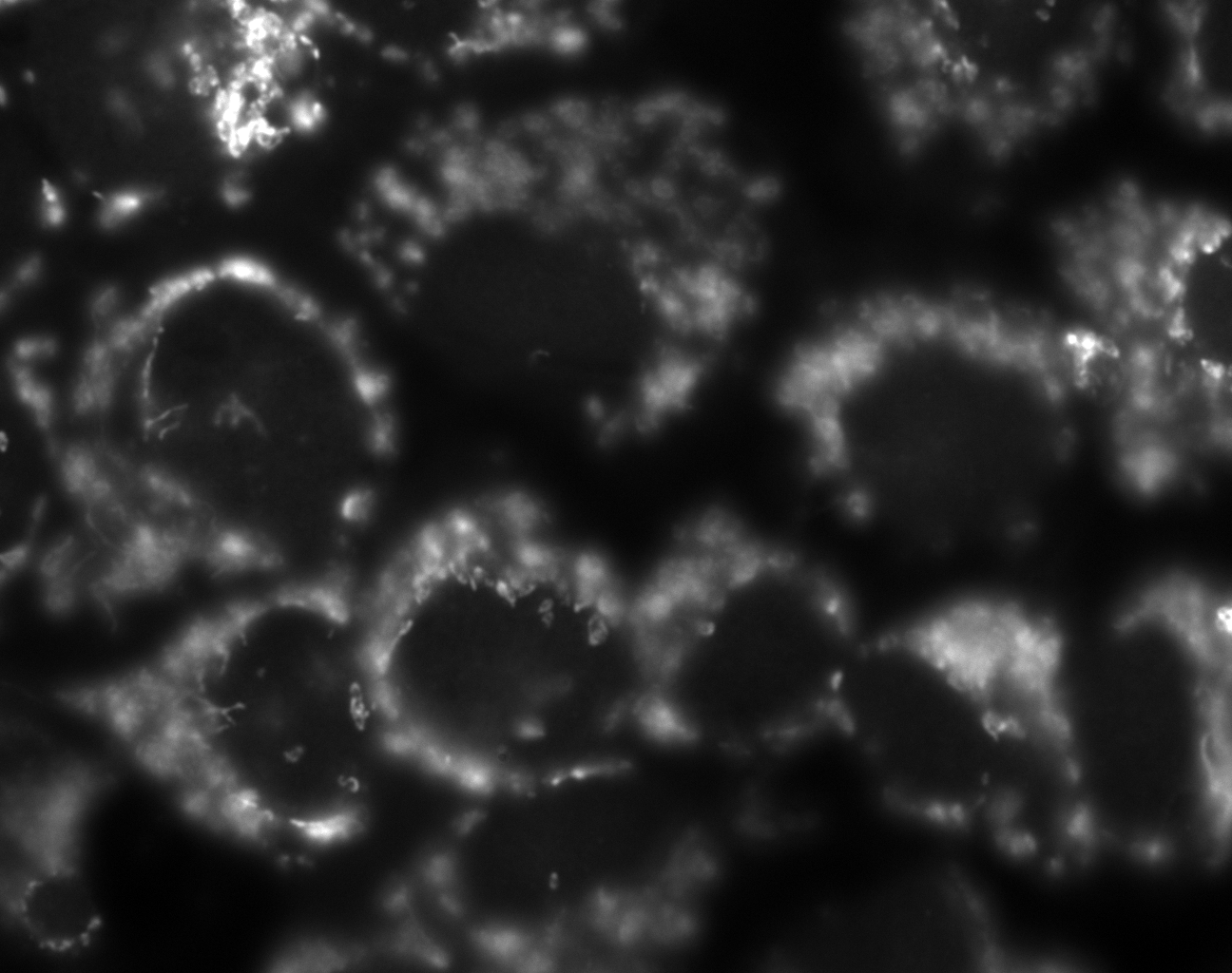

Supplement: Figure 2—source data 1. [file elife-89136-fig2-data1.zip › Figure 2-Source Data/Figure 2-Source Data-2 (raw IF images)/Fig2D-Chol-MCT1-2060-Cy3.TIF]

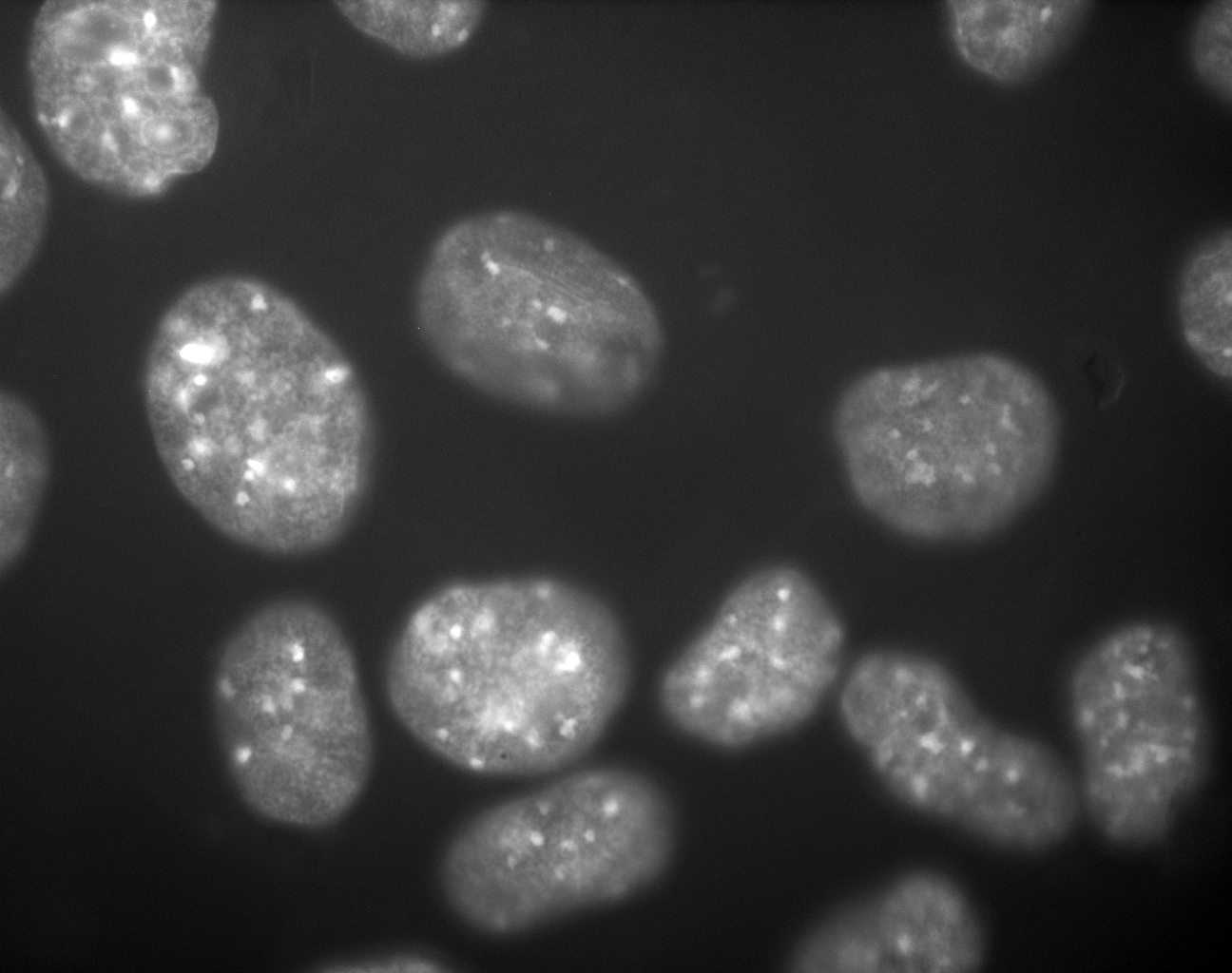

Supplement: Figure 2—source data 1. [file elife-89136-fig2-data1.zip › Figure 2-Source Data/Figure 2-Source Data-2 (raw IF images)/Fig2D-Chol-MCT1-2060-DAPI.TIF]

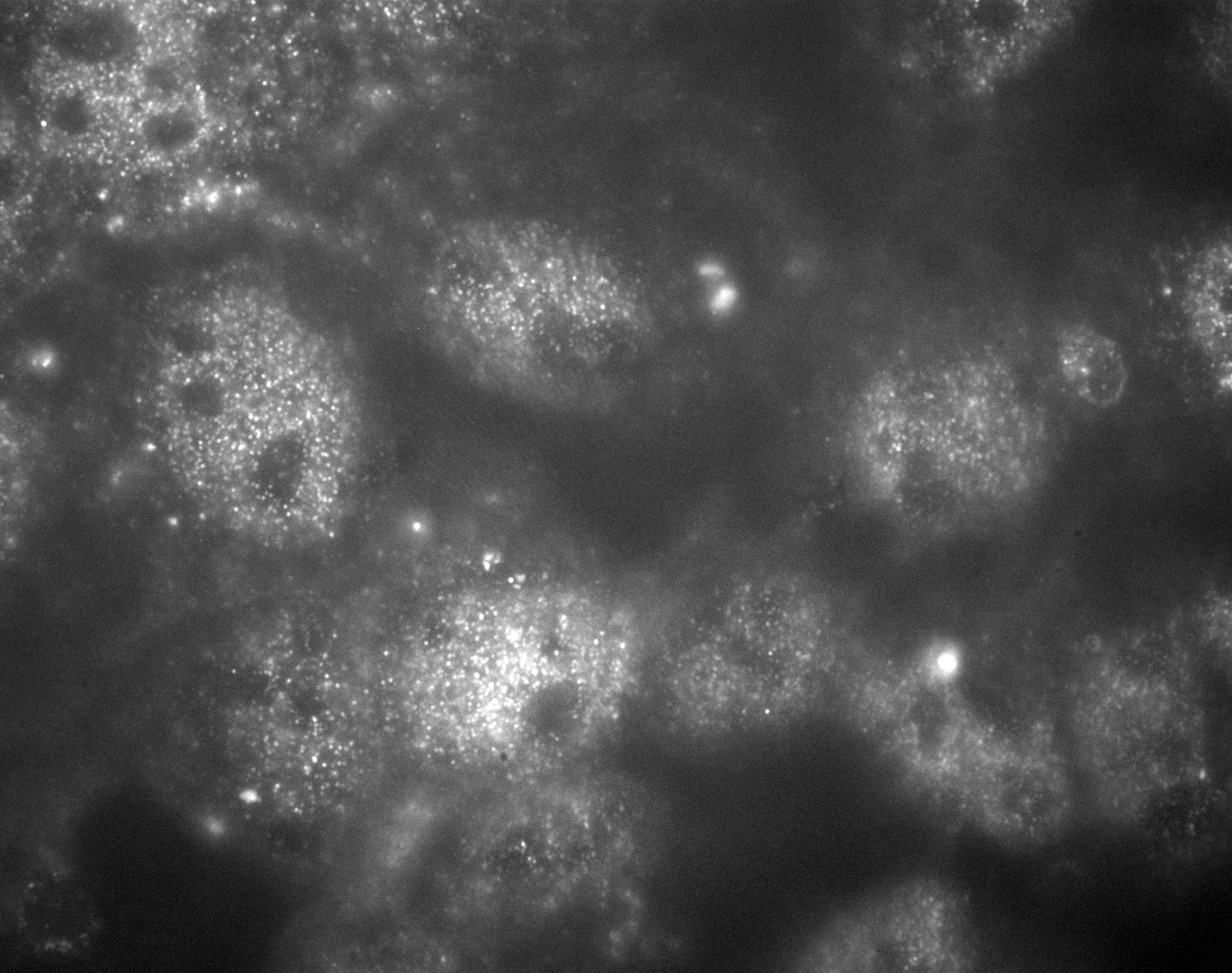

Supplement: Figure 2—source data 1. [file elife-89136-fig2-data1.zip › Figure 2-Source Data/Figure 2-Source Data-2 (raw IF images)/Fig2D-Chol-MCT1-2060-GFP.TIF]

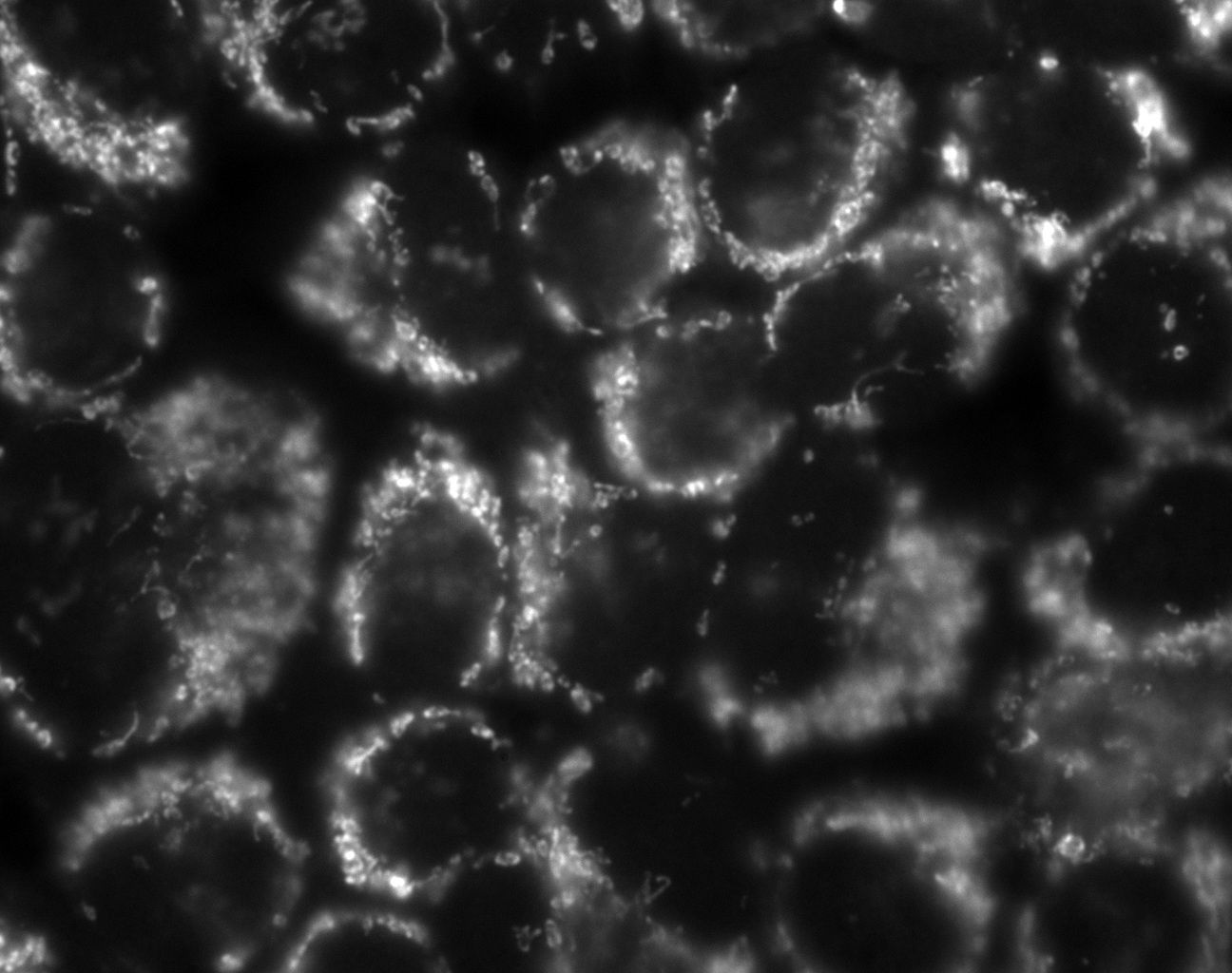

Supplement: Figure 2—source data 1. [file elife-89136-fig2-data1.zip › Figure 2-Source Data/Figure 2-Source Data-2 (raw IF images)/Fig2D-Chol-NTC-siRNA-Cy3.TIF]

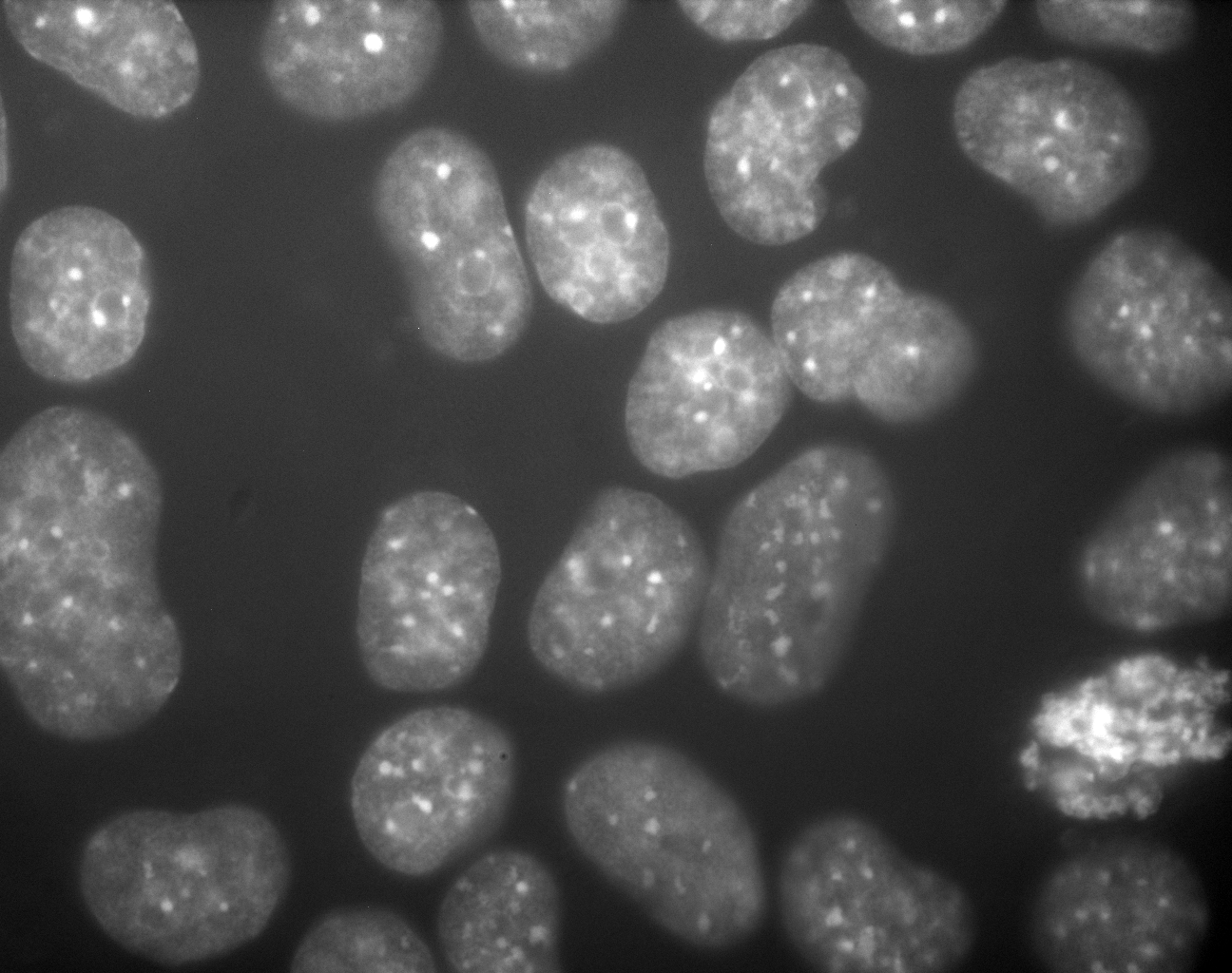

Supplement: Figure 2—source data 1. [file elife-89136-fig2-data1.zip › Figure 2-Source Data/Figure 2-Source Data-2 (raw IF images)/Fig2D-Chol-NTC-siRNA-DAPI.TIF]

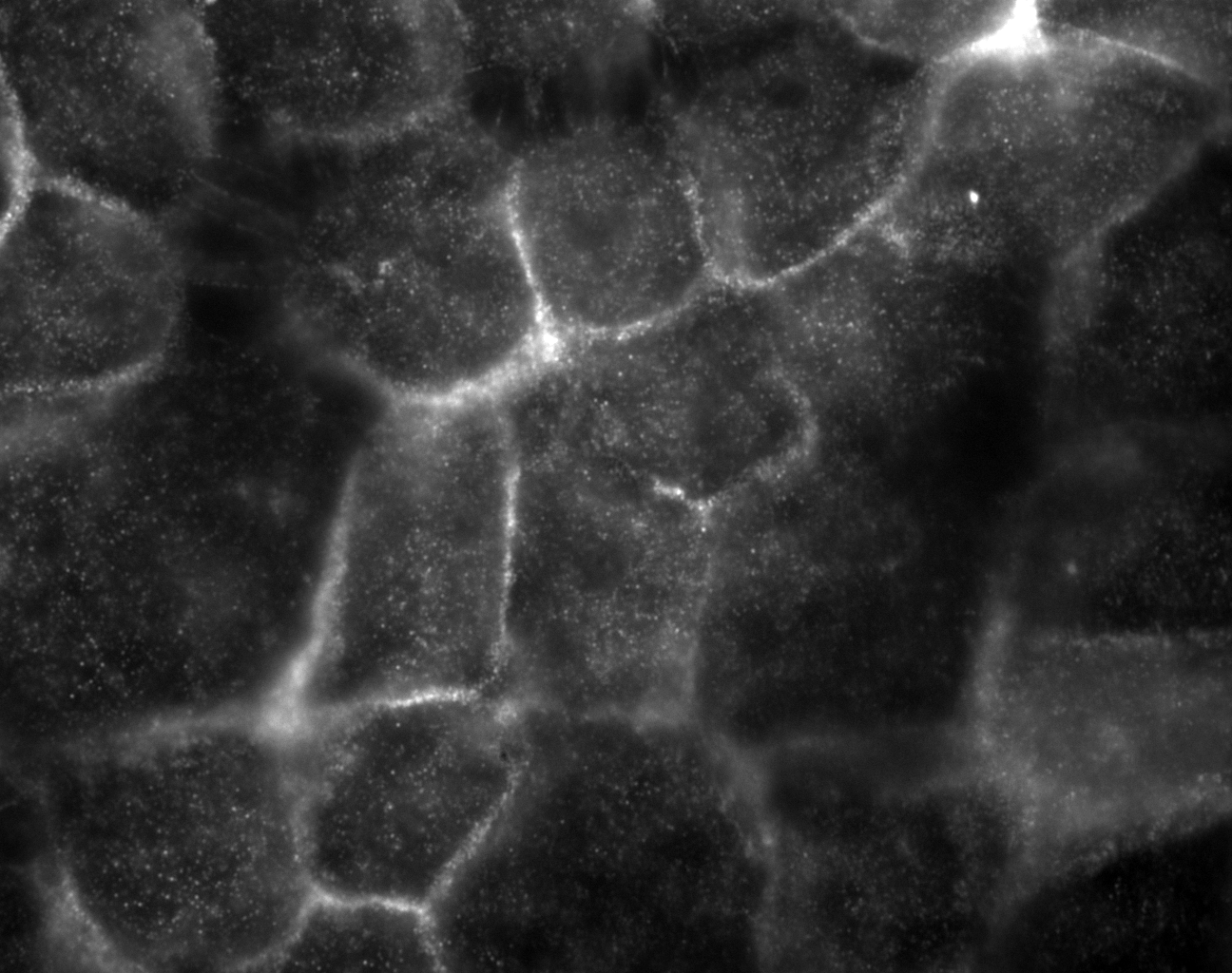

Supplement: Figure 2—source data 1. [file elife-89136-fig2-data1.zip › Figure 2-Source Data/Figure 2-Source Data-2 (raw IF images)/Fig2D-Chol-NTC-siRNA-GFP.TIF]

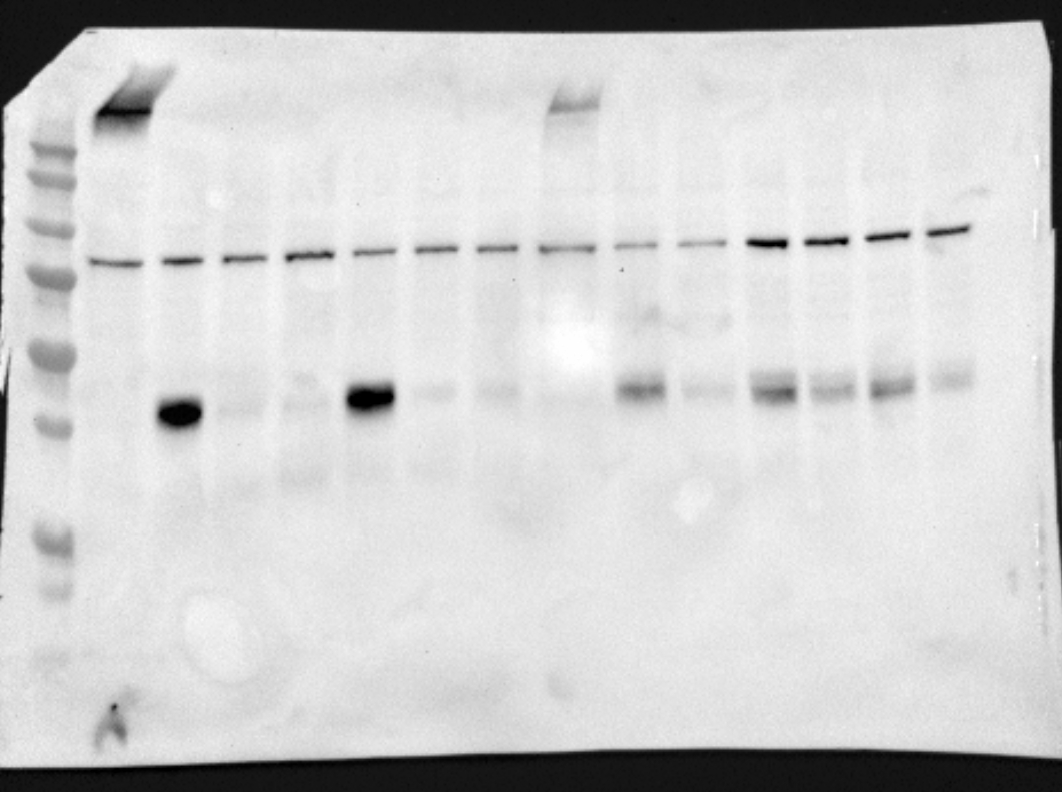

Supplement: Figure 2—source data 1. [file elife-89136-fig2-data1.zip › Figure 2-Source Data/Figure 2-Source Data-4 (raw WB images)/Figure 2E-HSP90.jpg]

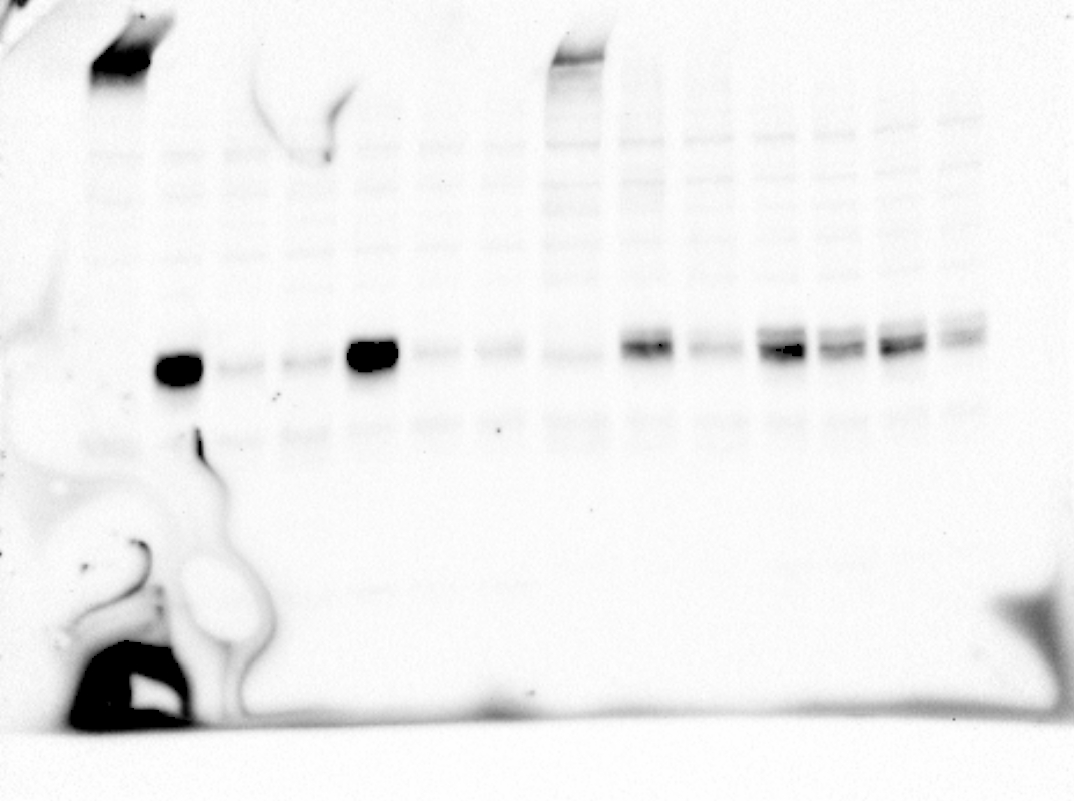

Supplement: Figure 2—source data 1. [file elife-89136-fig2-data1.zip › Figure 2-Source Data/Figure 2-Source Data-4 (raw WB images)/Figure 2E-MCT1.jpg]

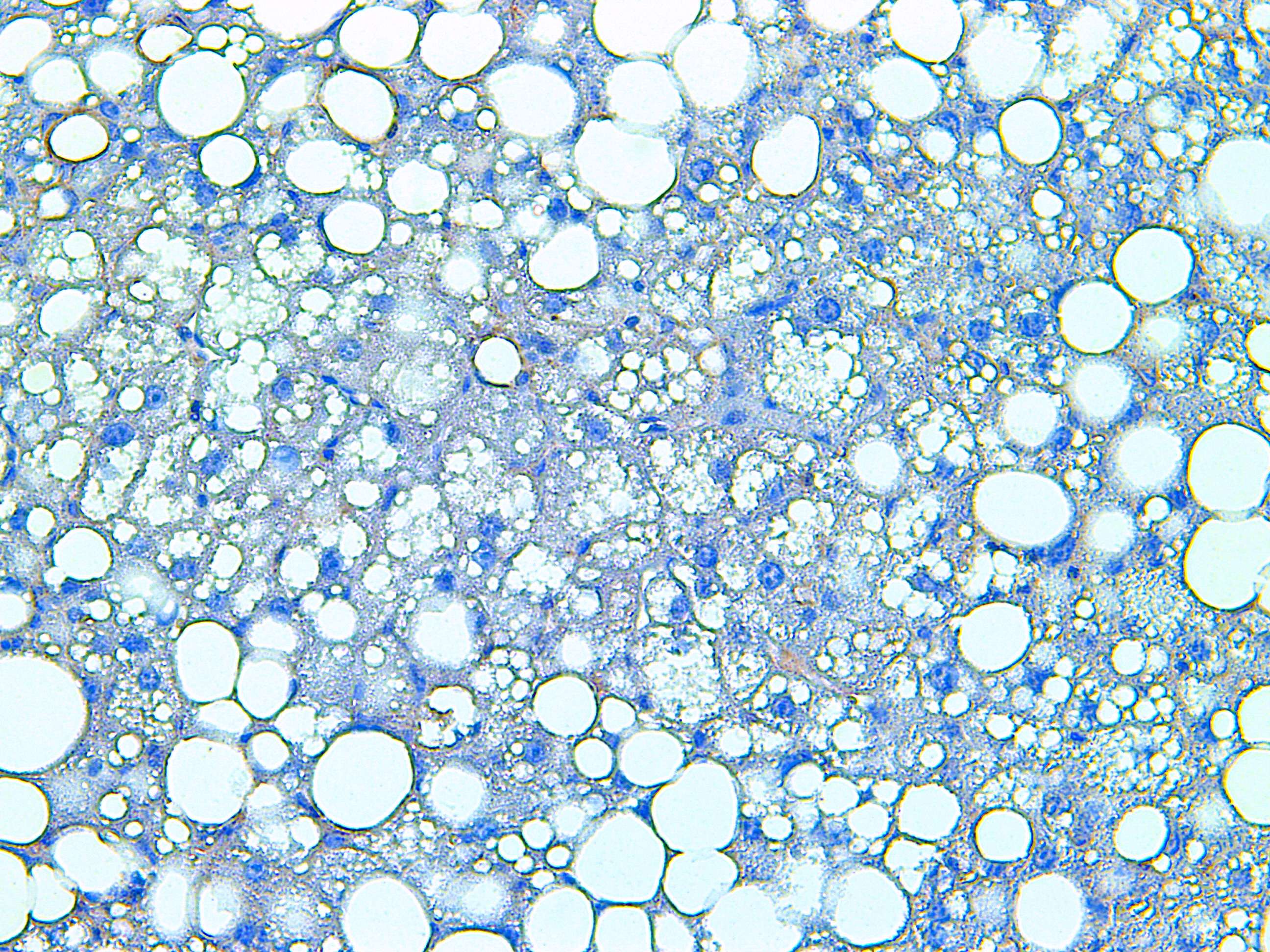

Supplement: Figure 4—source data 1. [file elife-89136-fig4-data1.zip › Figure 4-Source Data/Figure 4-Source Data-1 (raw IHC images)/Figure 4B-Chol-MCT1-siRNA.jpg]

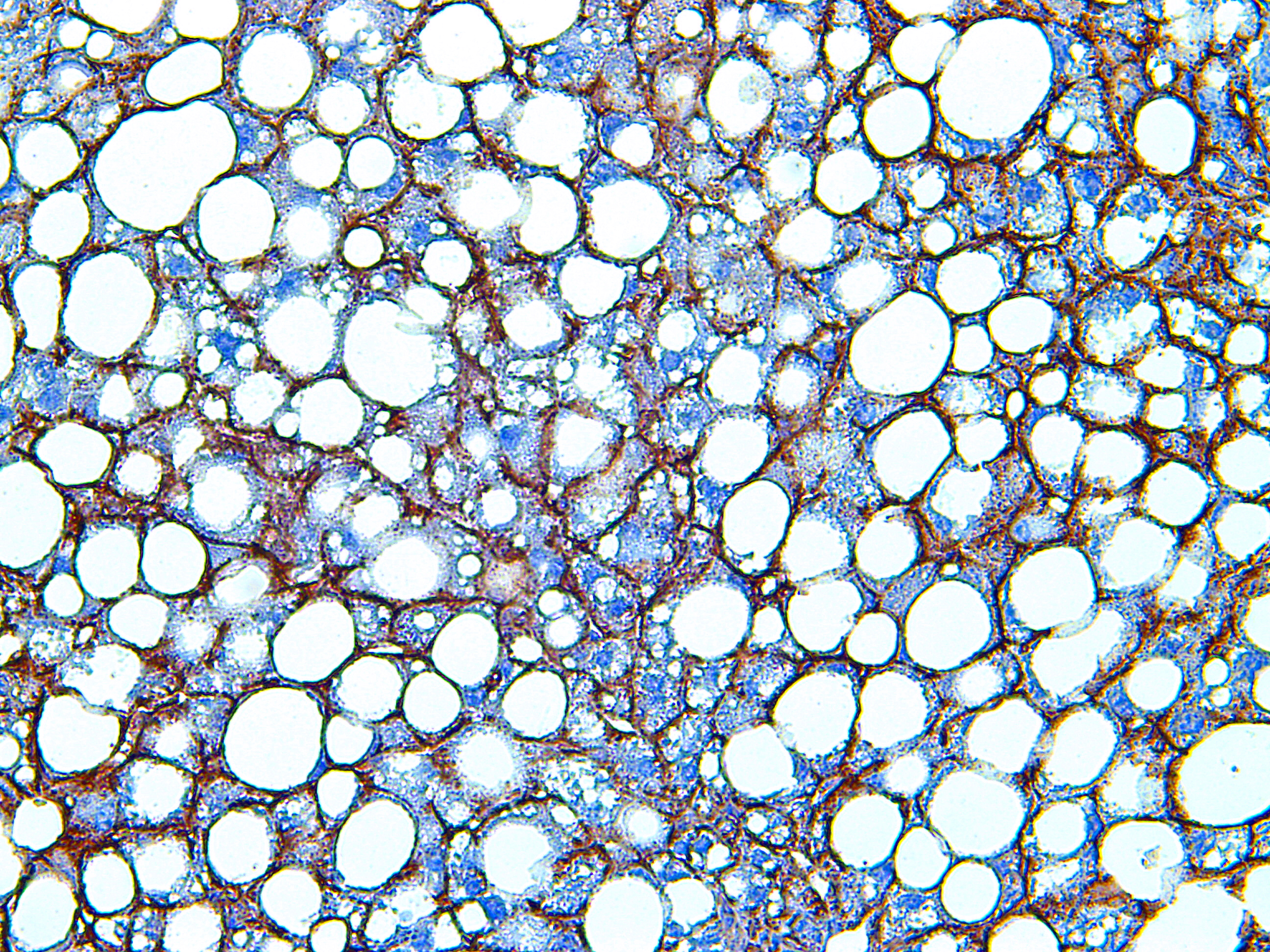

Supplement: Figure 4—source data 1. [file elife-89136-fig4-data1.zip › Figure 4-Source Data/Figure 4-Source Data-1 (raw IHC images)/Figure 4B-Chol-NTC-siRNA.jpg]

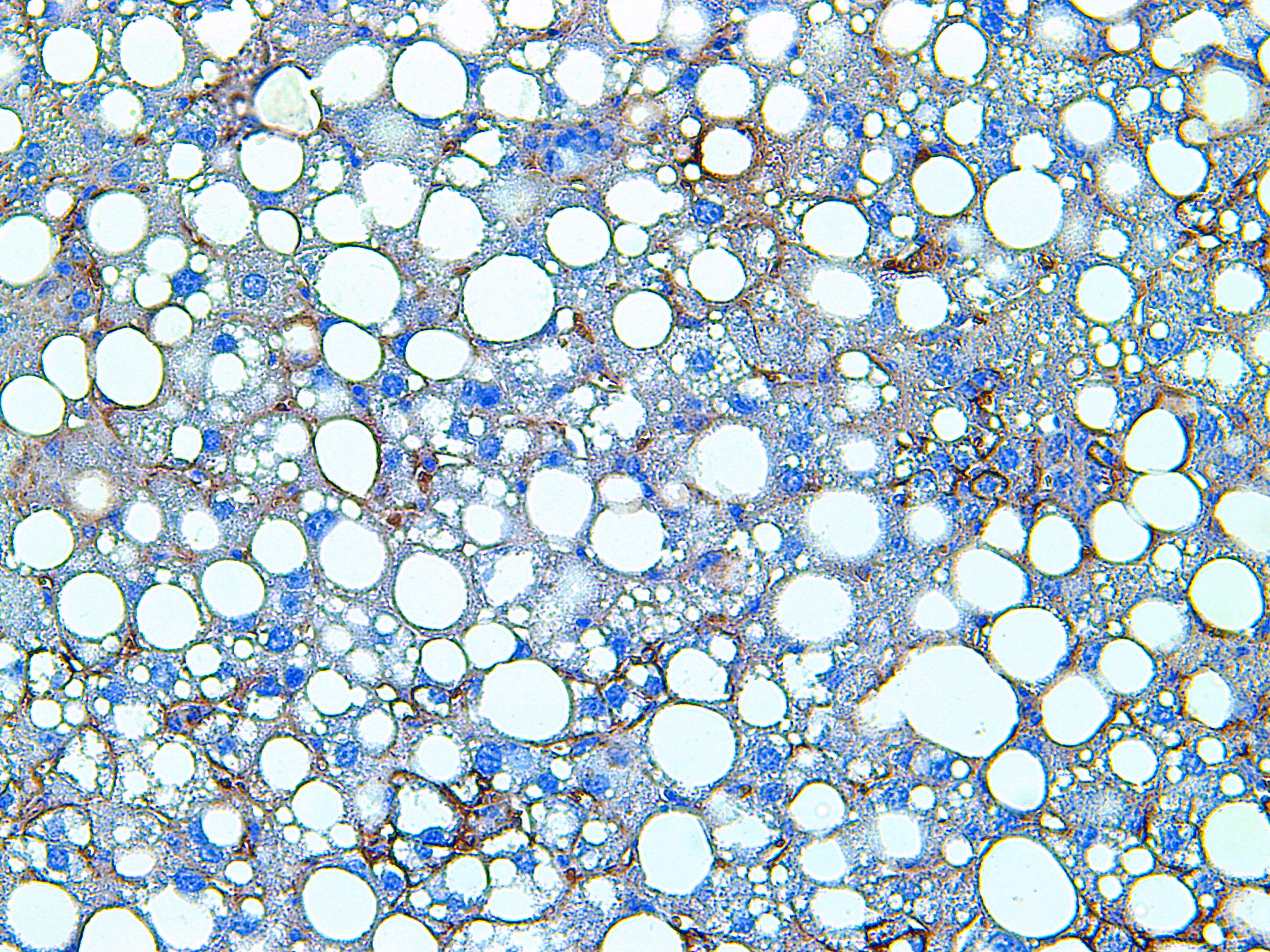

Supplement: Figure 4—source data 1. [file elife-89136-fig4-data1.zip › Figure 4-Source Data/Figure 4-Source Data-1 (raw IHC images)/Figure 4B-GN-MCT1-siRNA.jpg]

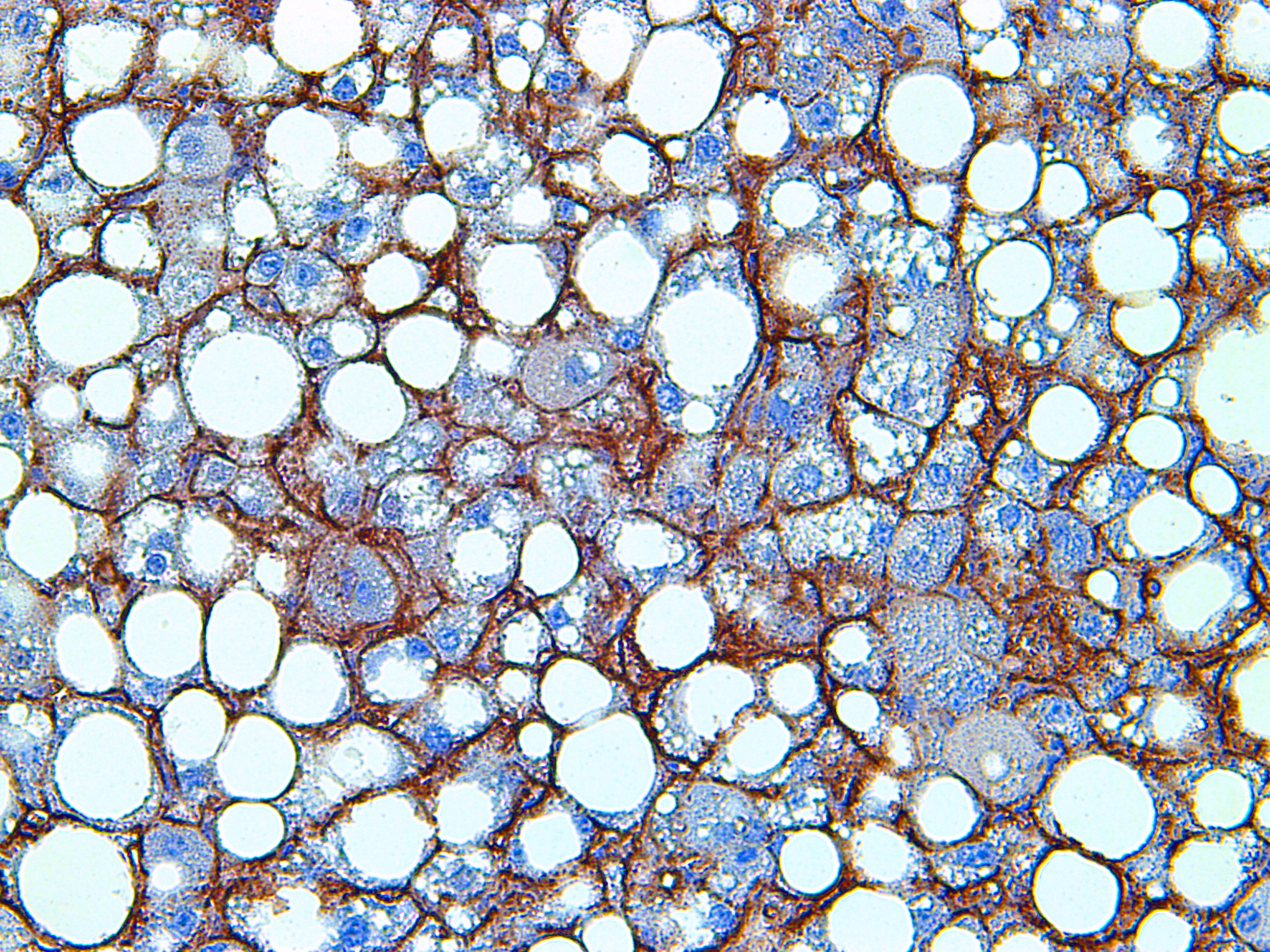

Supplement: Figure 4—source data 1. [file elife-89136-fig4-data1.zip › Figure 4-Source Data/Figure 4-Source Data-1 (raw IHC images)/Figure 4B-GN-NTC-siRNA.jpg]

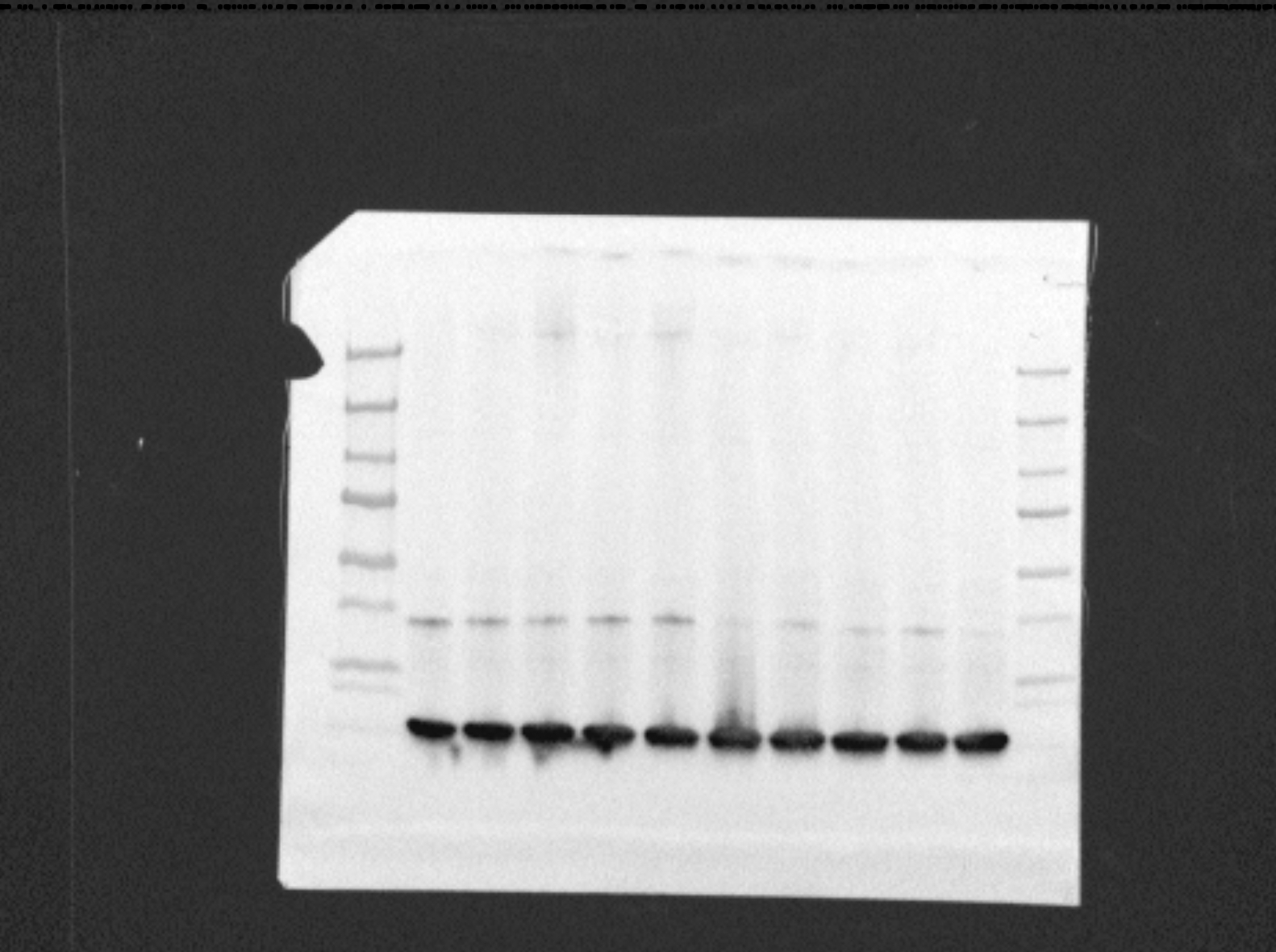

Supplement: Figure 4—figure supplement 2—source data 1. [file elife-89136-fig4-figsupp2-data1.zip › Figure 4-figure supplement 2-Source Data/Figure 4-figure supplement 2-Source Data-2 (raw WB images)/Figure 4-figure supplement 2C-(SCD1)-H3.jpg]

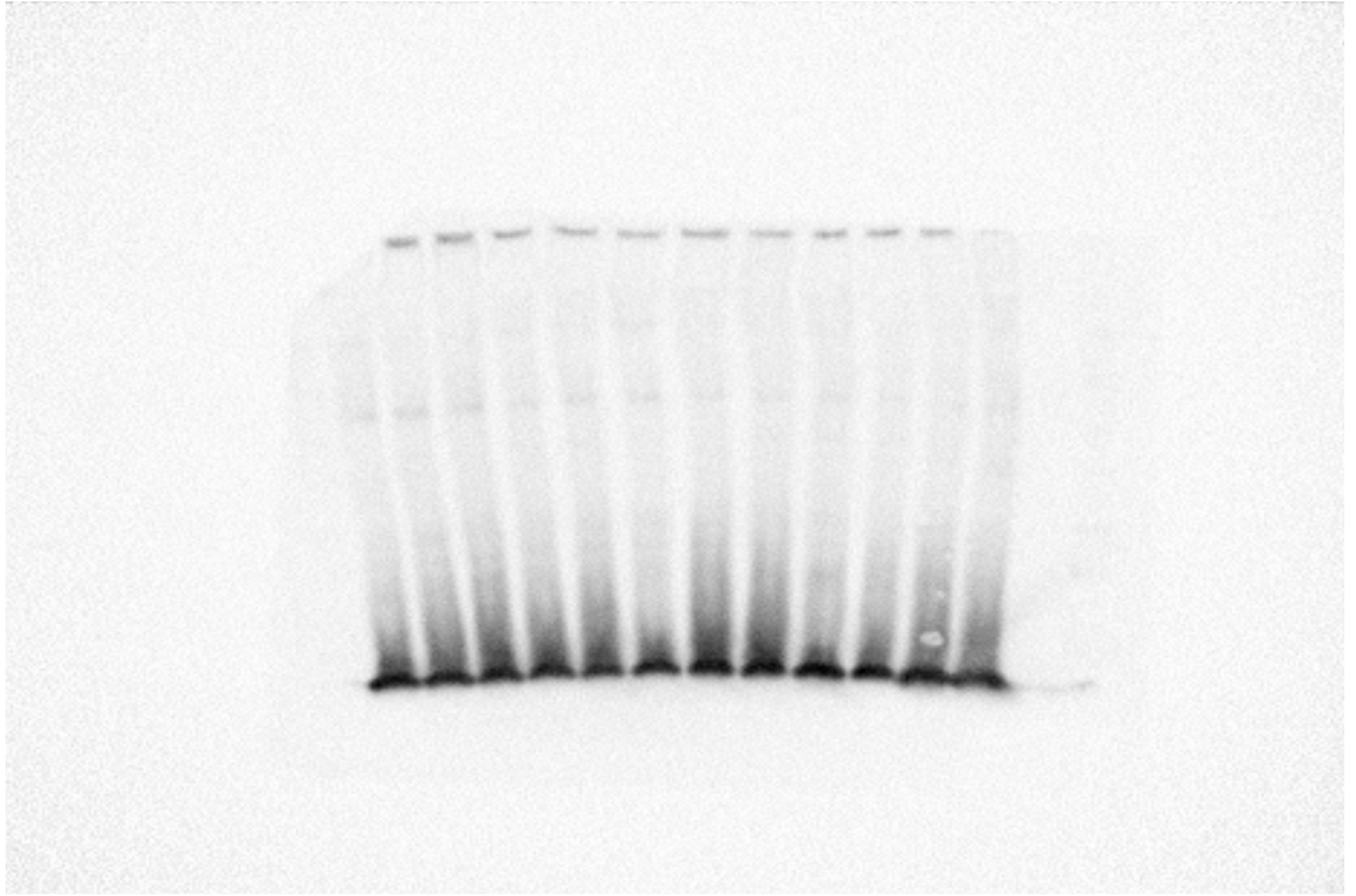

Supplement: Figure 4—figure supplement 2—source data 1. [file elife-89136-fig4-figsupp2-data1.zip › Figure 4-figure supplement 2-Source Data/Figure 4-figure supplement 2-Source Data-2 (raw WB images)/Figure 4-figure supplement 2C-(SREBP1)-H3.jpg]

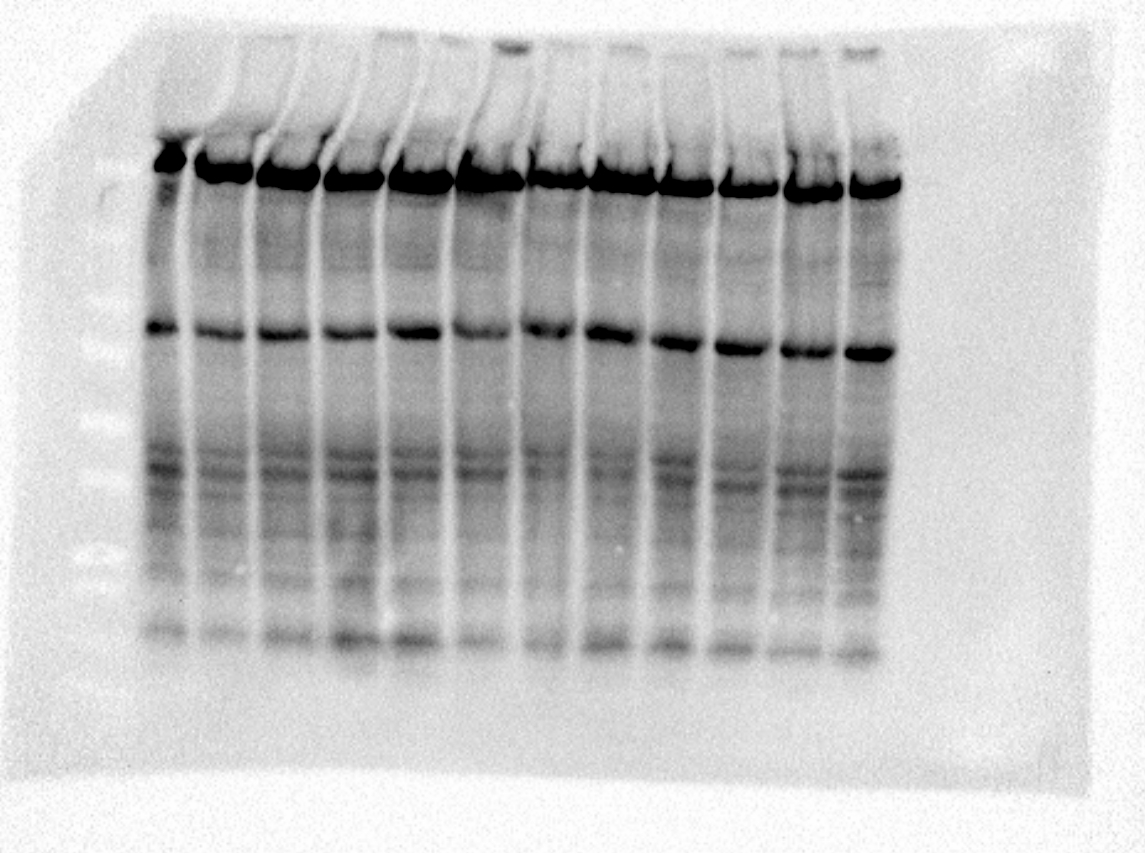

Supplement: Figure 4—figure supplement 2—source data 1. [file elife-89136-fig4-figsupp2-data1.zip › Figure 4-figure supplement 2-Source Data/Figure 4-figure supplement 2-Source Data-2 (raw WB images)/Figure 4-figure supplement 2C-ACLY (alternative uncropped image).jpg]

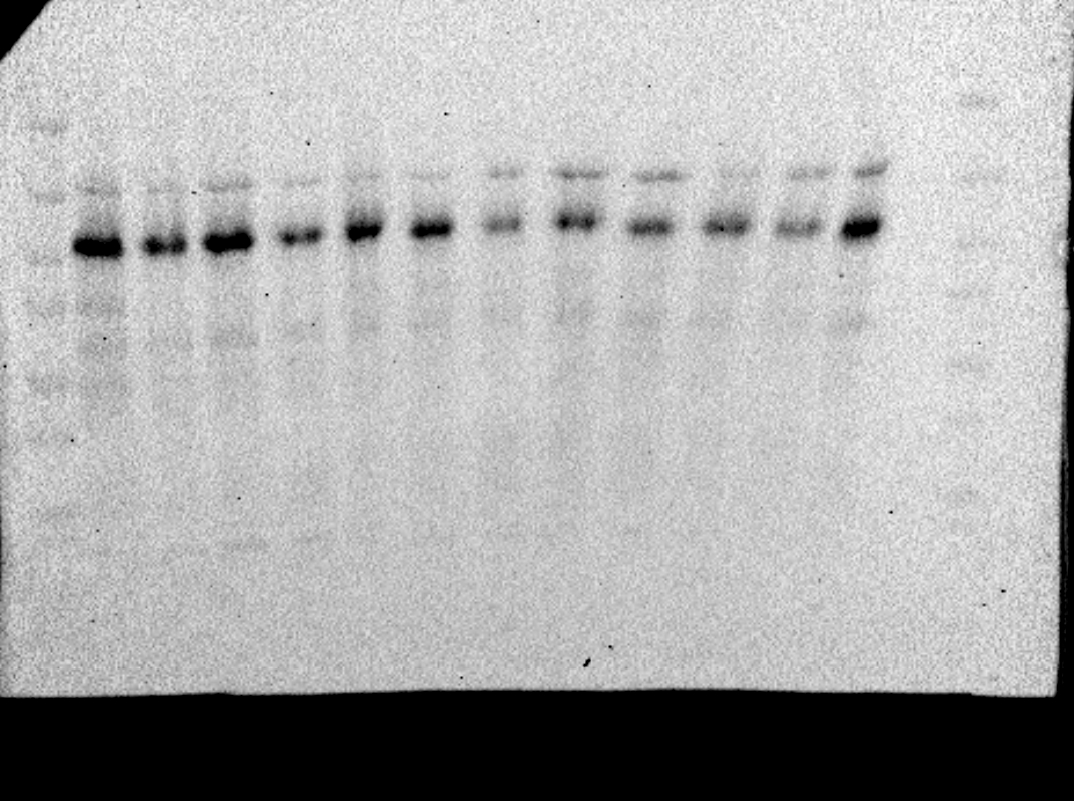

Supplement: Figure 4—figure supplement 2—source data 1. [file elife-89136-fig4-figsupp2-data1.zip › Figure 4-figure supplement 2-Source Data/Figure 4-figure supplement 2-Source Data-2 (raw WB images)/Figure 4-figure supplement 2C-ChREBP.jpg]

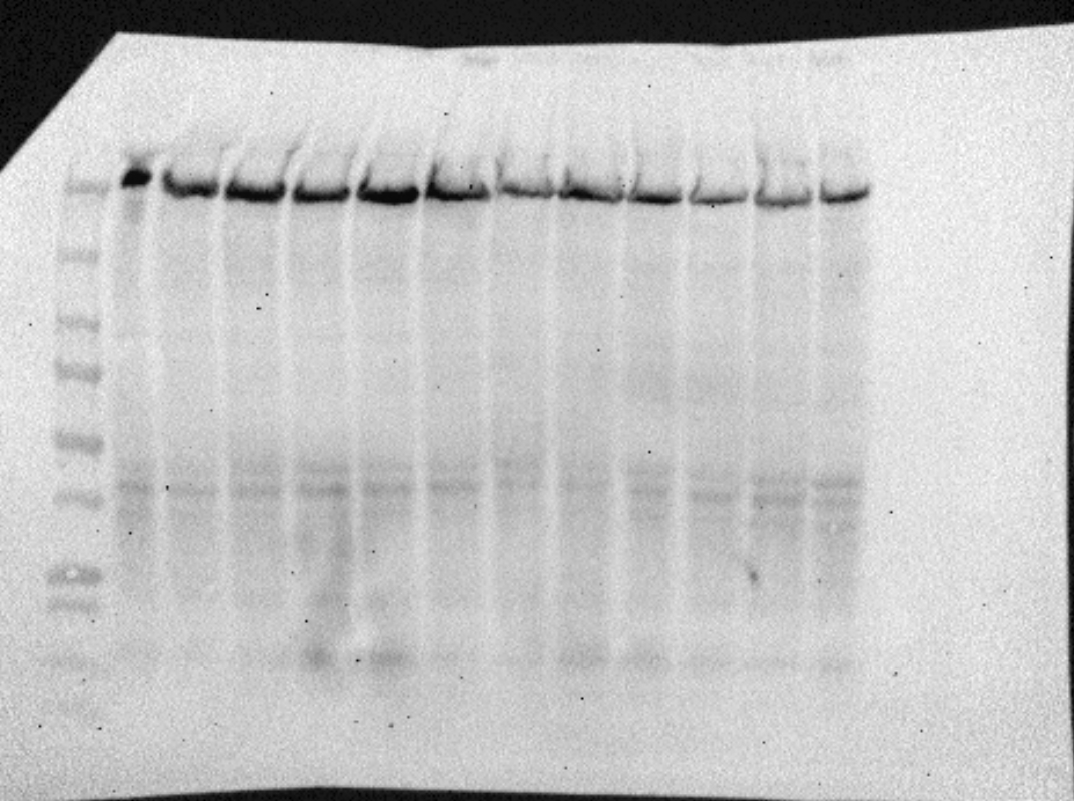

Supplement: Figure 4—figure supplement 2—source data 1. [file elife-89136-fig4-figsupp2-data1.zip › Figure 4-figure supplement 2-Source Data/Figure 4-figure supplement 2-Source Data-2 (raw WB images)/Figure 4-figure supplement 2C-FASN (alternative uncropped image).jpg]

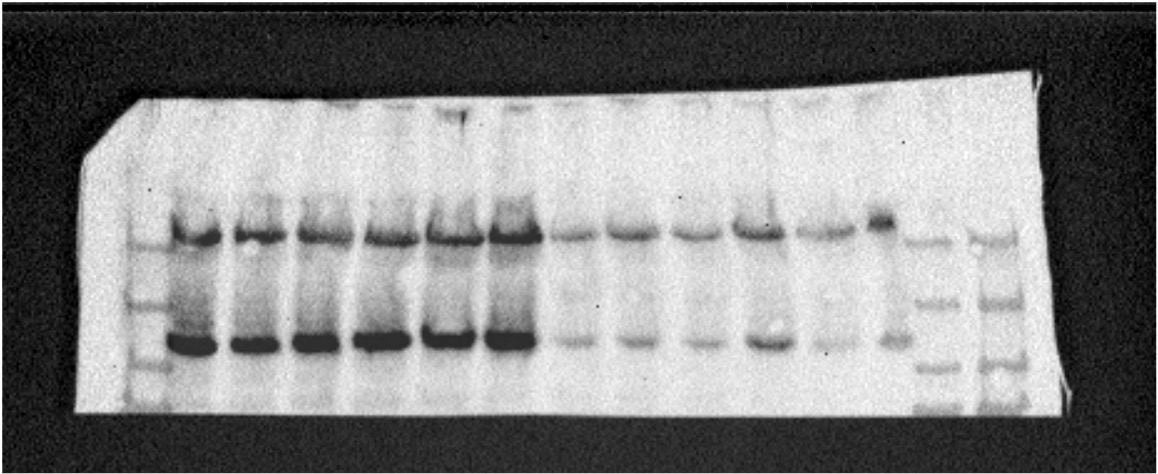

Supplement: Figure 4—figure supplement 2—source data 1. [file elife-89136-fig4-figsupp2-data1.zip › Figure 4-figure supplement 2-Source Data/Figure 4-figure supplement 2-Source Data-2 (raw WB images)/Figure 4-figure supplement 2C-FASN-ACLY (Cropped).jpg]

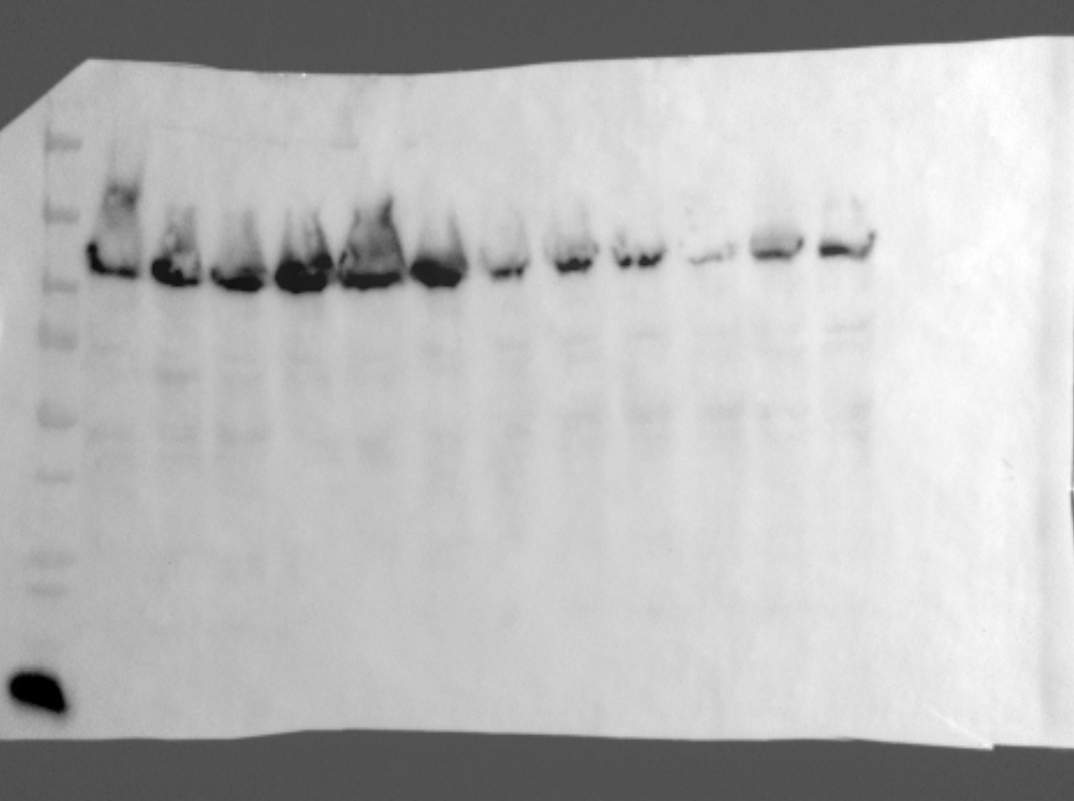

Supplement: Figure 4—figure supplement 2—source data 1. [file elife-89136-fig4-figsupp2-data1.zip › Figure 4-figure supplement 2-Source Data/Figure 4-figure supplement 2-Source Data-2 (raw WB images)/Figure 4-figure supplement 2C-HSP90 (alternative uncropped image).jpg]

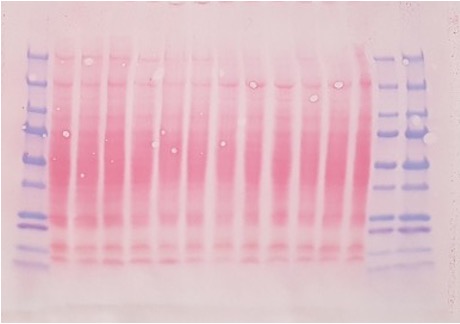

Supplement: Figure 4—figure supplement 2—source data 1. [file elife-89136-fig4-figsupp2-data1.zip › Figure 4-figure supplement 2-Source Data/Figure 4-figure supplement 2-Source Data-2 (raw WB images)/Figure 4-figure supplement 2C-ponceau (Uncropped).jpg]

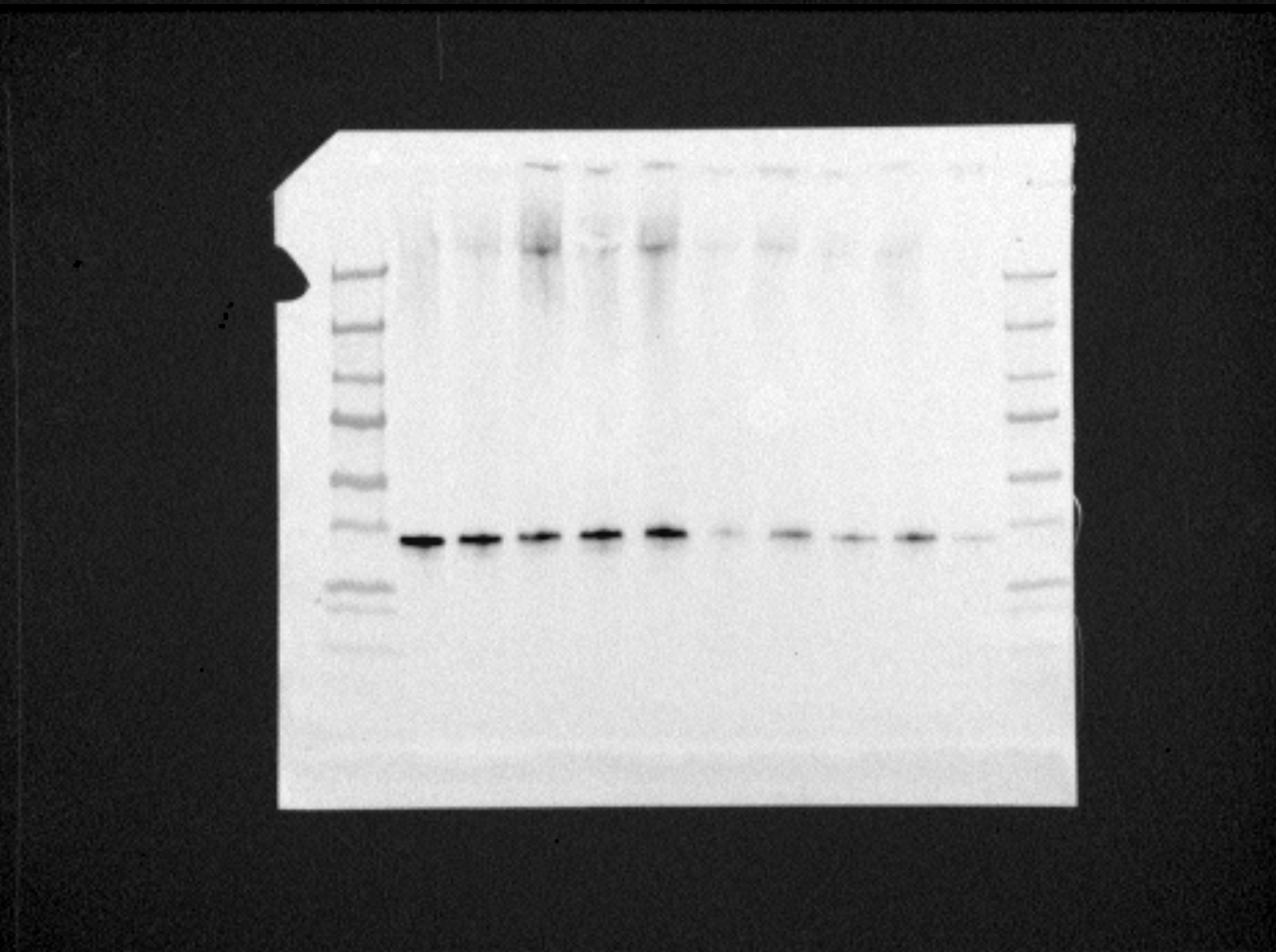

Supplement: Figure 4—figure supplement 2—source data 1. [file elife-89136-fig4-figsupp2-data1.zip › Figure 4-figure supplement 2-Source Data/Figure 4-figure supplement 2-Source Data-2 (raw WB images)/Figure 4-figure supplement 2C-SCD1.jpg]

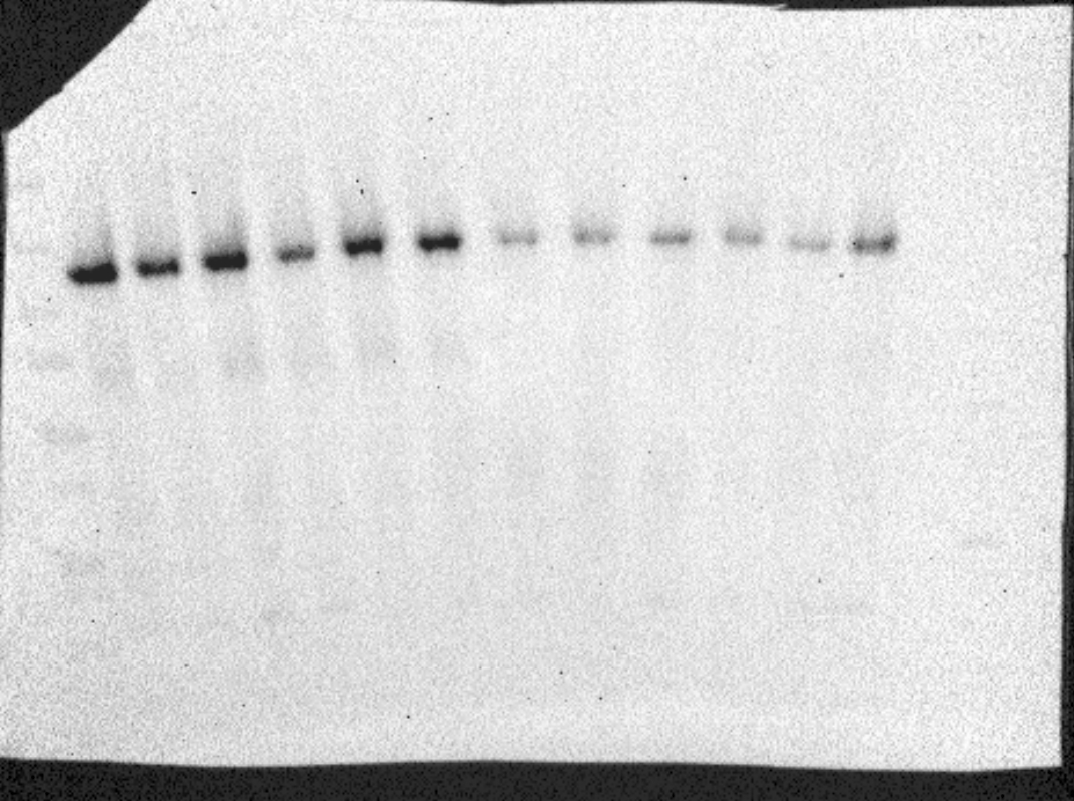

Supplement: Figure 4—figure supplement 2—source data 1. [file elife-89136-fig4-figsupp2-data1.zip › Figure 4-figure supplement 2-Source Data/Figure 4-figure supplement 2-Source Data-2 (raw WB images)/Figure 4-figure supplement 2C-SREBP1.jpg]

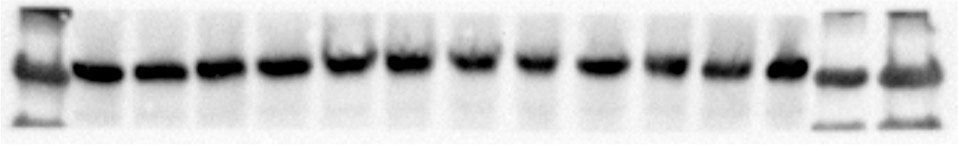

Supplement: Figure 4—figure supplement 2—source data 1. [file elife-89136-fig4-figsupp2-data1.zip › Figure 4-figure supplement 2-Source Data/Figure 4-figure supplement 2-Source Data-2 (raw WB images)/Figure 4-figure supplement 2C-Tubulin (Cropped).jpg]

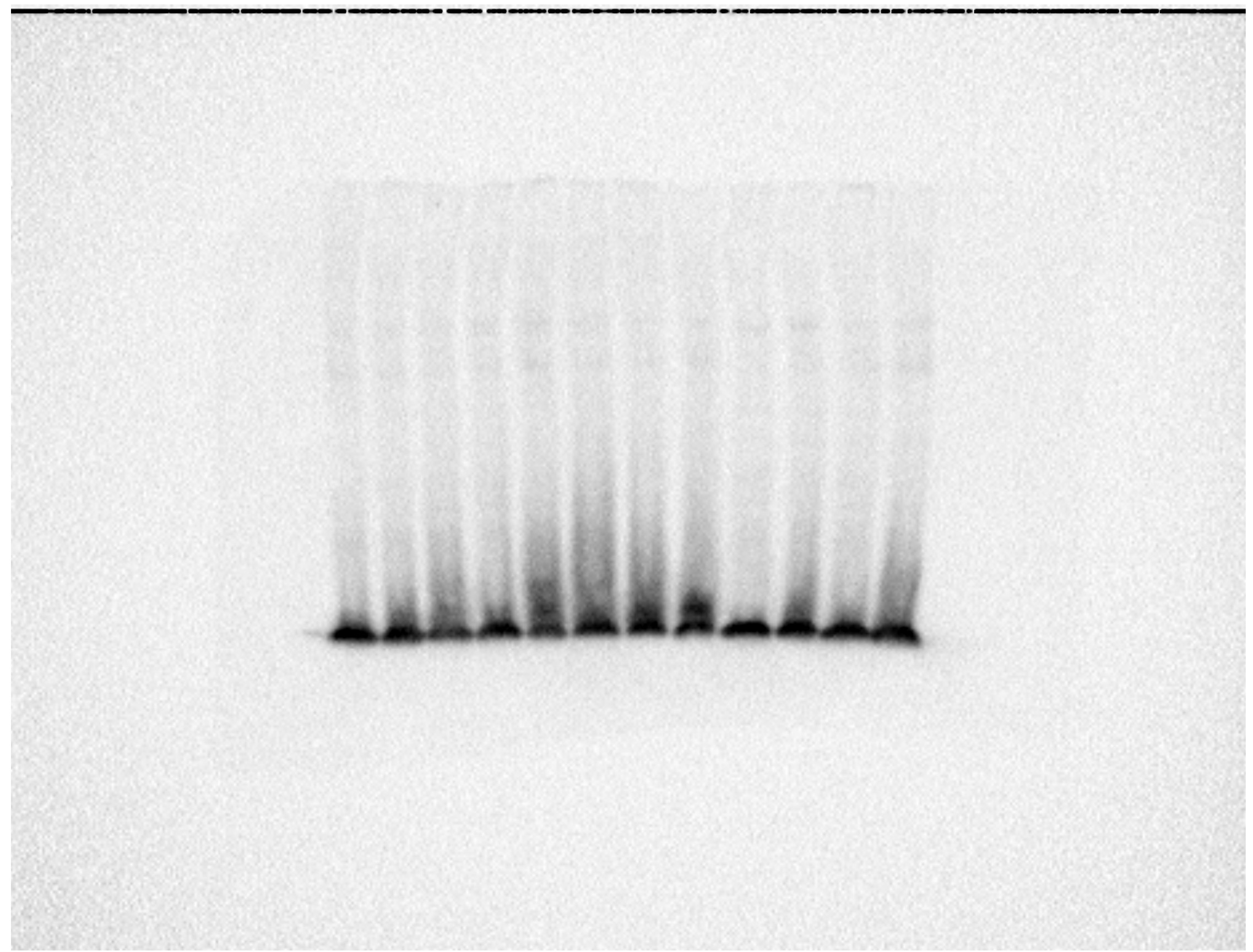

Supplement: Figure 4—figure supplement 2—source data 1. [file elife-89136-fig4-figsupp2-data1.zip › Figure 4-figure supplement 2-Source Data/Figure 4-figure supplement 2-Source Data-2 (raw WB images)/Figure 4-figure supplement 2D-(ChREBP)-H3.jpg]

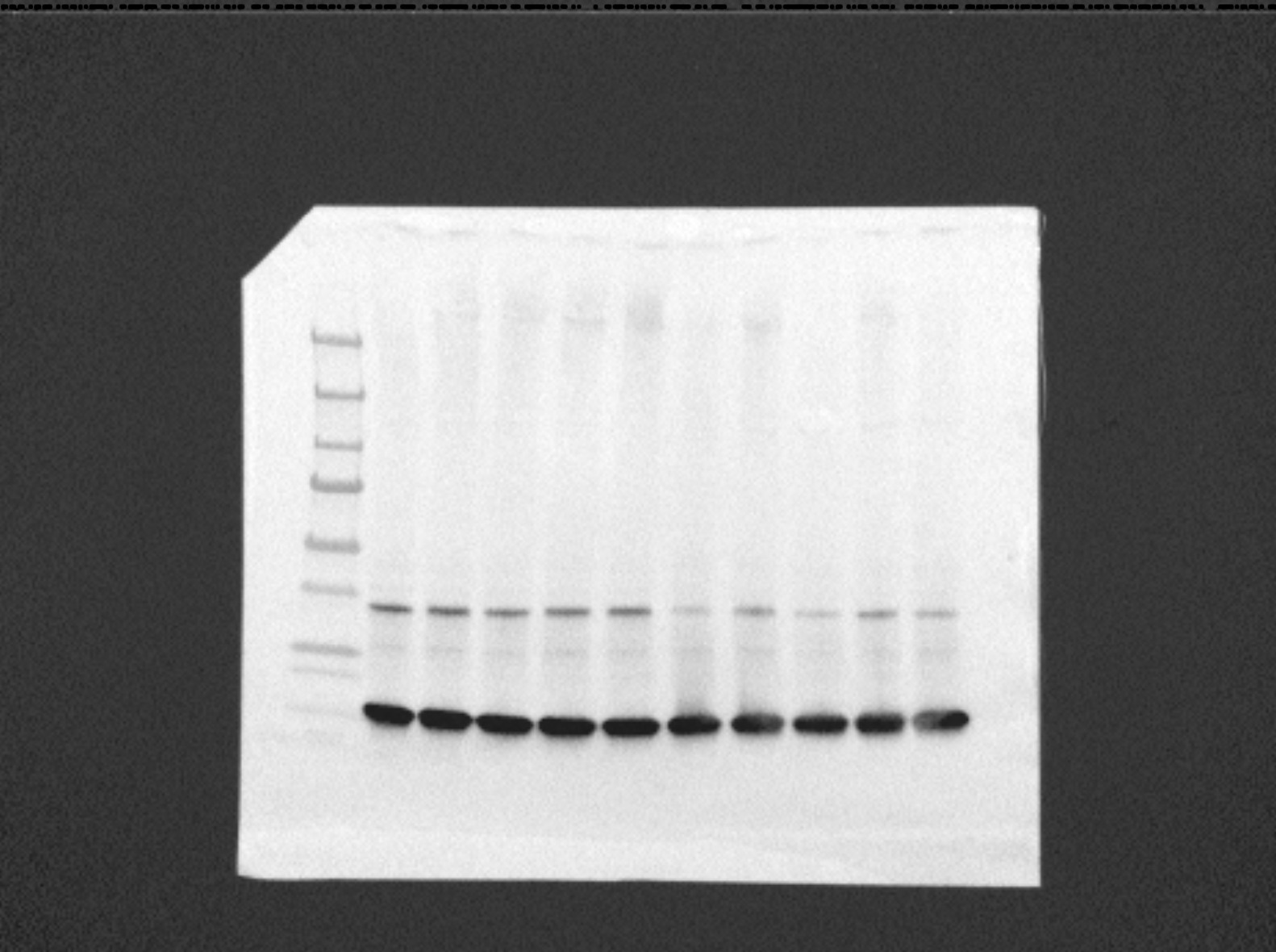

Supplement: Figure 4—figure supplement 2—source data 1. [file elife-89136-fig4-figsupp2-data1.zip › Figure 4-figure supplement 2-Source Data/Figure 4-figure supplement 2-Source Data-2 (raw WB images)/Figure 4-figure supplement 2D-(SCD)-H3.jpg]

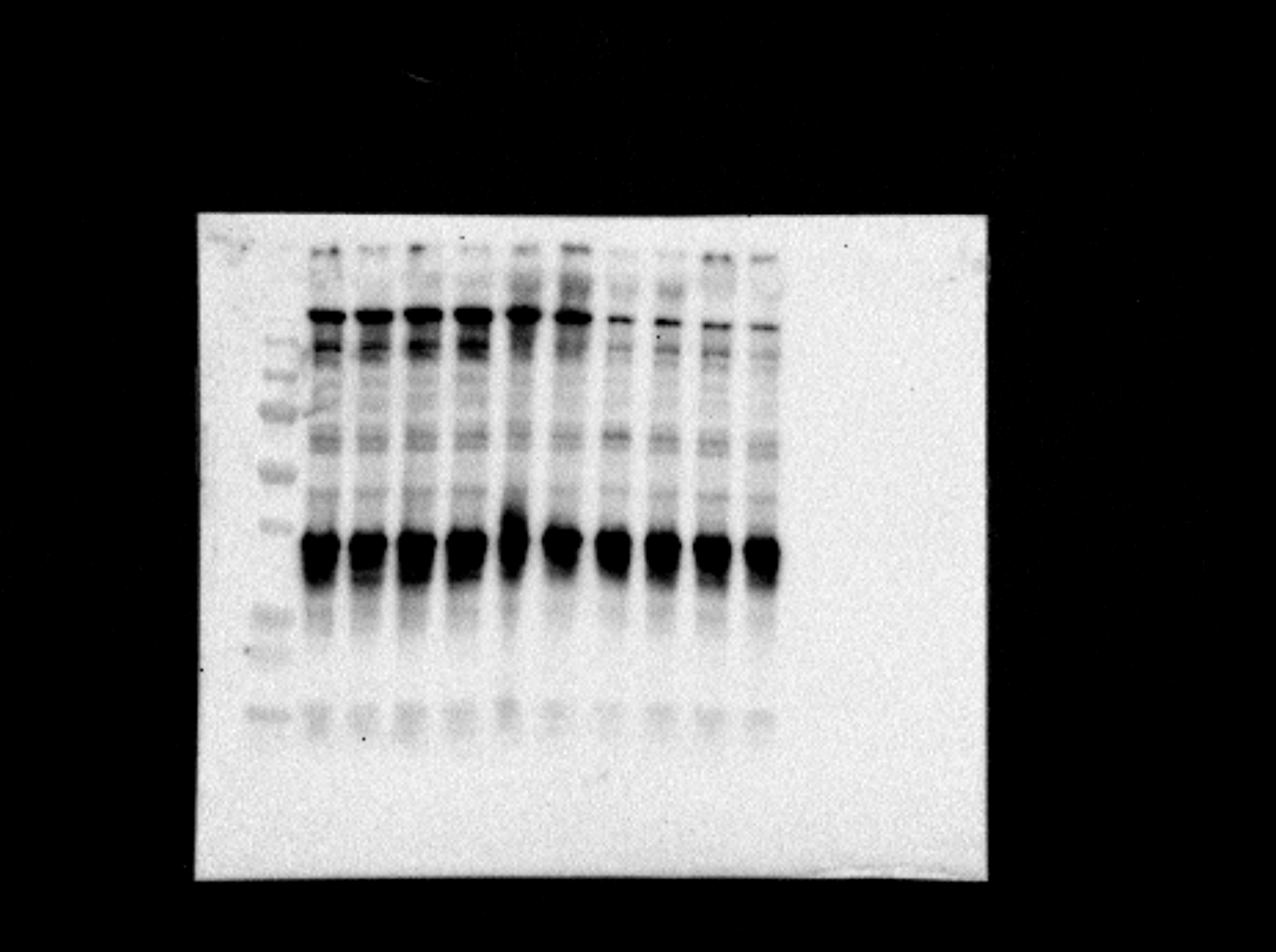

Supplement: Figure 4—figure supplement 2—source data 1. [file elife-89136-fig4-figsupp2-data1.zip › Figure 4-figure supplement 2-Source Data/Figure 4-figure supplement 2-Source Data-2 (raw WB images)/Figure 4-figure supplement 2D-ACLY.jpg]

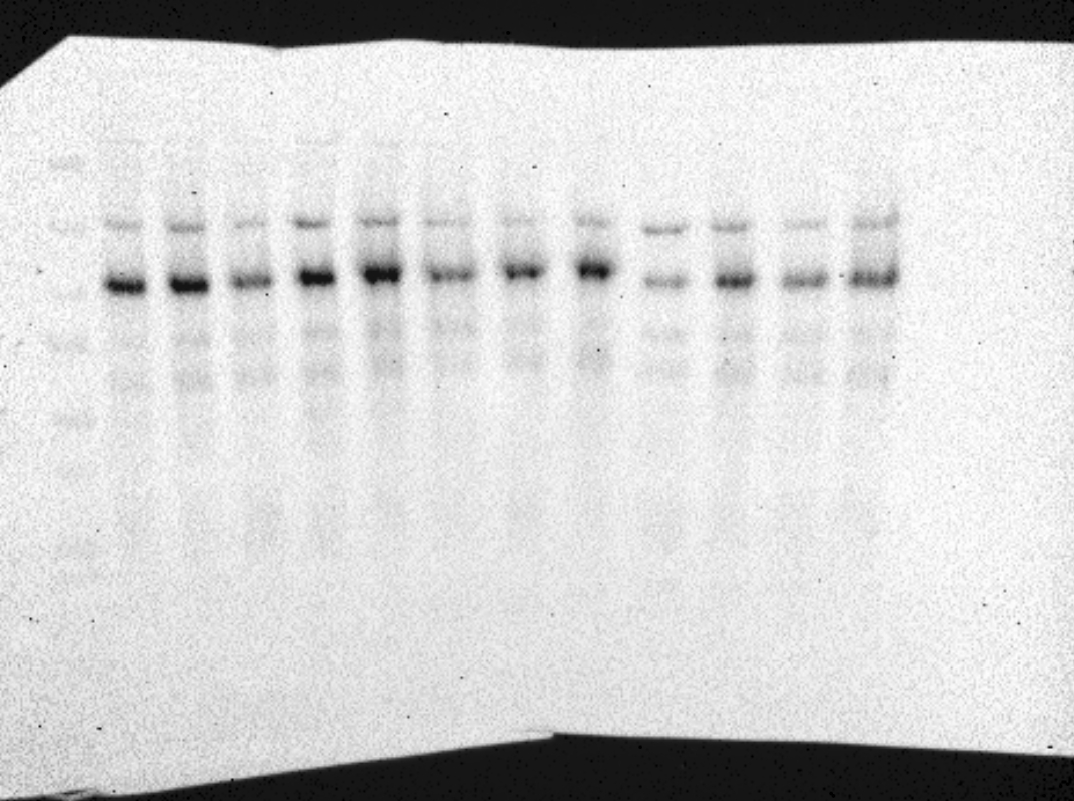

Supplement: Figure 4—figure supplement 2—source data 1. [file elife-89136-fig4-figsupp2-data1.zip › Figure 4-figure supplement 2-Source Data/Figure 4-figure supplement 2-Source Data-2 (raw WB images)/Figure 4-figure supplement 2D-ChREBP.jpg]

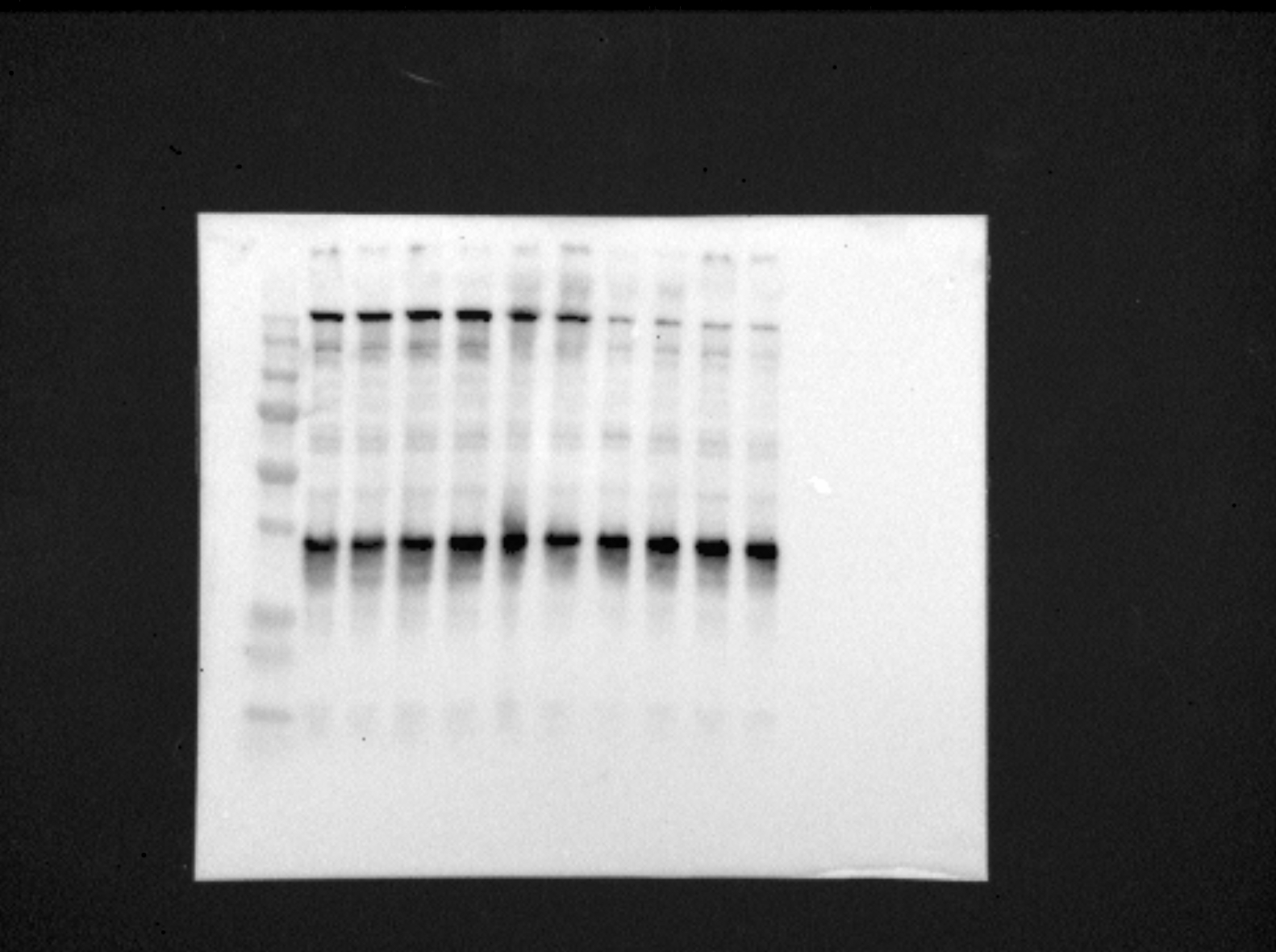

Supplement: Figure 4—figure supplement 2—source data 1. [file elife-89136-fig4-figsupp2-data1.zip › Figure 4-figure supplement 2-Source Data/Figure 4-figure supplement 2-Source Data-2 (raw WB images)/Figure 4-figure supplement 2D-FASN-GAPDH.jpg]

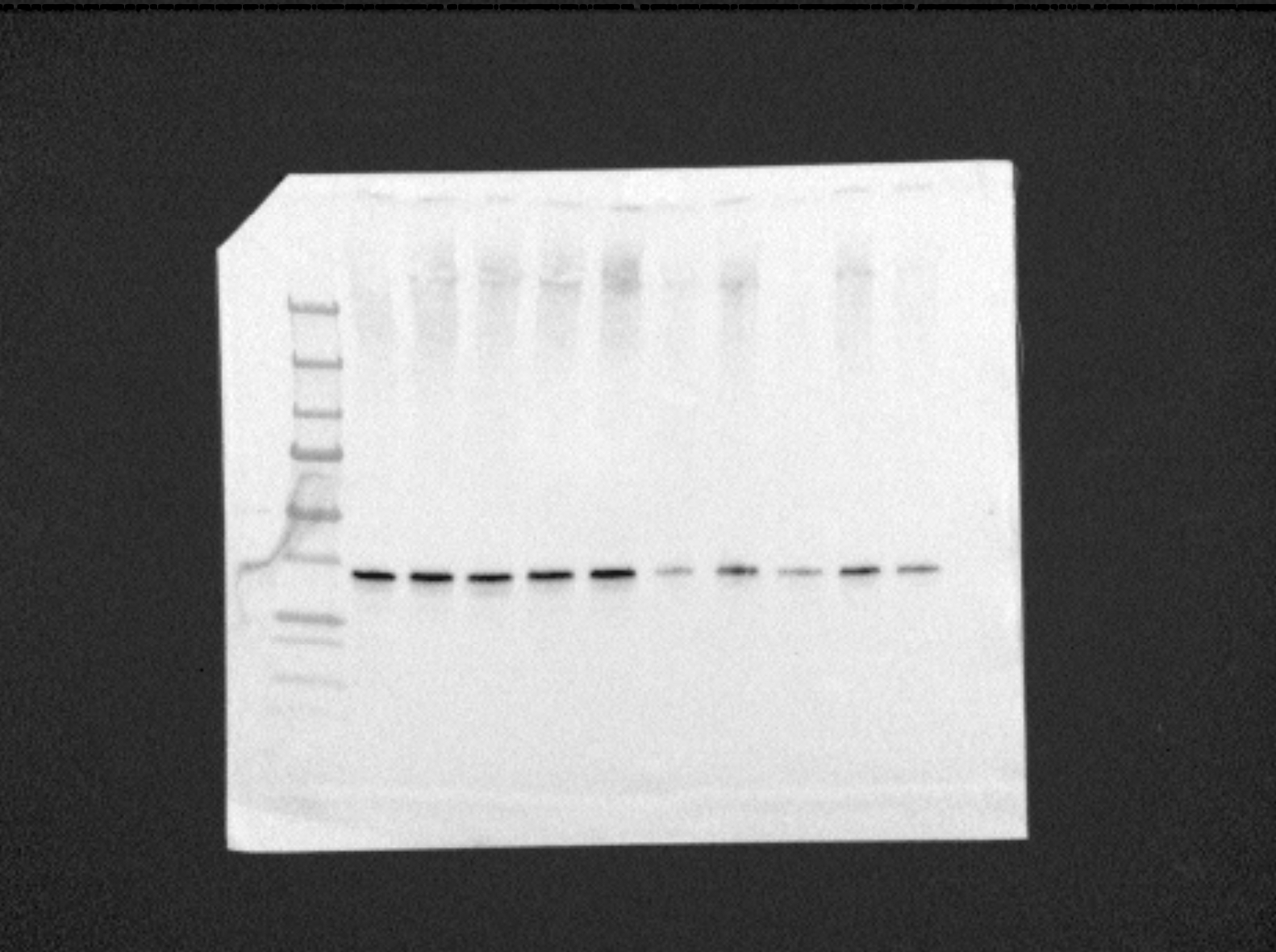

Supplement: Figure 4—figure supplement 2—source data 1. [file elife-89136-fig4-figsupp2-data1.zip › Figure 4-figure supplement 2-Source Data/Figure 4-figure supplement 2-Source Data-2 (raw WB images)/Figure 4-figure supplement 2D-SCD1.jpg]

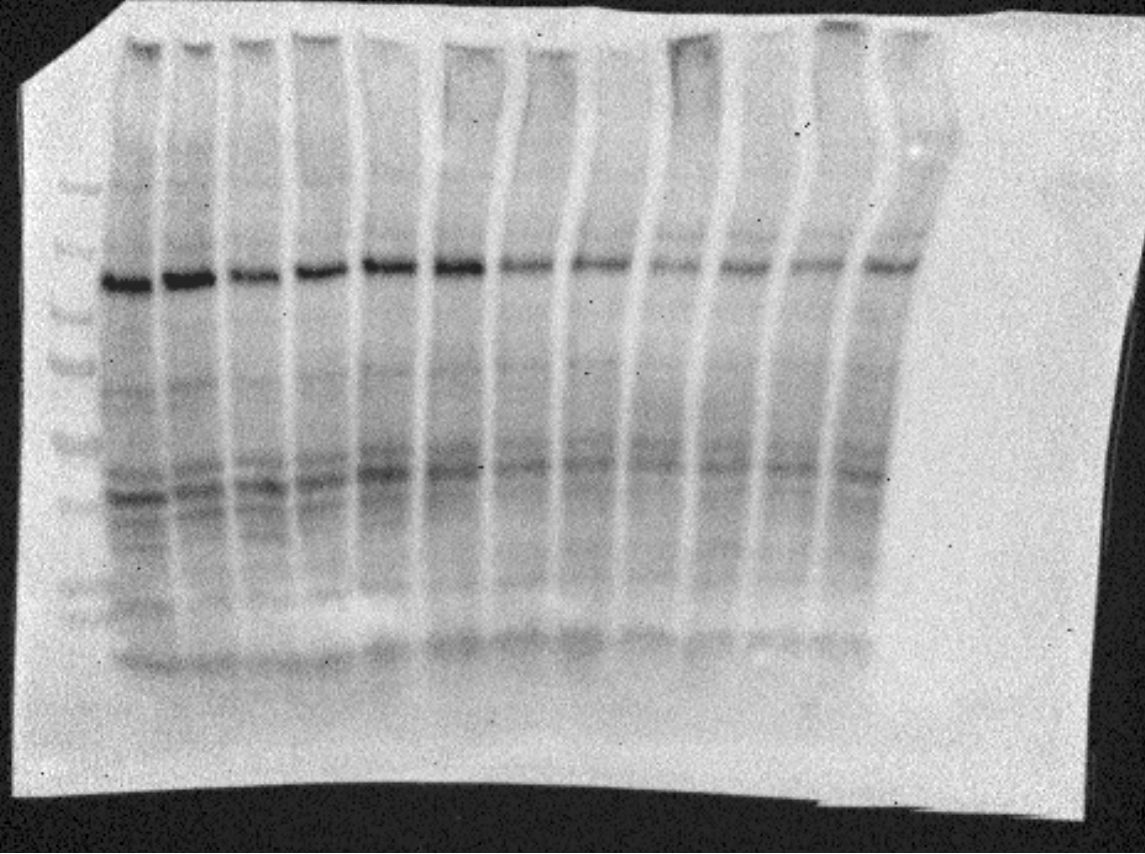

Supplement: Figure 4—figure supplement 2—source data 1. [file elife-89136-fig4-figsupp2-data1.zip › Figure 4-figure supplement 2-Source Data/Figure 4-figure supplement 2-Source Data-2 (raw WB images)/Figure 4-figure supplement 2D-SREBP1.jpg]

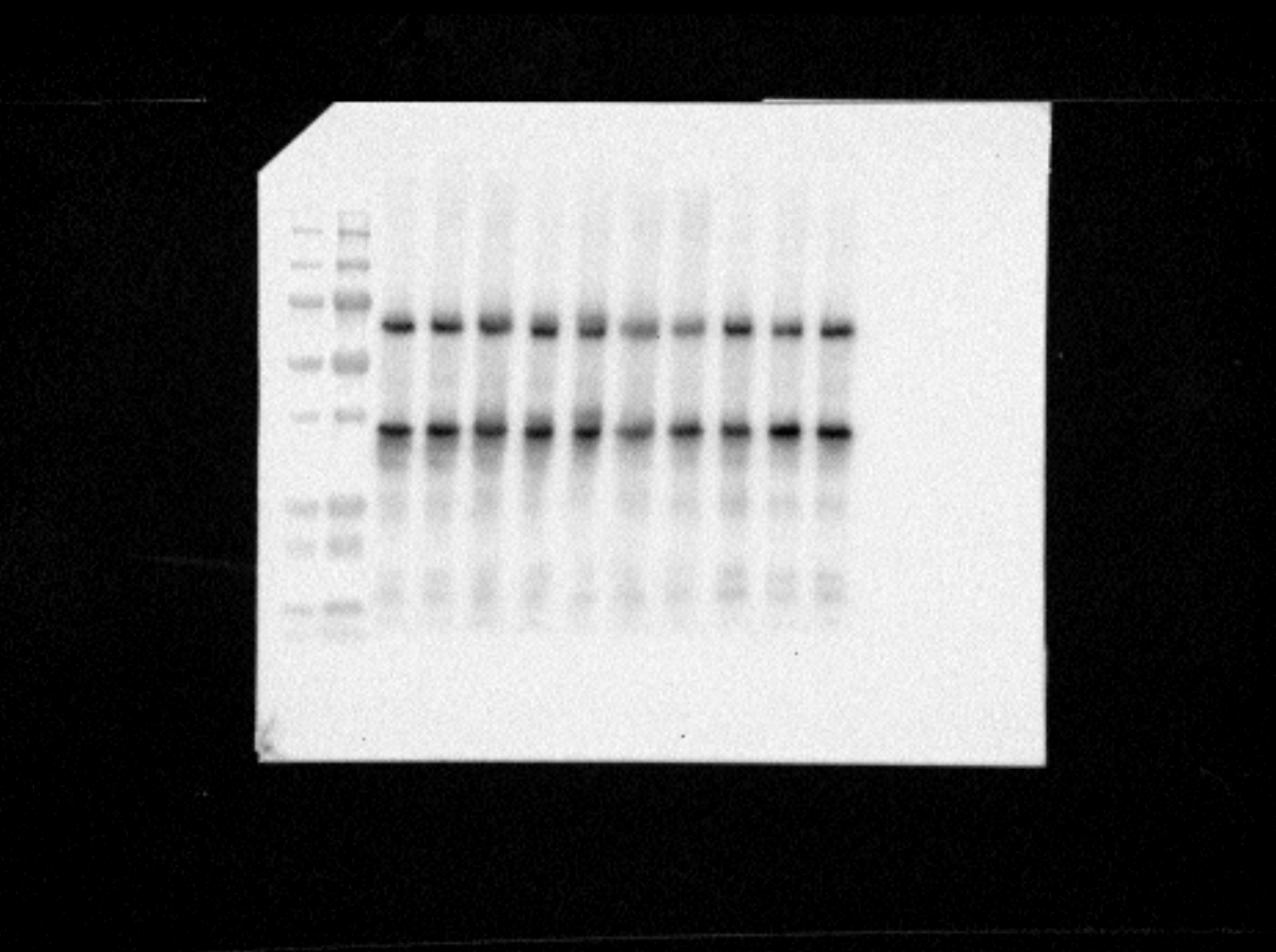

Supplement: Figure 4—figure supplement 2—source data 1. [file elife-89136-fig4-figsupp2-data1.zip › Figure 4-figure supplement 2-Source Data/Figure 4-figure supplement 2-Source Data-2 (raw WB images)/Figure 4-figure supplement 2E-(AMPK)-GAPDH.jpg]

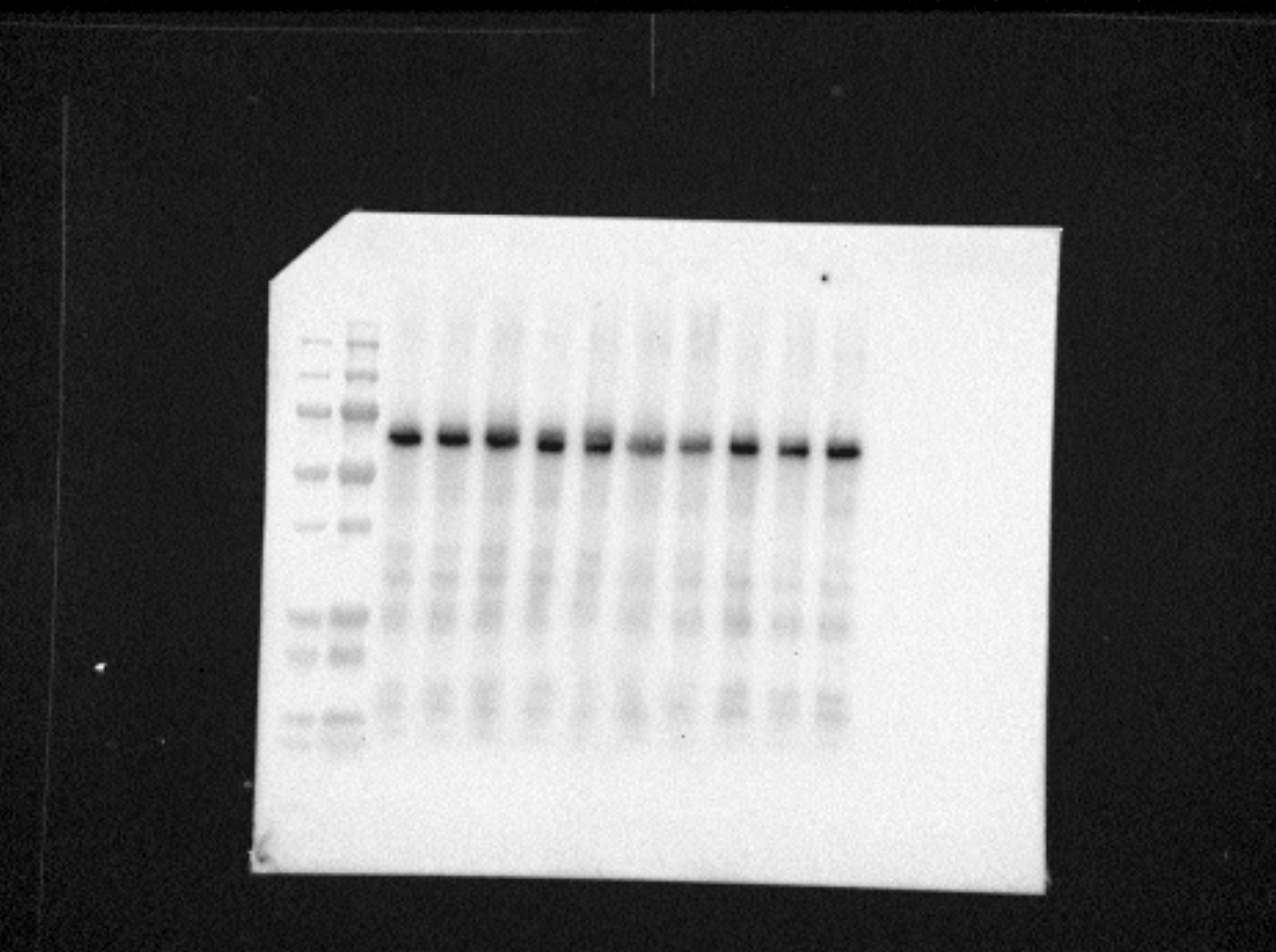

Supplement: Figure 4—figure supplement 2—source data 1. [file elife-89136-fig4-figsupp2-data1.zip › Figure 4-figure supplement 2-Source Data/Figure 4-figure supplement 2-Source Data-2 (raw WB images)/Figure 4-figure supplement 2E-AMPK.jpg]

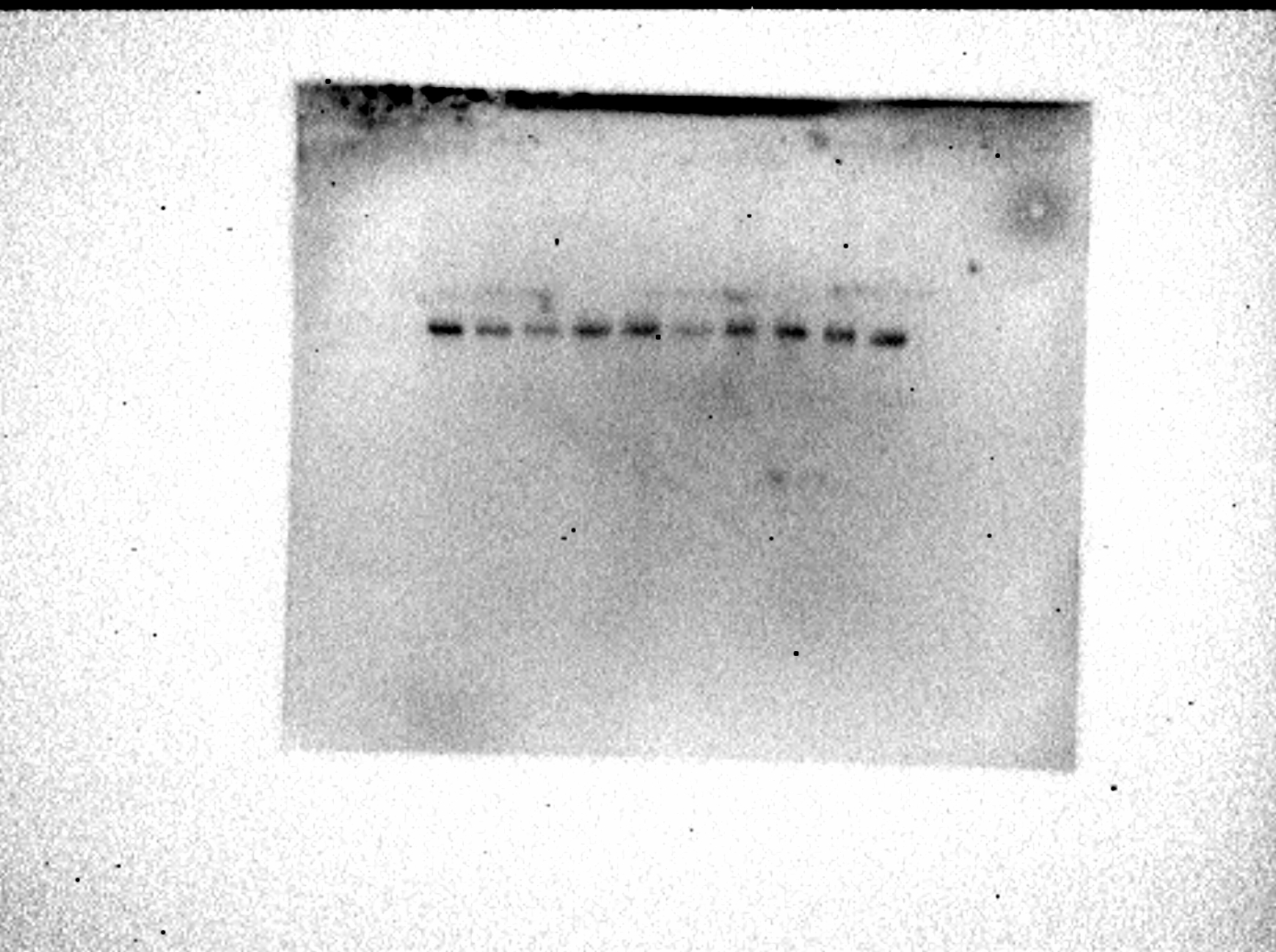

Supplement: Figure 4—figure supplement 2—source data 1. [file elife-89136-fig4-figsupp2-data1.zip › Figure 4-figure supplement 2-Source Data/Figure 4-figure supplement 2-Source Data-2 (raw WB images)/Figure 4-figure supplement 2E-pAMPK.jpg]

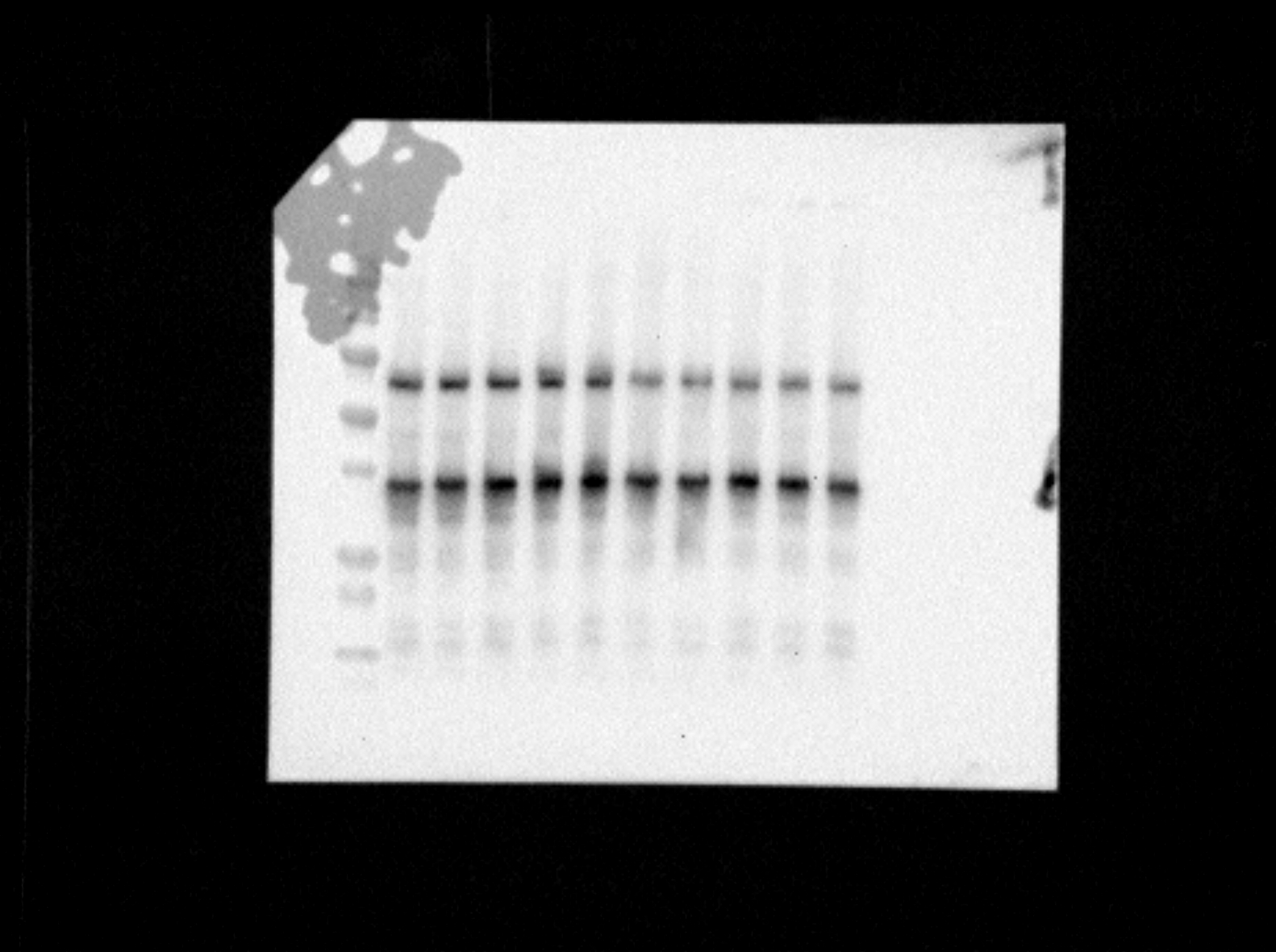

Supplement: Figure 4—figure supplement 2—source data 1. [file elife-89136-fig4-figsupp2-data1.zip › Figure 4-figure supplement 2-Source Data/Figure 4-figure supplement 2-Source Data-2 (raw WB images)/Figure 4-figure supplement 2F-AMPK-GAPDH.jpg]

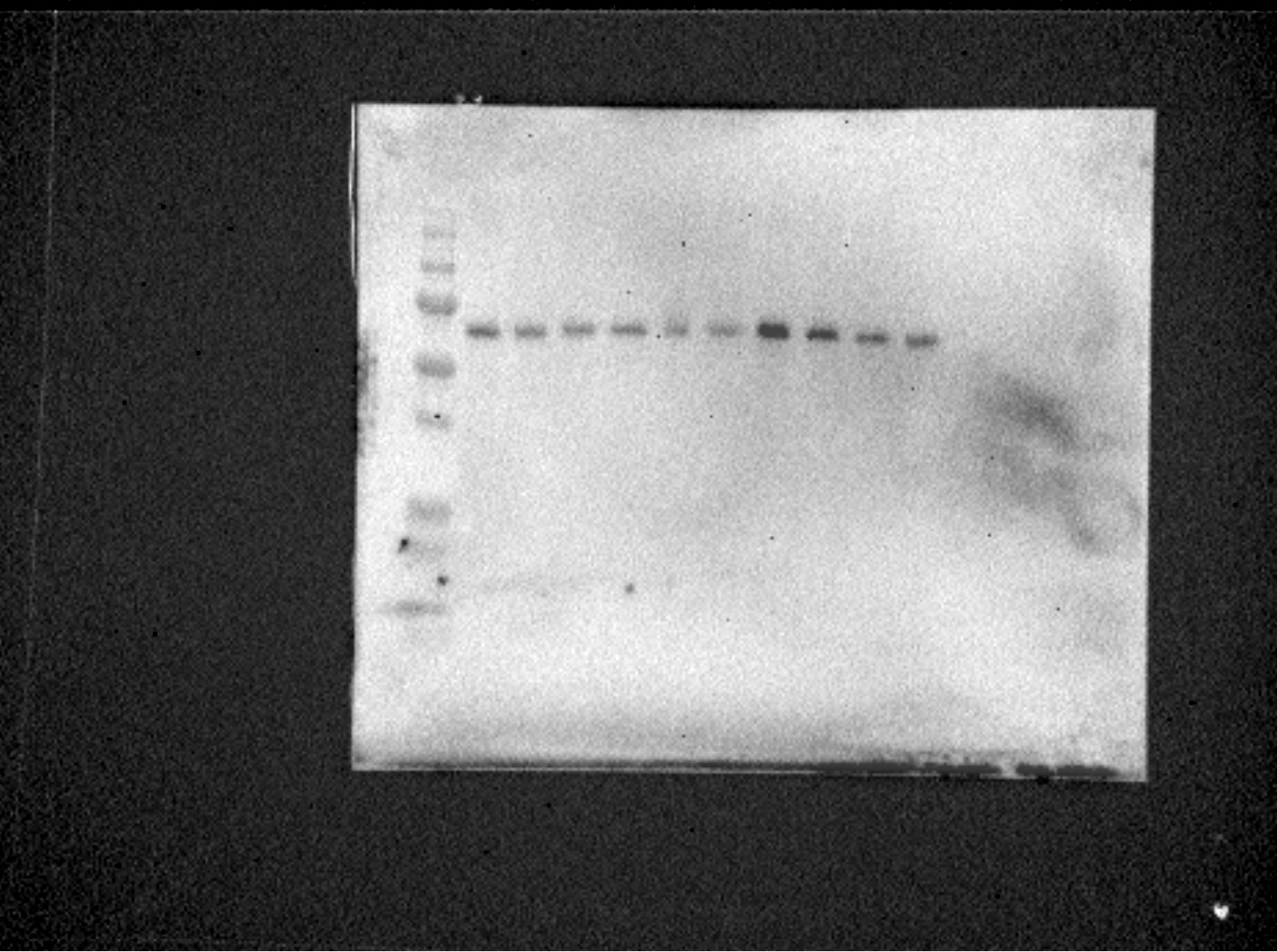

Supplement: Figure 4—figure supplement 2—source data 1. [file elife-89136-fig4-figsupp2-data1.zip › Figure 4-figure supplement 2-Source Data/Figure 4-figure supplement 2-Source Data-2 (raw WB images)/Figure 4-figure supplement 2F-pAMPK.jpg]

## Slide 1
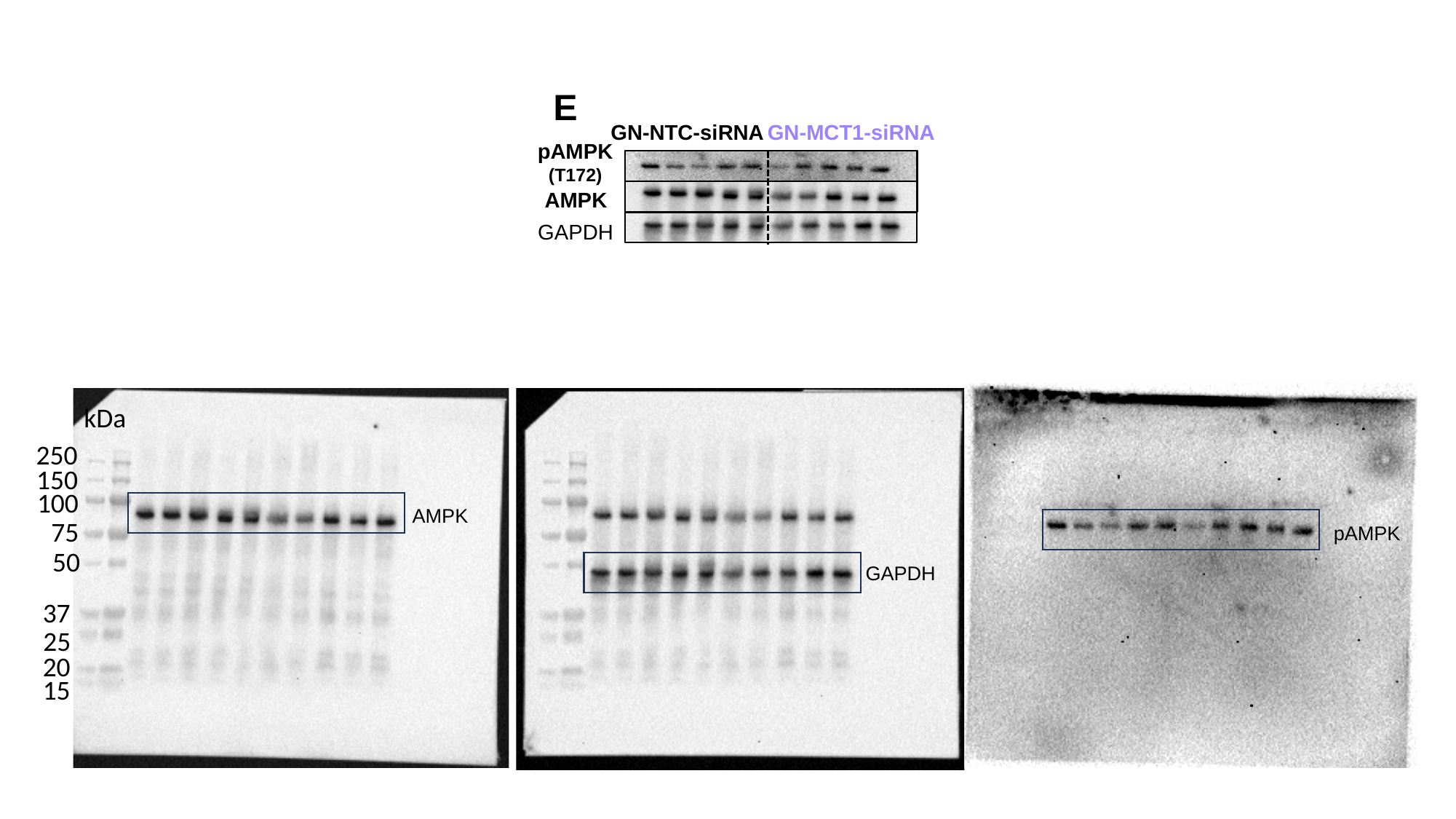

E
GN-NTC-siRNA
GN-MCT1-siRNA
pAMPK
(T172)
AMPK
GAPDH
kDa
250
150
100
75
50
37
25
20
15
AMPK
pAMPK
GAPDH

Supplement: Figure 4—figure supplement 2—source data 1. [file elife-89136-fig4-figsupp2-data1.zip › Figure 4-figure supplement 2-Source Data/Figure 4-figure supplement 2-Source Data-3 (labeled WB images)/Figure 4-figure supplement 2E.pptx]

## Slide 1
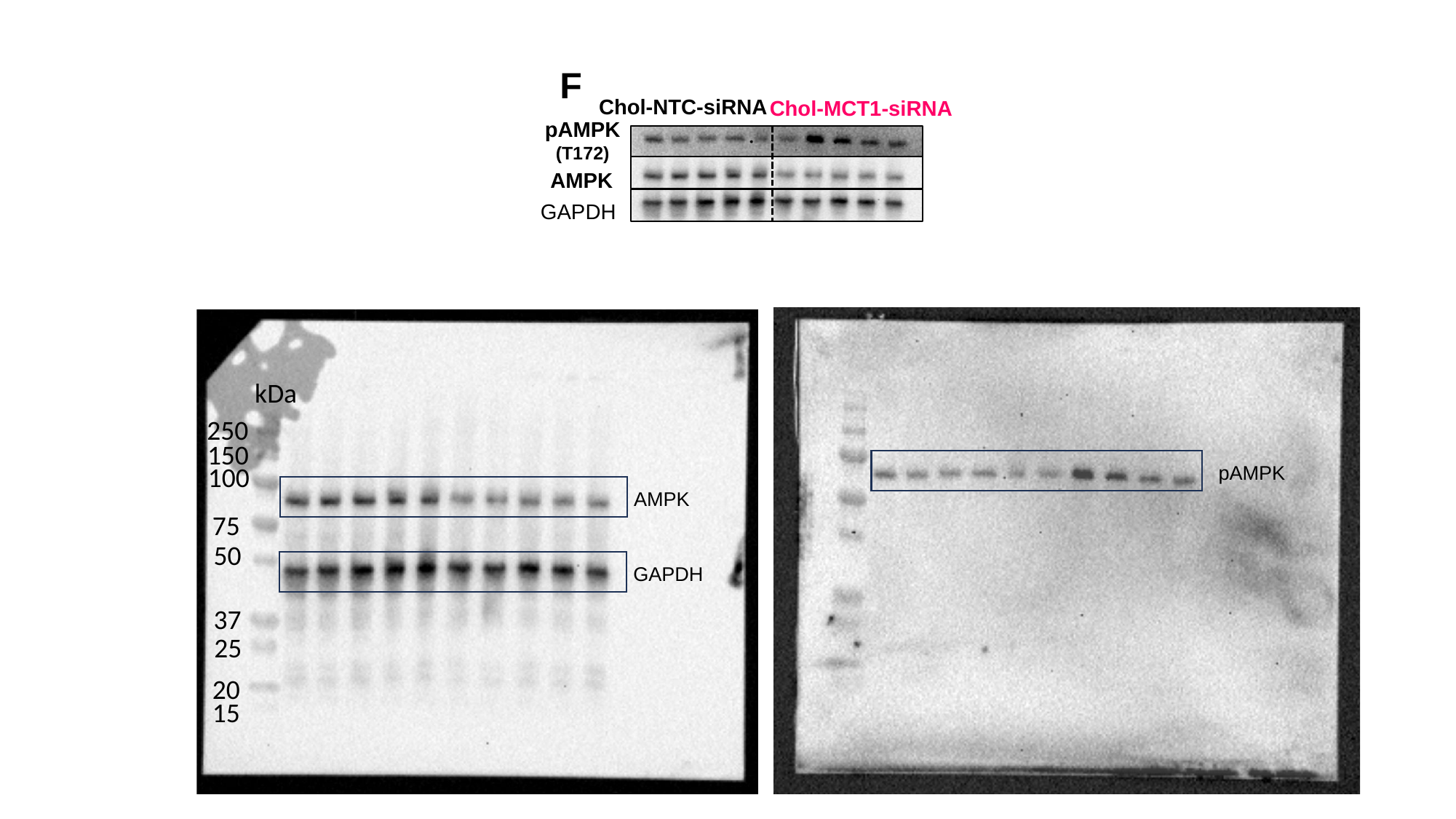

F
Chol-NTC-siRNA
Chol-MCT1-siRNA
pAMPK
(T172)
AMPK
GAPDH
kDa
250
150
100
75
50
37
25
20
15
pAMPK
AMPK
GAPDH

Supplement: Figure 4—figure supplement 2—source data 1. [file elife-89136-fig4-figsupp2-data1.zip › Figure 4-figure supplement 2-Source Data/Figure 4-figure supplement 2-Source Data-3 (labeled WB images)/Figure 4-figure supplement 2F.pptx]

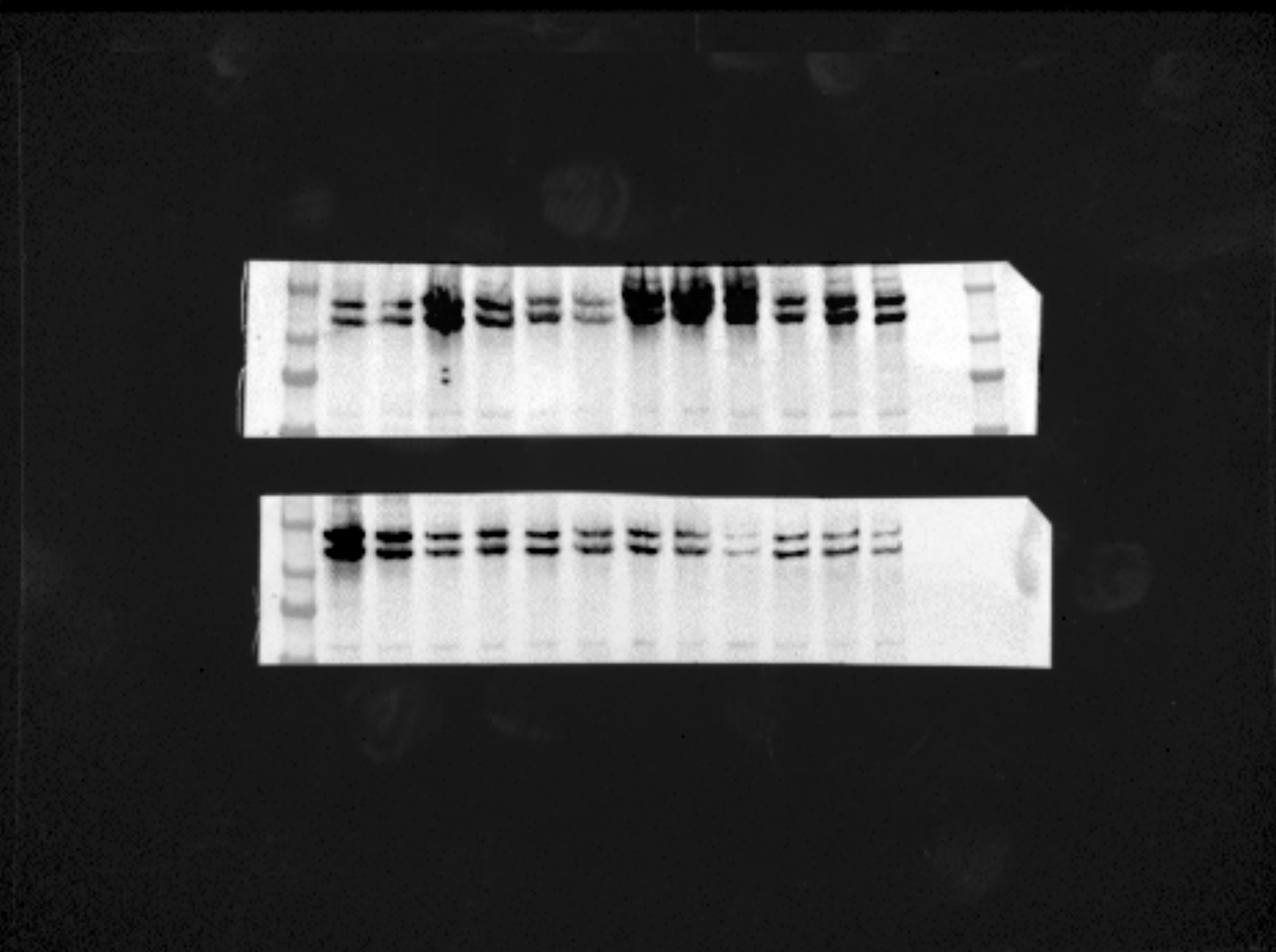

Supplement: Figure 5—source data 1. [file elife-89136-fig5-data1.zip › Figure 5-Source Data/Figure 5-Source Data-1 (raw WB images)/Figure 5A&5D-Col1.jpg]

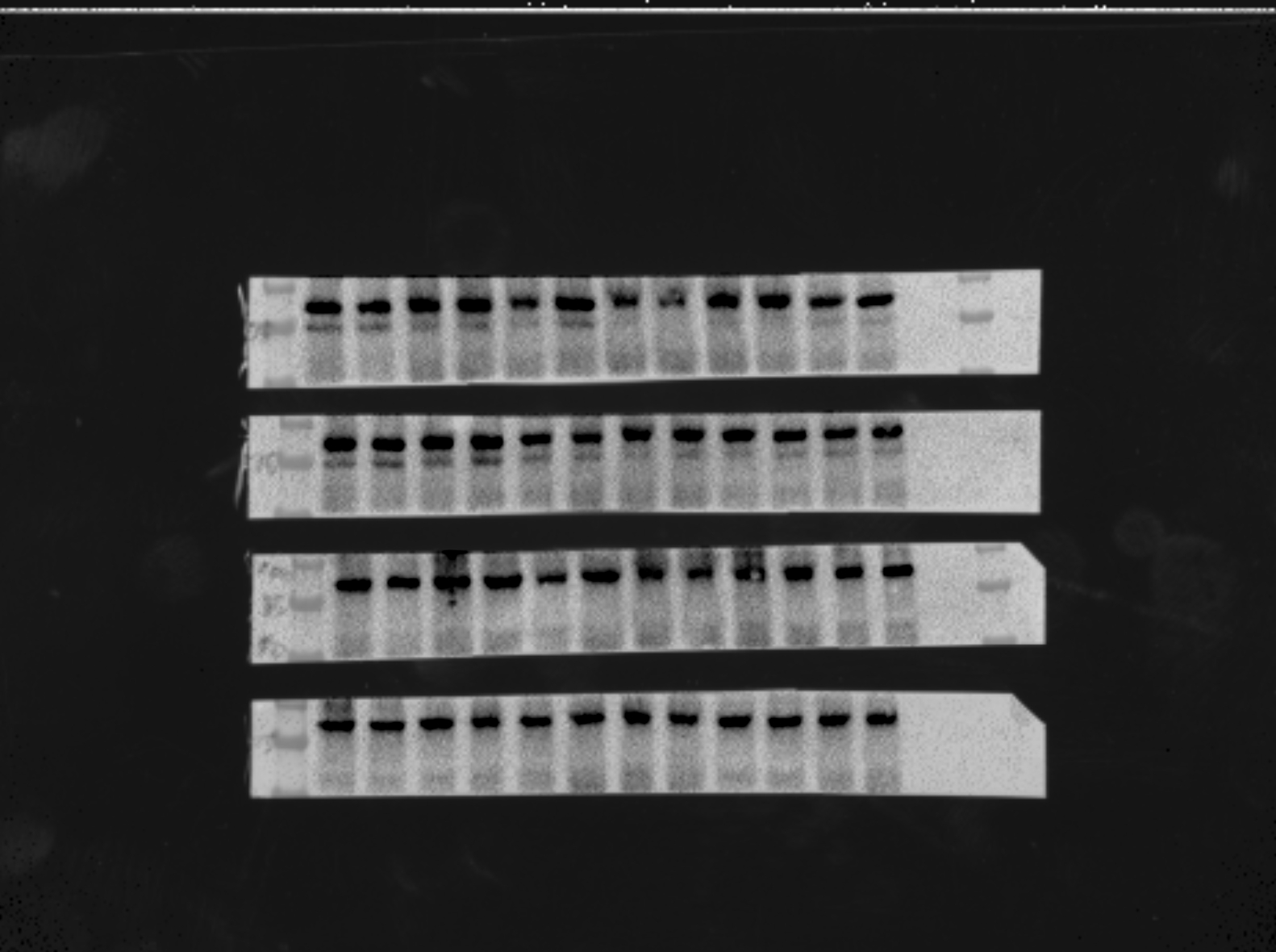

Supplement: Figure 5—source data 1. [file elife-89136-fig5-data1.zip › Figure 5-Source Data/Figure 5-Source Data-1 (raw WB images)/Figure 5A&5D-HSP90.jpg]

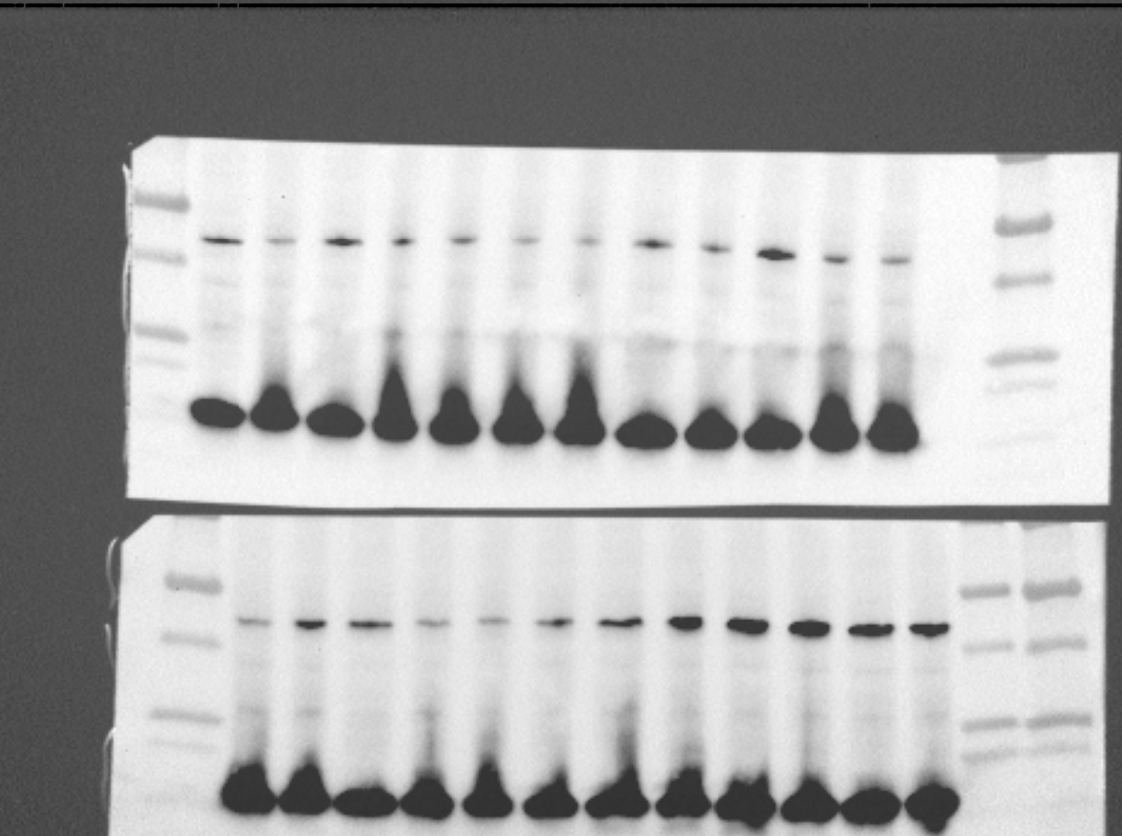

Supplement: Figure 5—source data 1. [file elife-89136-fig5-data1.zip › Figure 5-Source Data/Figure 5-Source Data-1 (raw WB images)/Figure 5A&D-aSMA-H3.jpg]

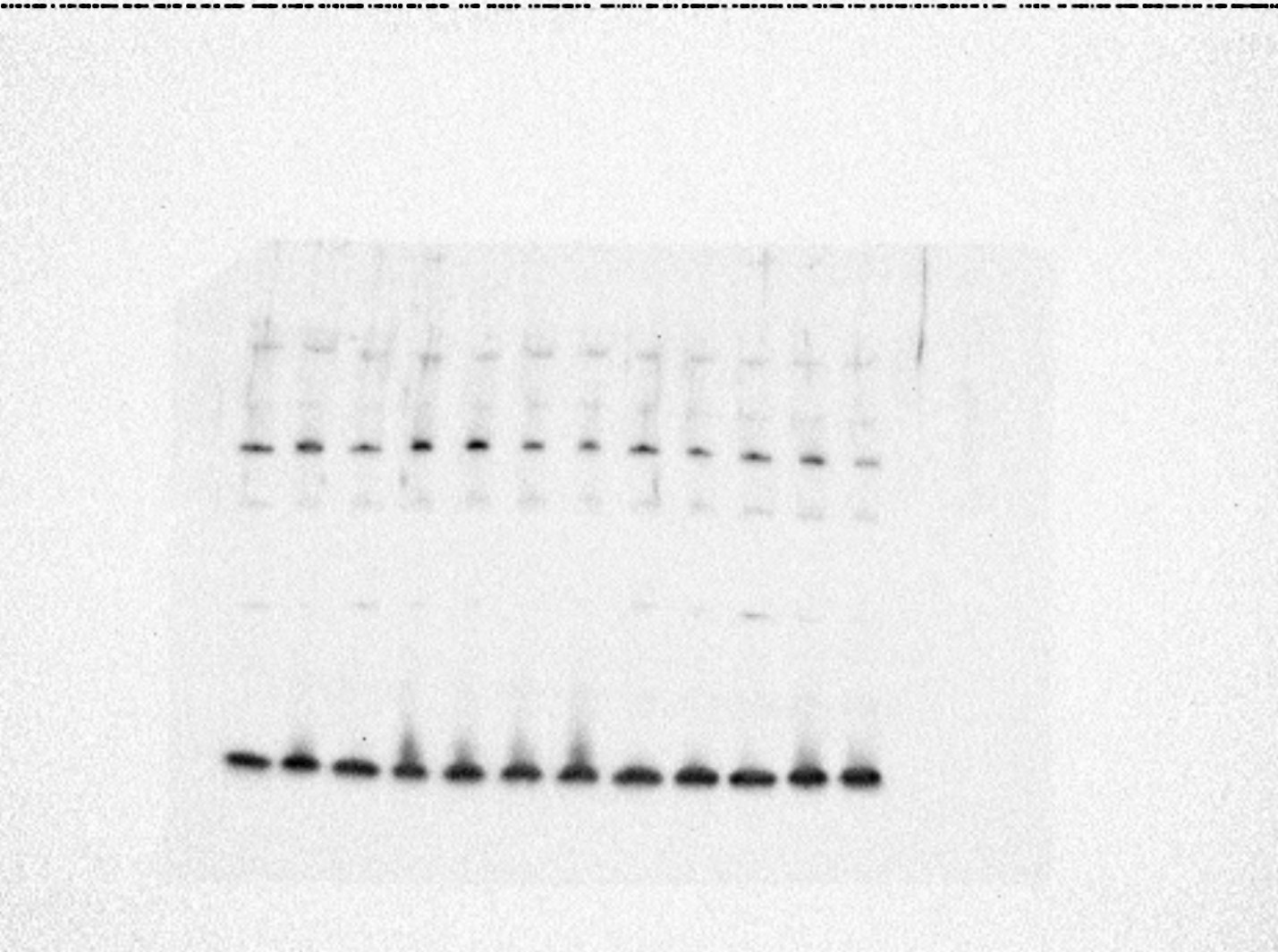

Supplement: Figure 5—source data 1. [file elife-89136-fig5-data1.zip › Figure 5-Source Data/Figure 5-Source Data-1 (raw WB images)/Figure 5A-H3.jpg]

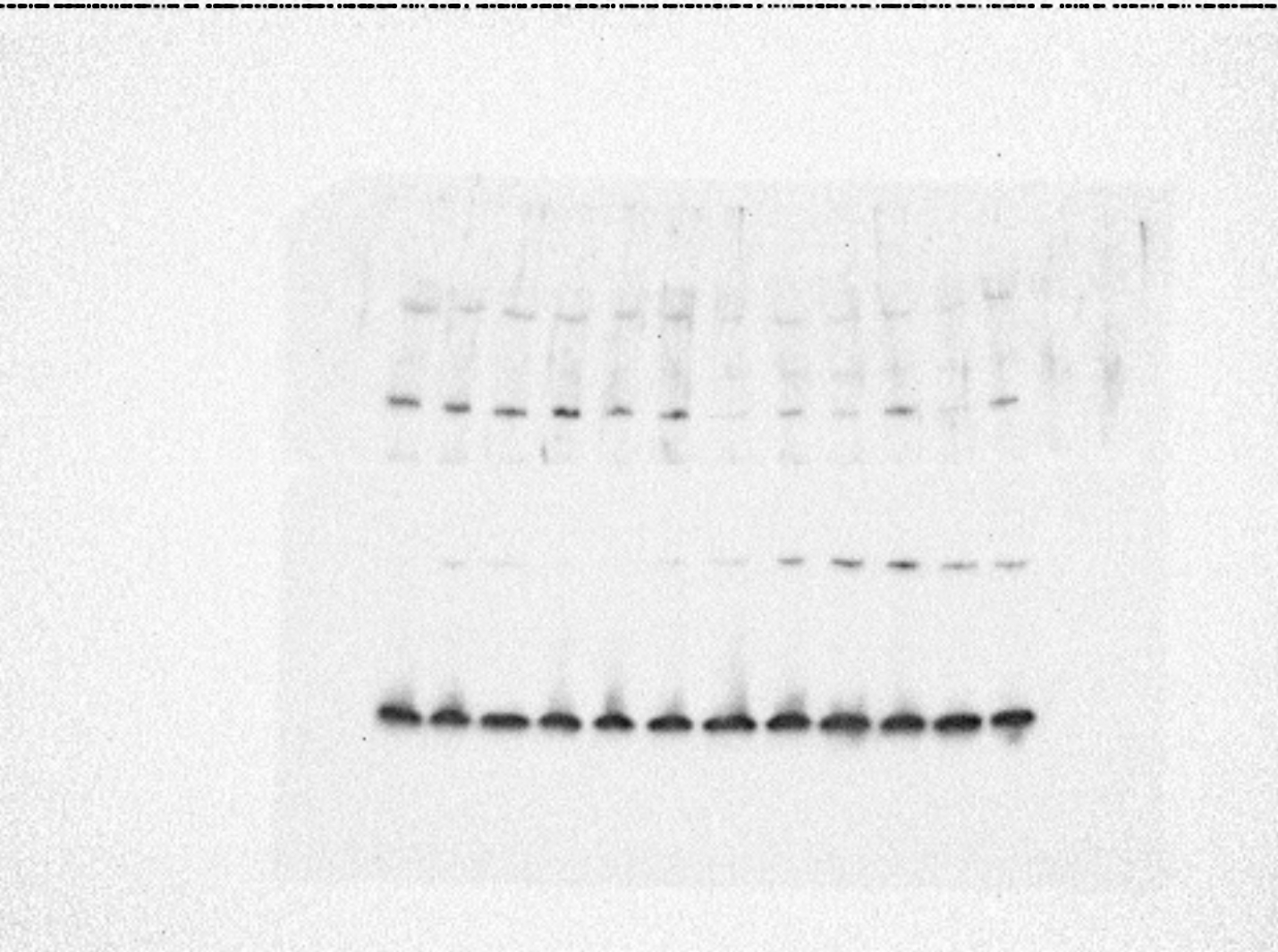

Supplement: Figure 5—source data 1. [file elife-89136-fig5-data1.zip › Figure 5-Source Data/Figure 5-Source Data-1 (raw WB images)/Figure 5D-H3.jpg]

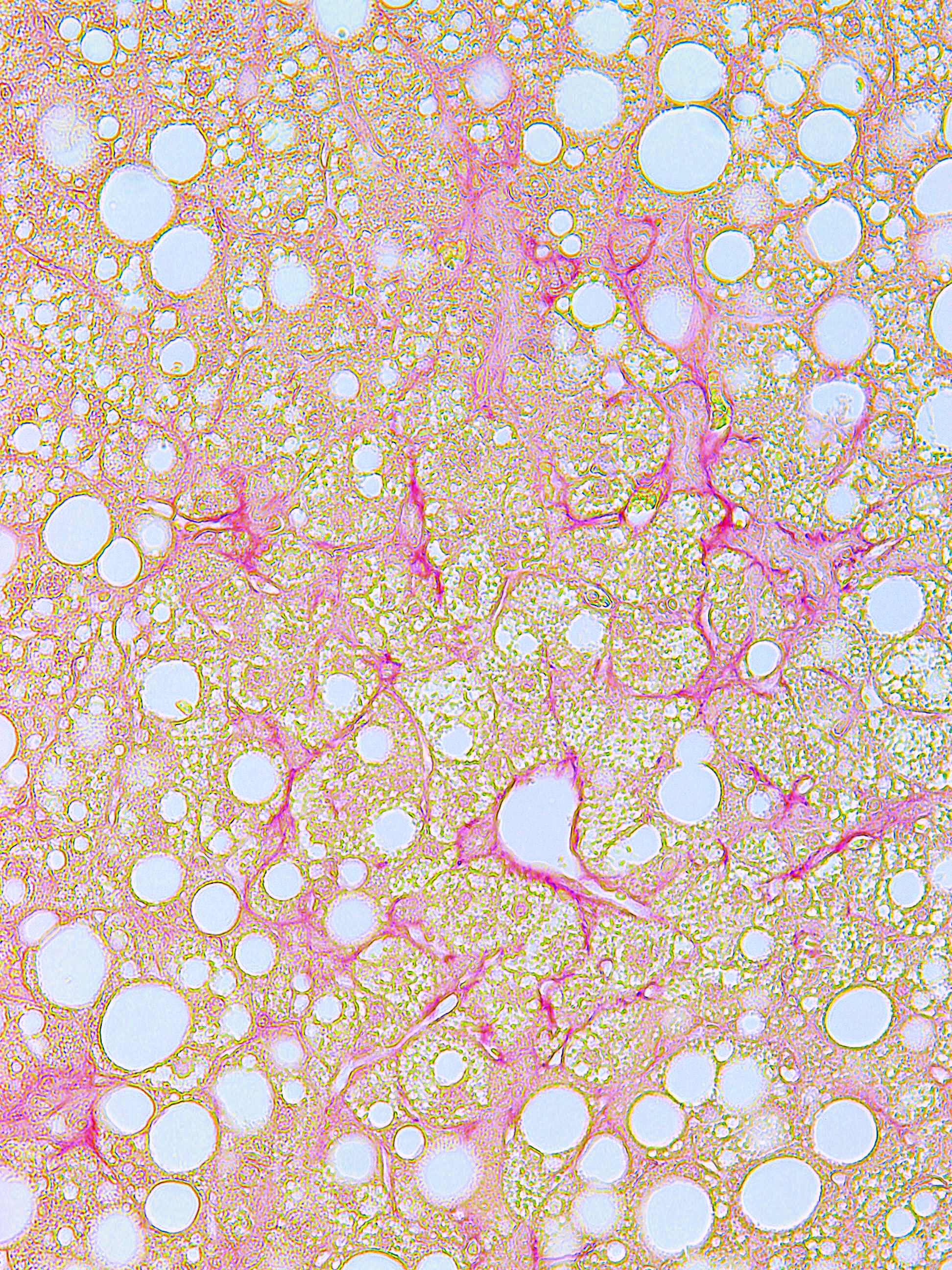

Supplement: Figure 5—source data 1. [file elife-89136-fig5-data1.zip › Figure 5-Source Data/Figure 5-Source Data-4 (raw IHC images)/Figure 5G-Chol-MCT1-siRNA.jpg]

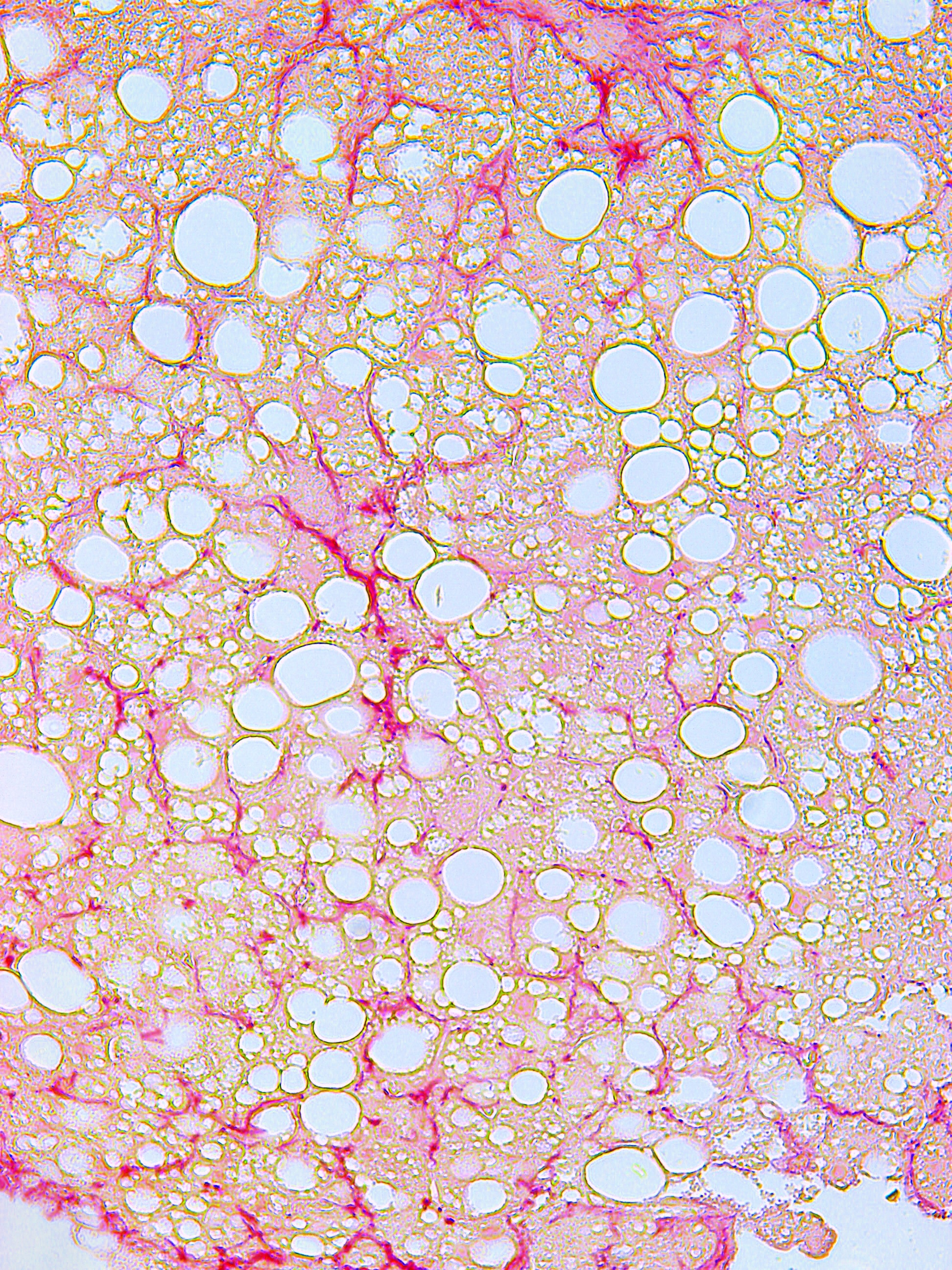

Supplement: Figure 5—source data 1. [file elife-89136-fig5-data1.zip › Figure 5-Source Data/Figure 5-Source Data-4 (raw IHC images)/Figure 5G-Chol-NTC-siRNA.jpg]

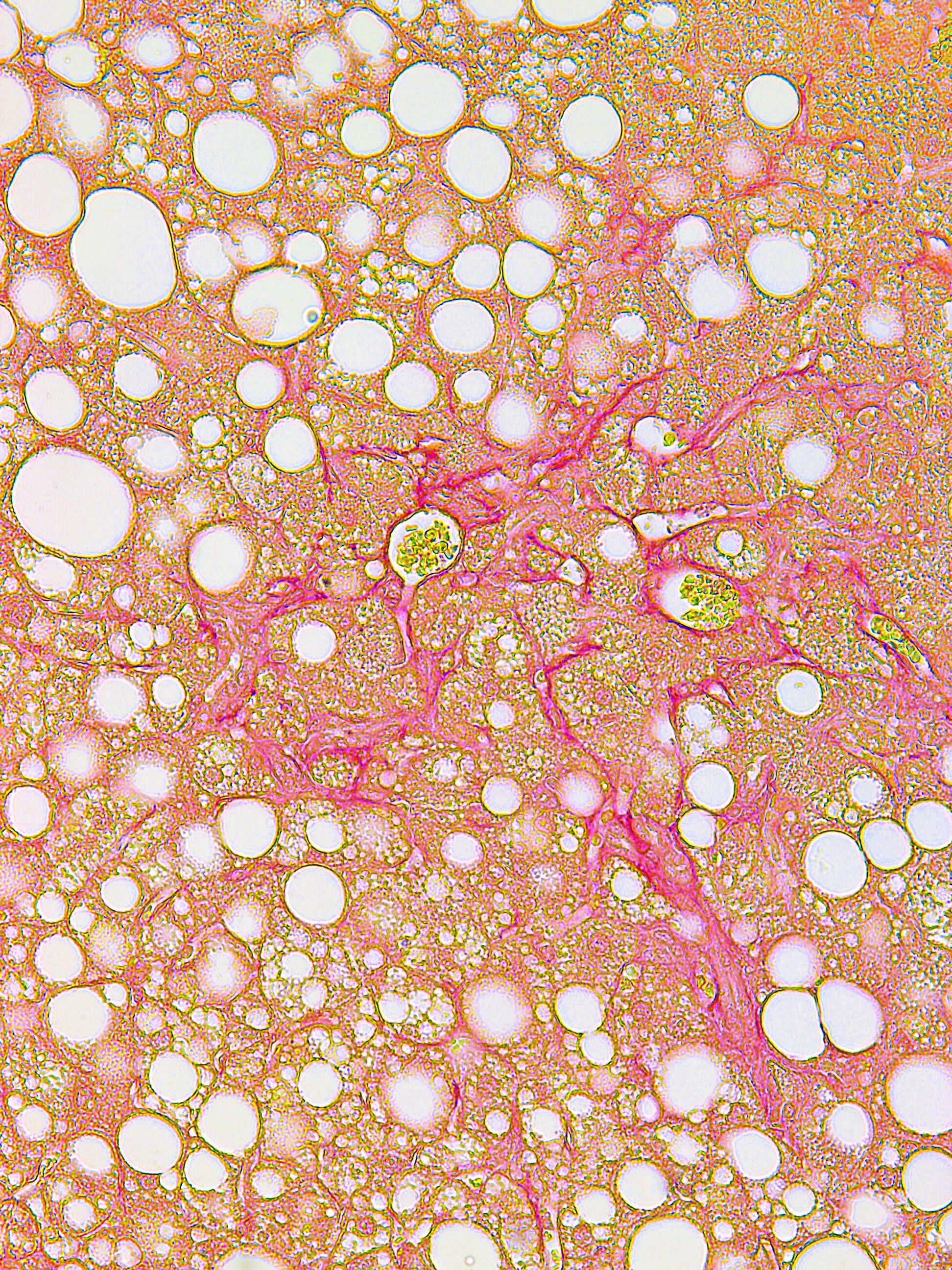

Supplement: Figure 5—source data 1. [file elife-89136-fig5-data1.zip › Figure 5-Source Data/Figure 5-Source Data-4 (raw IHC images)/Figure 5G-GN-MCT1-siRNA.jpg]

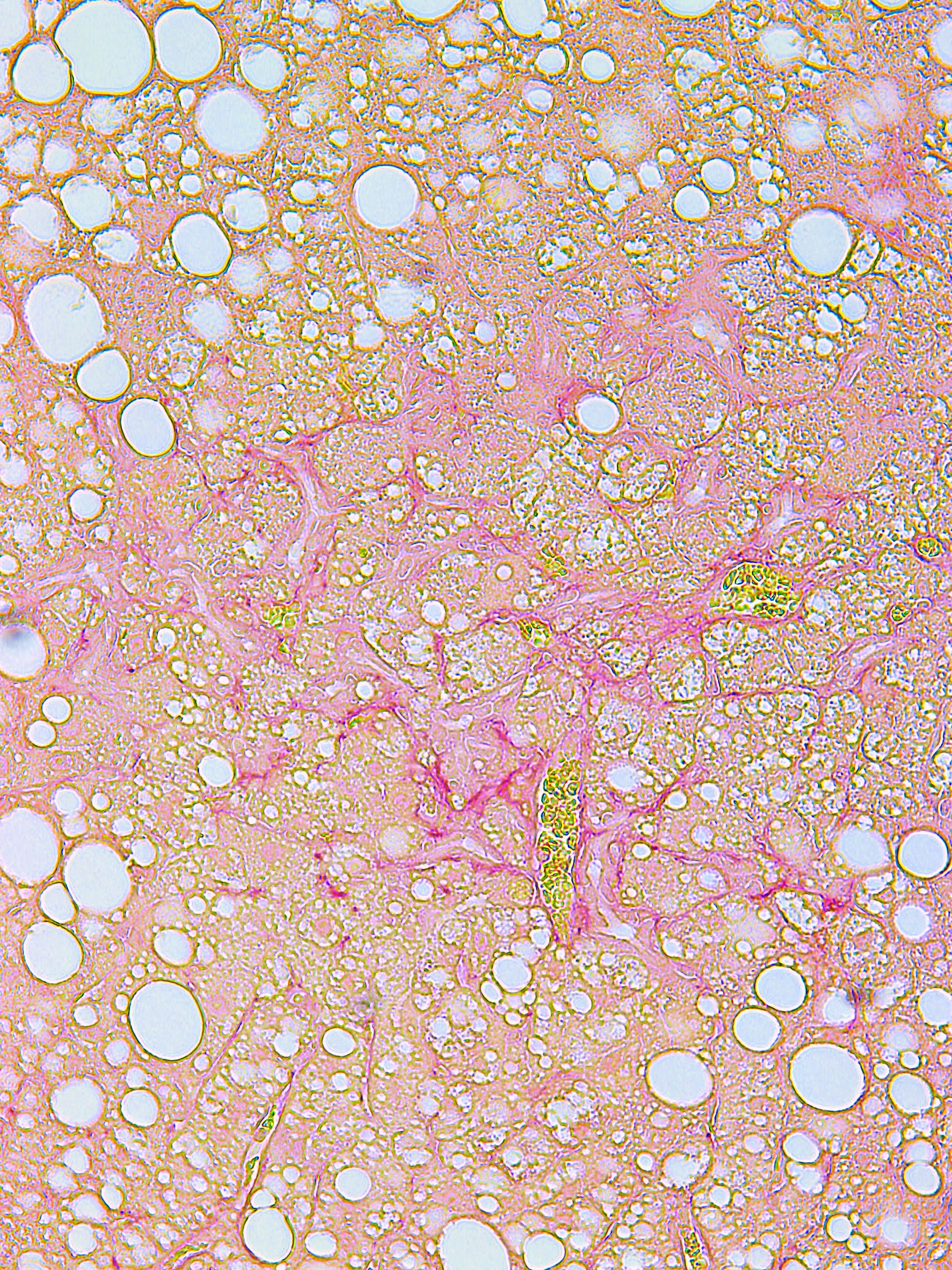

Supplement: Figure 5—source data 1. [file elife-89136-fig5-data1.zip › Figure 5-Source Data/Figure 5-Source Data-4 (raw IHC images)/Figure 5G-GN-NTC-siRNA.jpg]

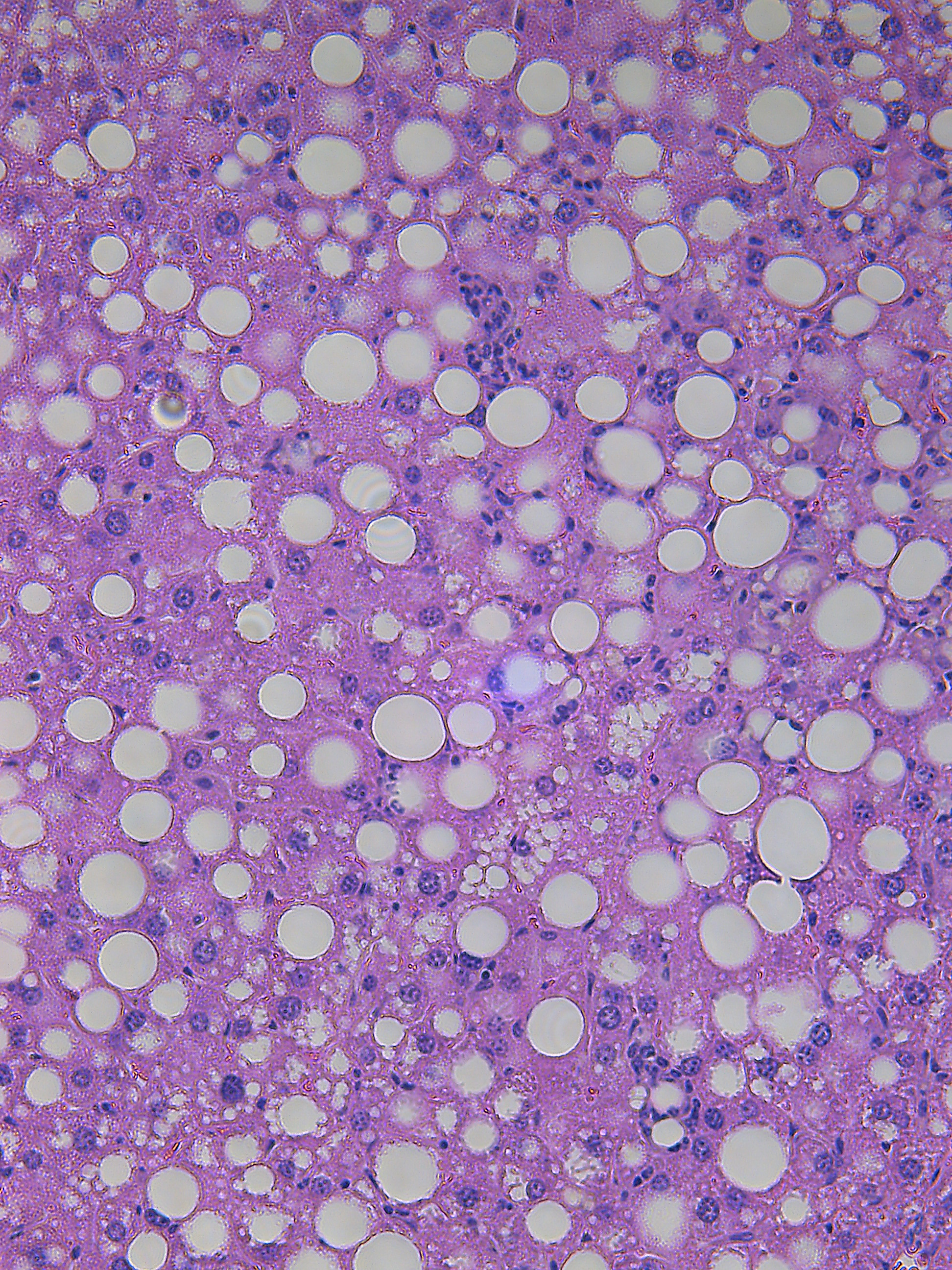

Supplement: Figure 6—source data 1. [file elife-89136-fig6-data1.zip › Figure 6-Source Data/Figure 6-Source Data-2 (raw IHC images)/Figure 6E-Control.tif]

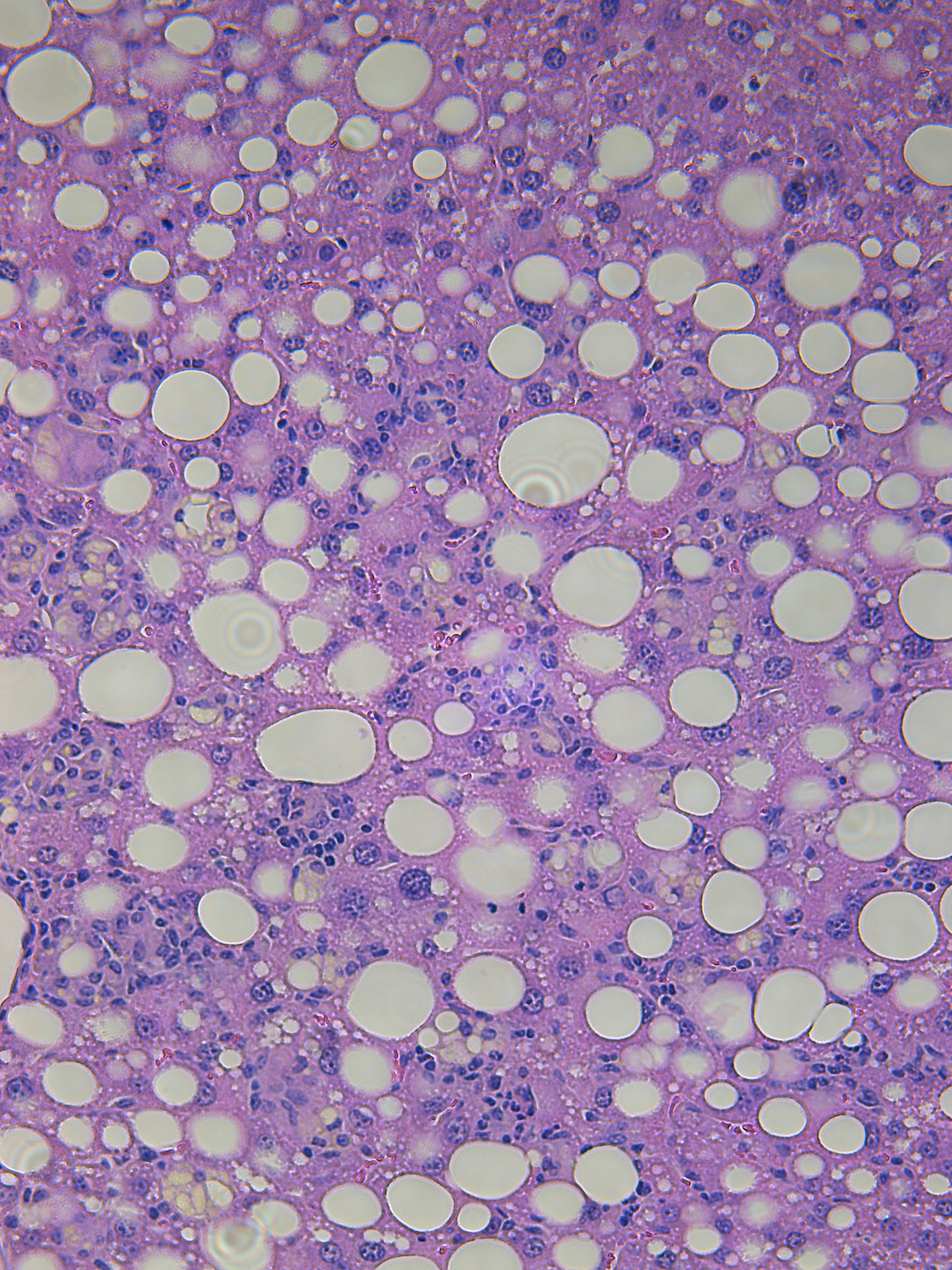

Supplement: Figure 6—source data 1. [file elife-89136-fig6-data1.zip › Figure 6-Source Data/Figure 6-Source Data-2 (raw IHC images)/Figure 6E-Hep KO & HSC KO.tif]

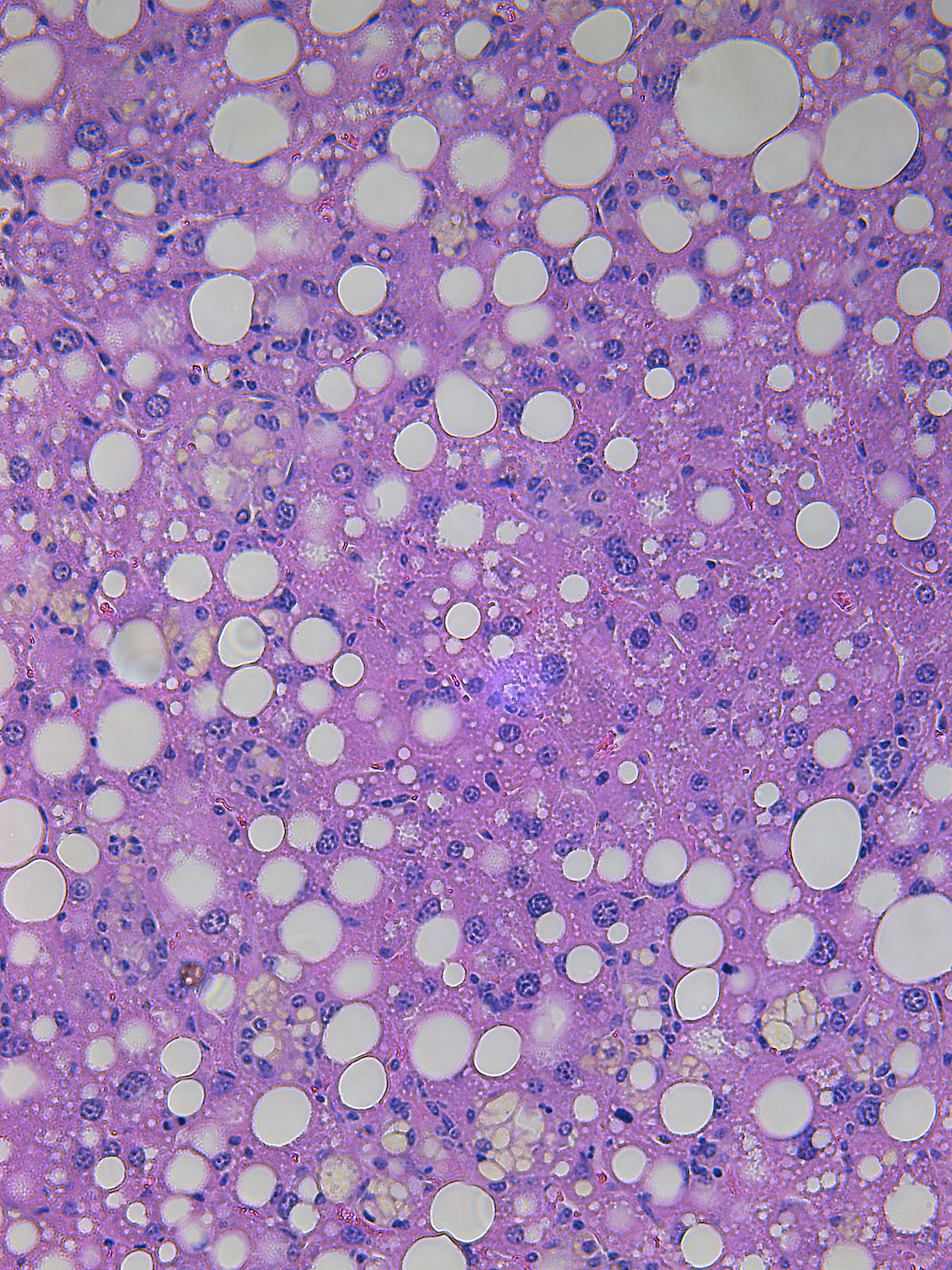

Supplement: Figure 6—source data 1. [file elife-89136-fig6-data1.zip › Figure 6-Source Data/Figure 6-Source Data-2 (raw IHC images)/Figure 6E-Hep KO.tif]

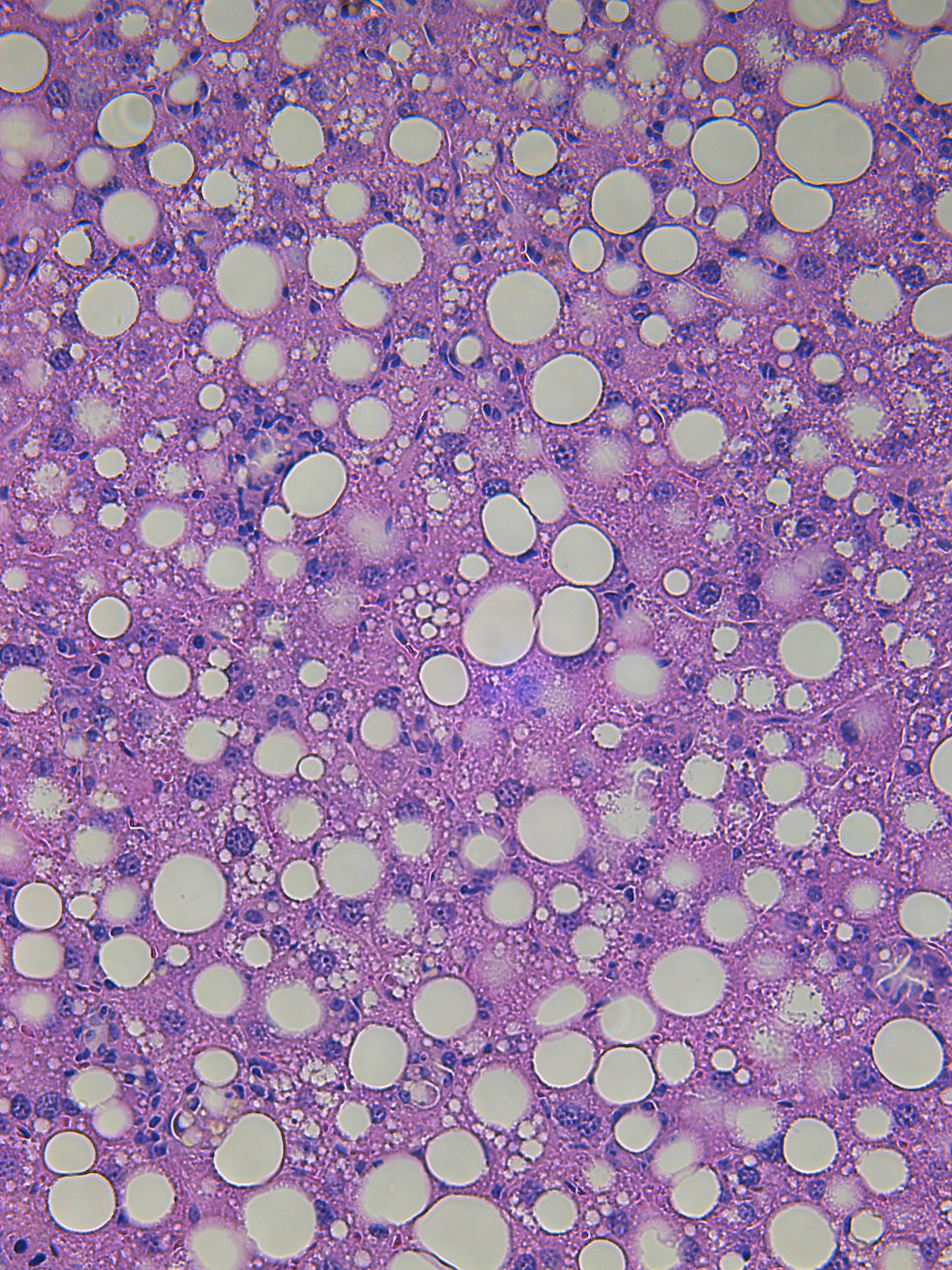

Supplement: Figure 6—source data 1. [file elife-89136-fig6-data1.zip › Figure 6-Source Data/Figure 6-Source Data-2 (raw IHC images)/Figure 6E-HSC KO.tif]

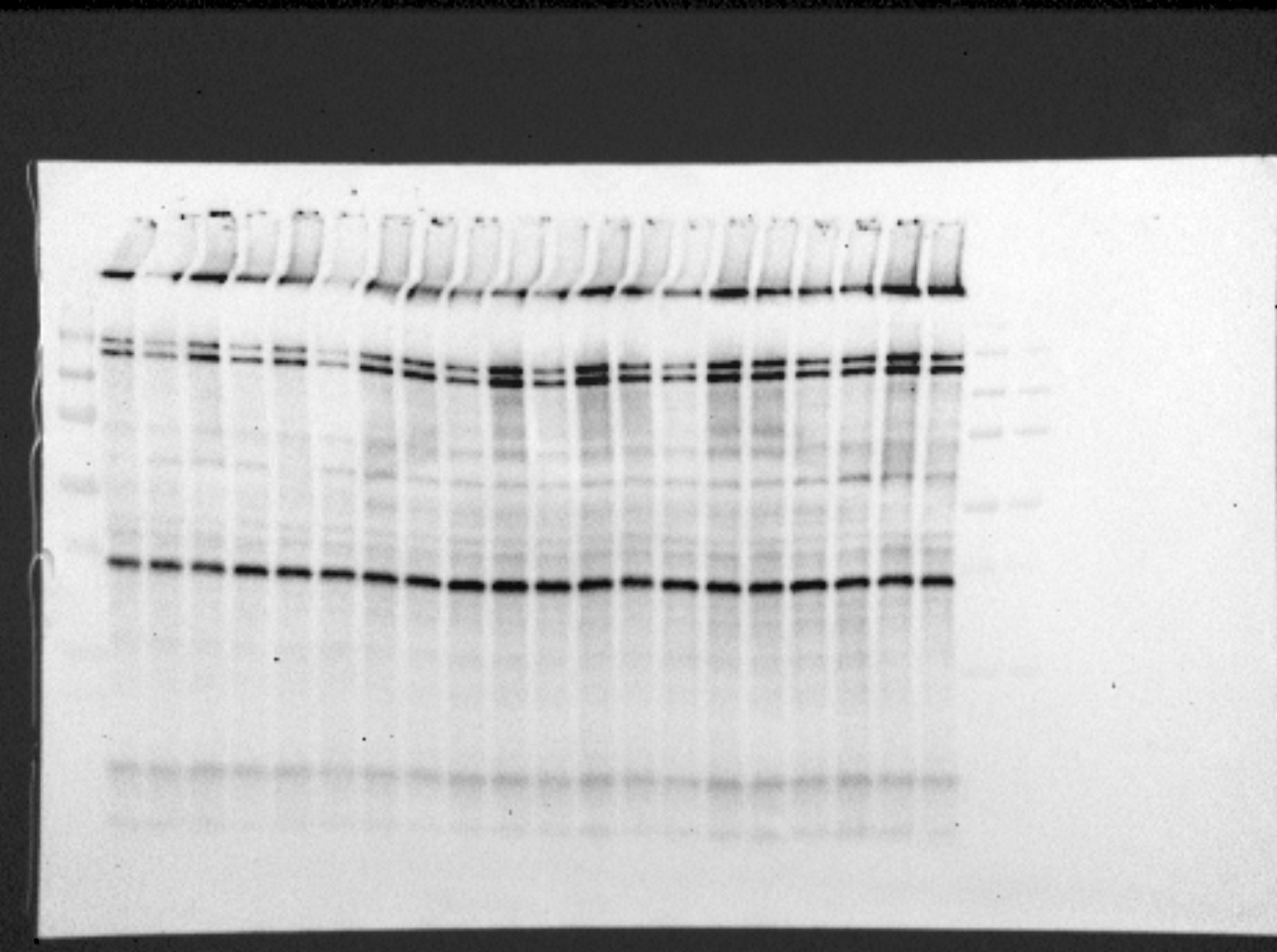

Supplement: Figure 7—source data 1. [file elife-89136-fig7-data1.zip › Figure 7-Source Data/Figure 7-Source Data-1 (raw WB images)/Figure 7A-Col1.jpg]

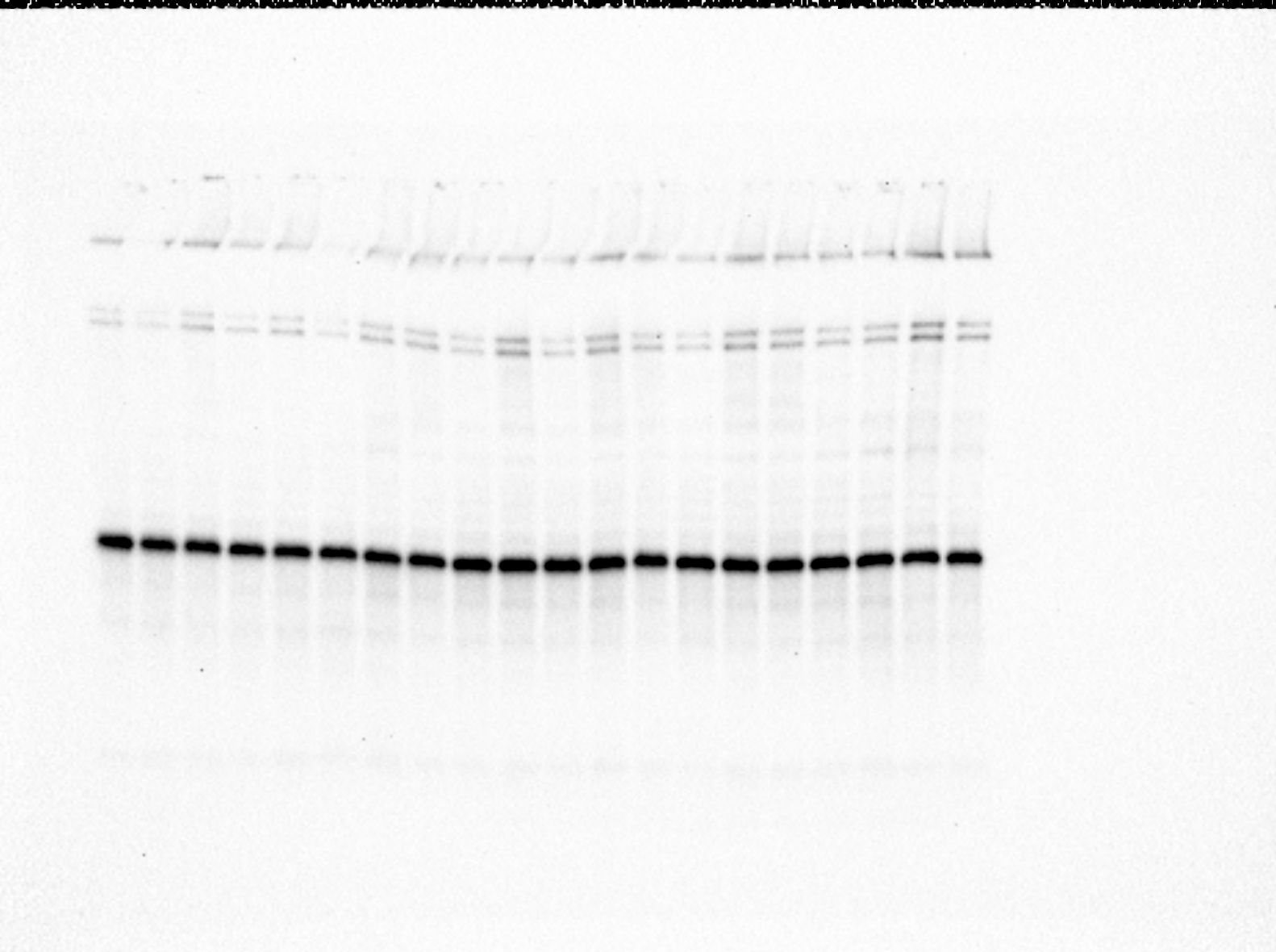

Supplement: Figure 7—source data 1. [file elife-89136-fig7-data1.zip › Figure 7-Source Data/Figure 7-Source Data-1 (raw WB images)/Figure 7A-GAPDH.jpg]

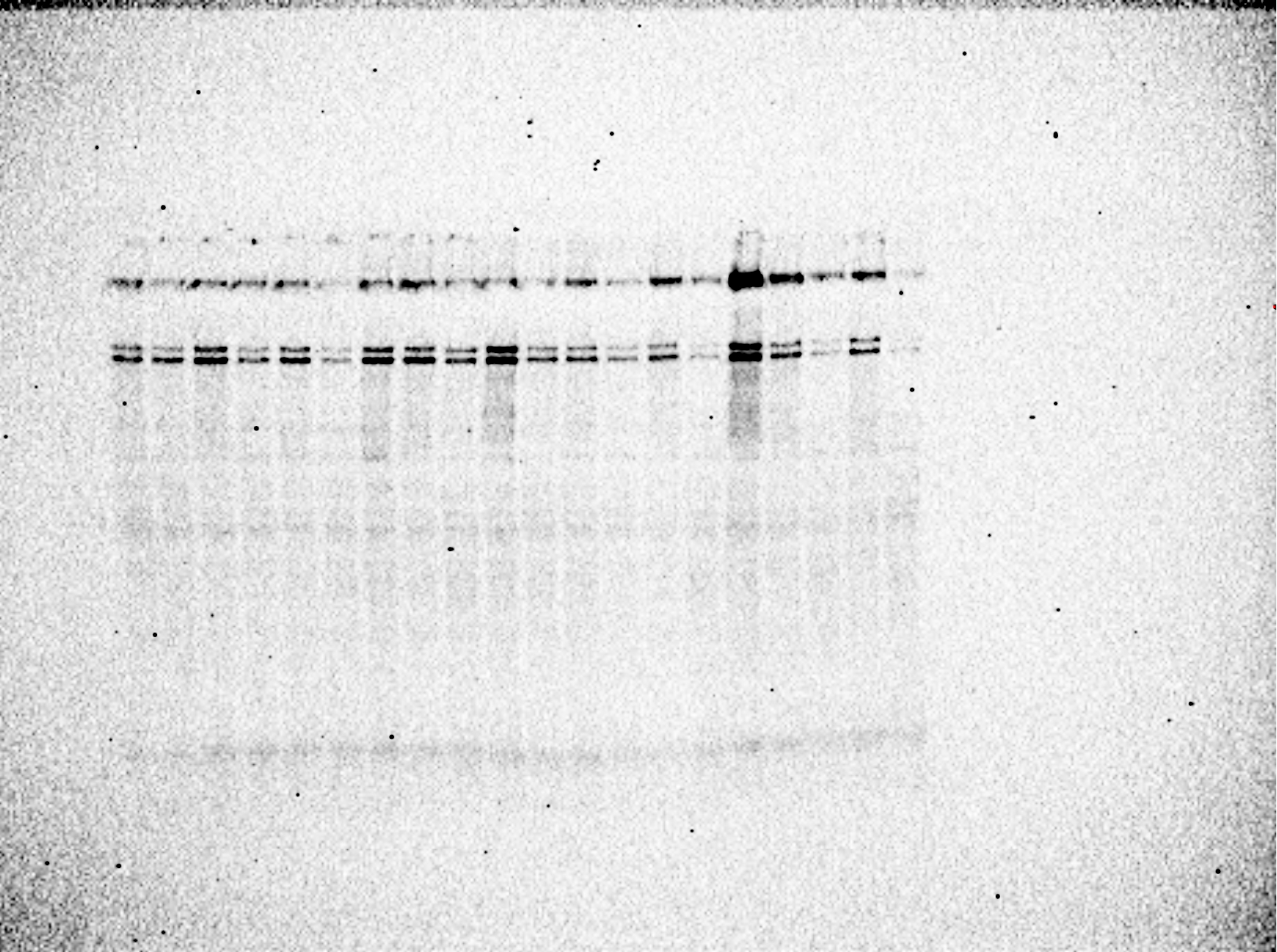

Supplement: Figure 7—source data 1. [file elife-89136-fig7-data1.zip › Figure 7-Source Data/Figure 7-Source Data-1 (raw WB images)/Figure 7B-Col1.jpg]

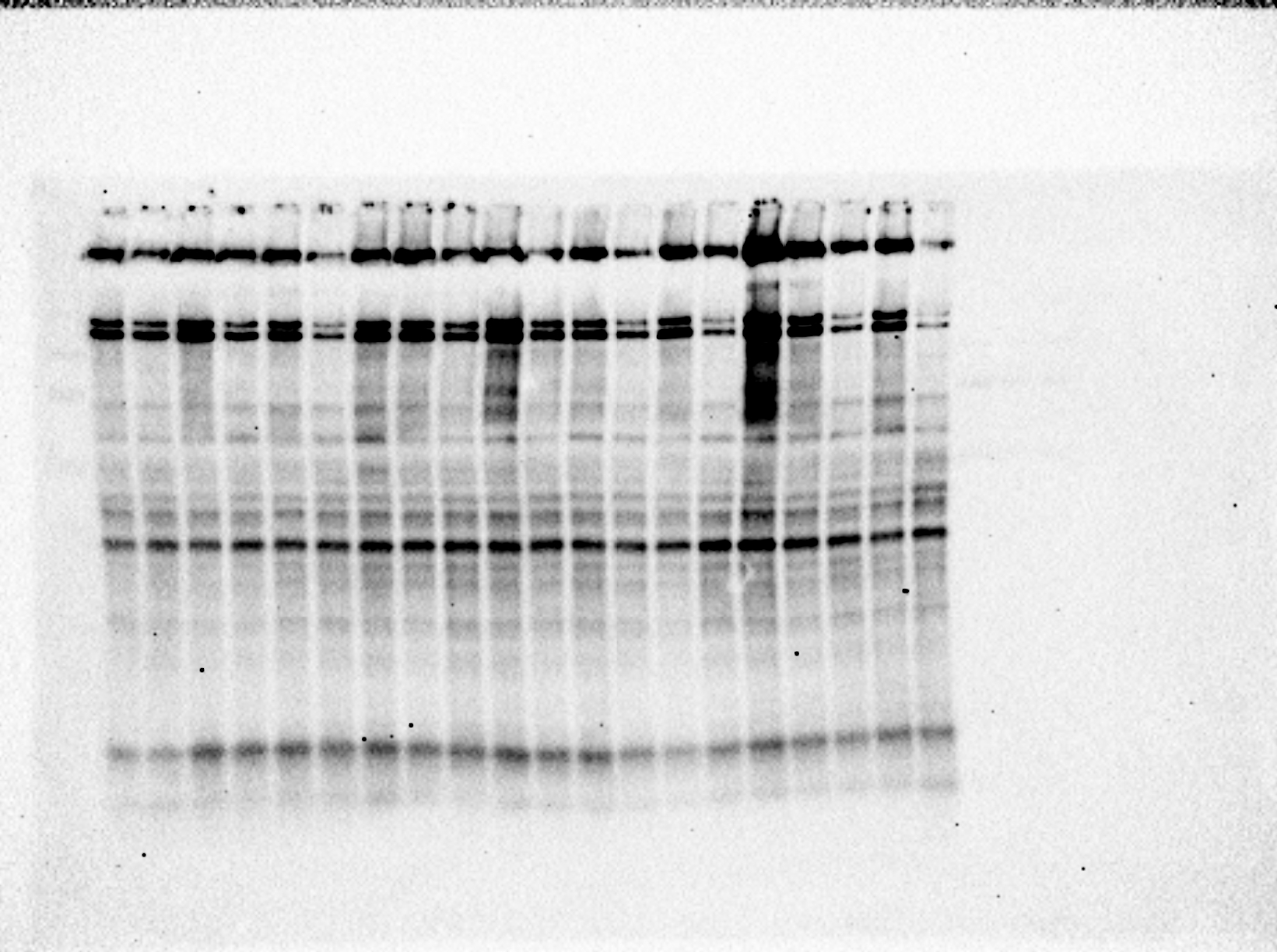

Supplement: Figure 7—source data 1. [file elife-89136-fig7-data1.zip › Figure 7-Source Data/Figure 7-Source Data-1 (raw WB images)/Figure 7B-GAPDH.jpg]

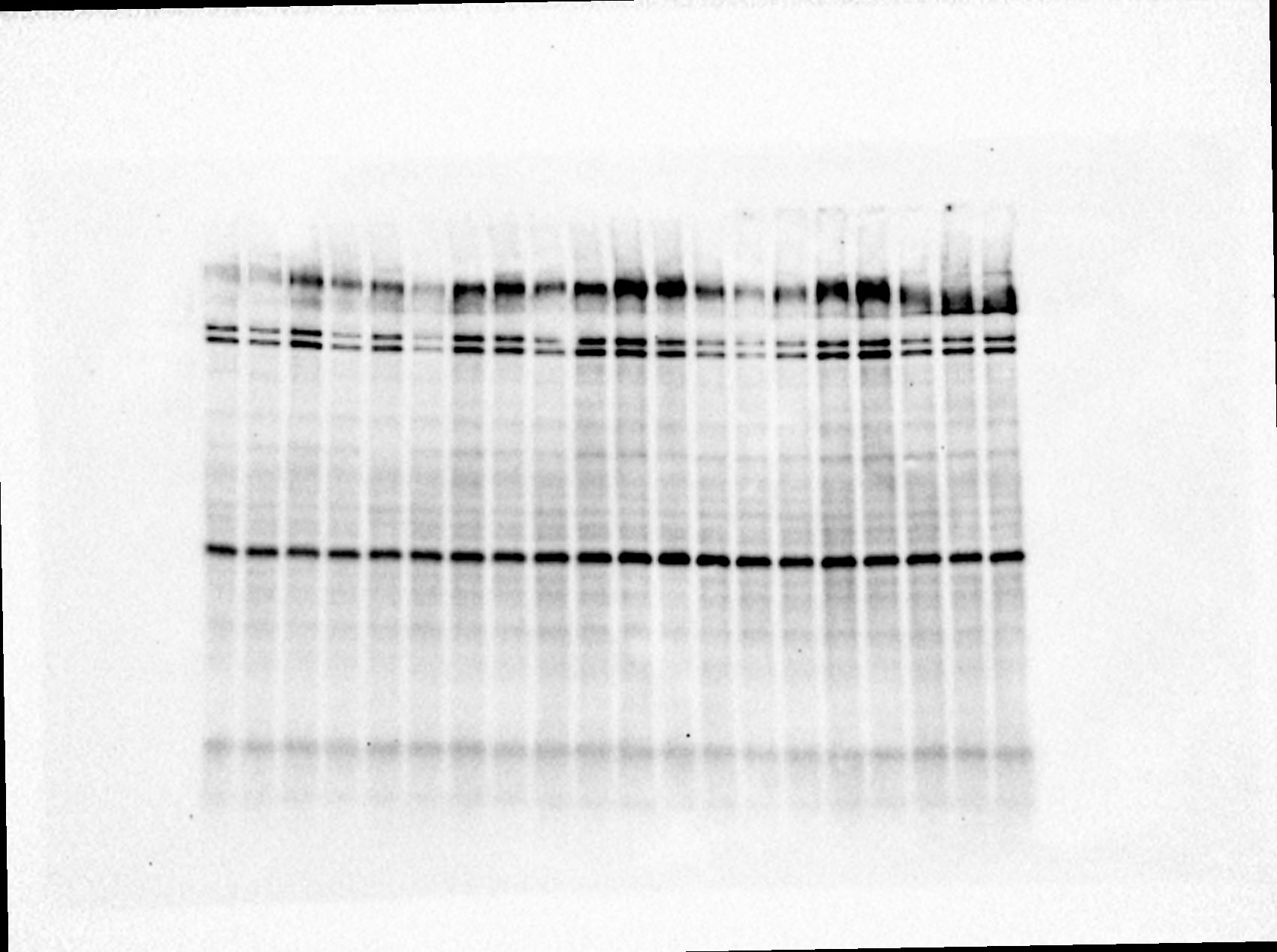

Supplement: Figure 7—source data 1. [file elife-89136-fig7-data1.zip › Figure 7-Source Data/Figure 7-Source Data-1 (raw WB images)/Figure 7C-Col1 & GAPDH.jpg]

## Slide 1
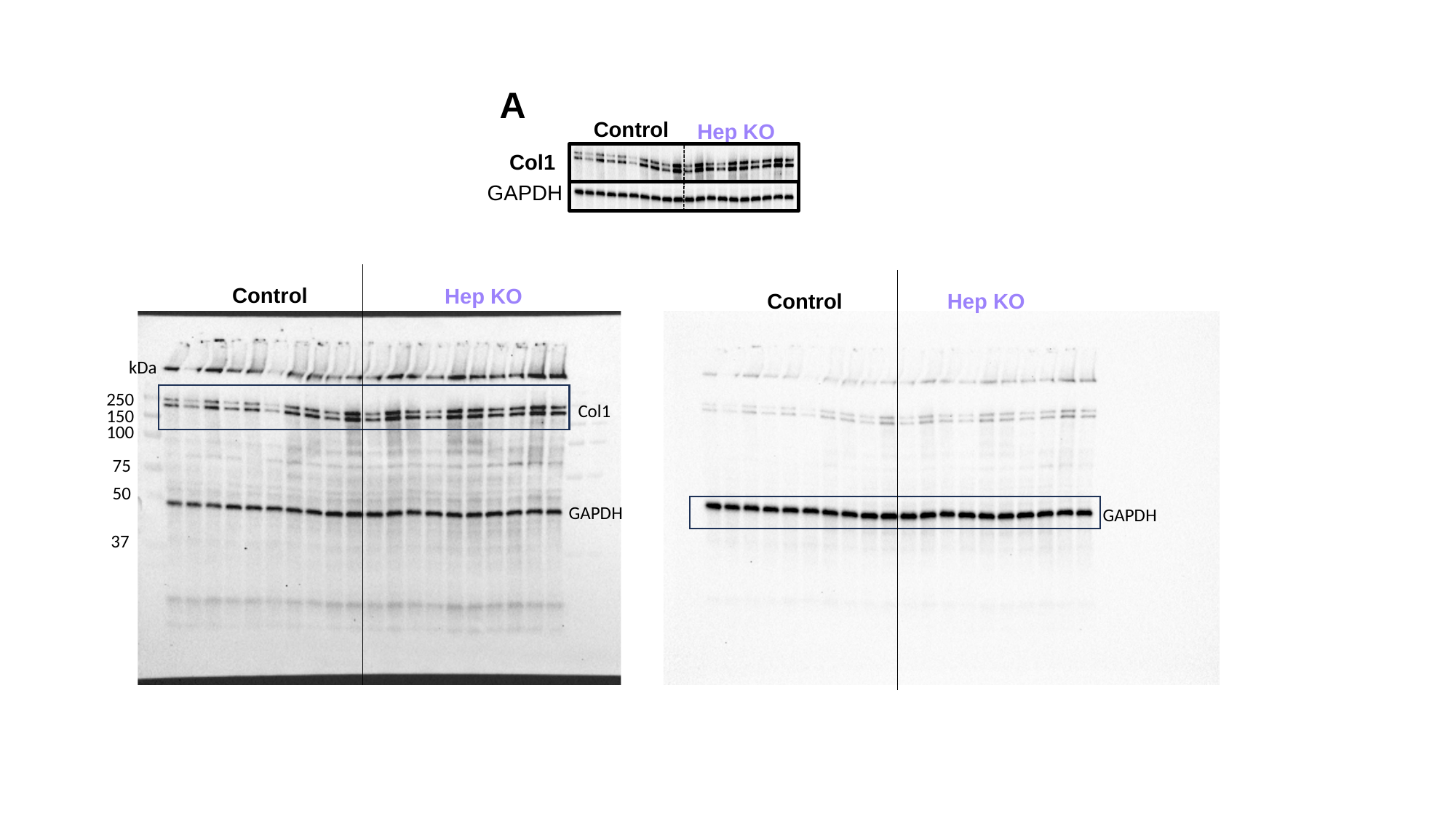

A
Control
Hep KO
Col1
GAPDH
Control
Hep KO
Control
Hep KO
kDa
250
Col1
150
100
75
50
GAPDH
GAPDH
37

Supplement: Figure 7—source data 1. [file elife-89136-fig7-data1.zip › Figure 7-Source Data/Figure 7-Source Data-2 (labeled WB images)/Figure 7A.pptx]

## Slide 1
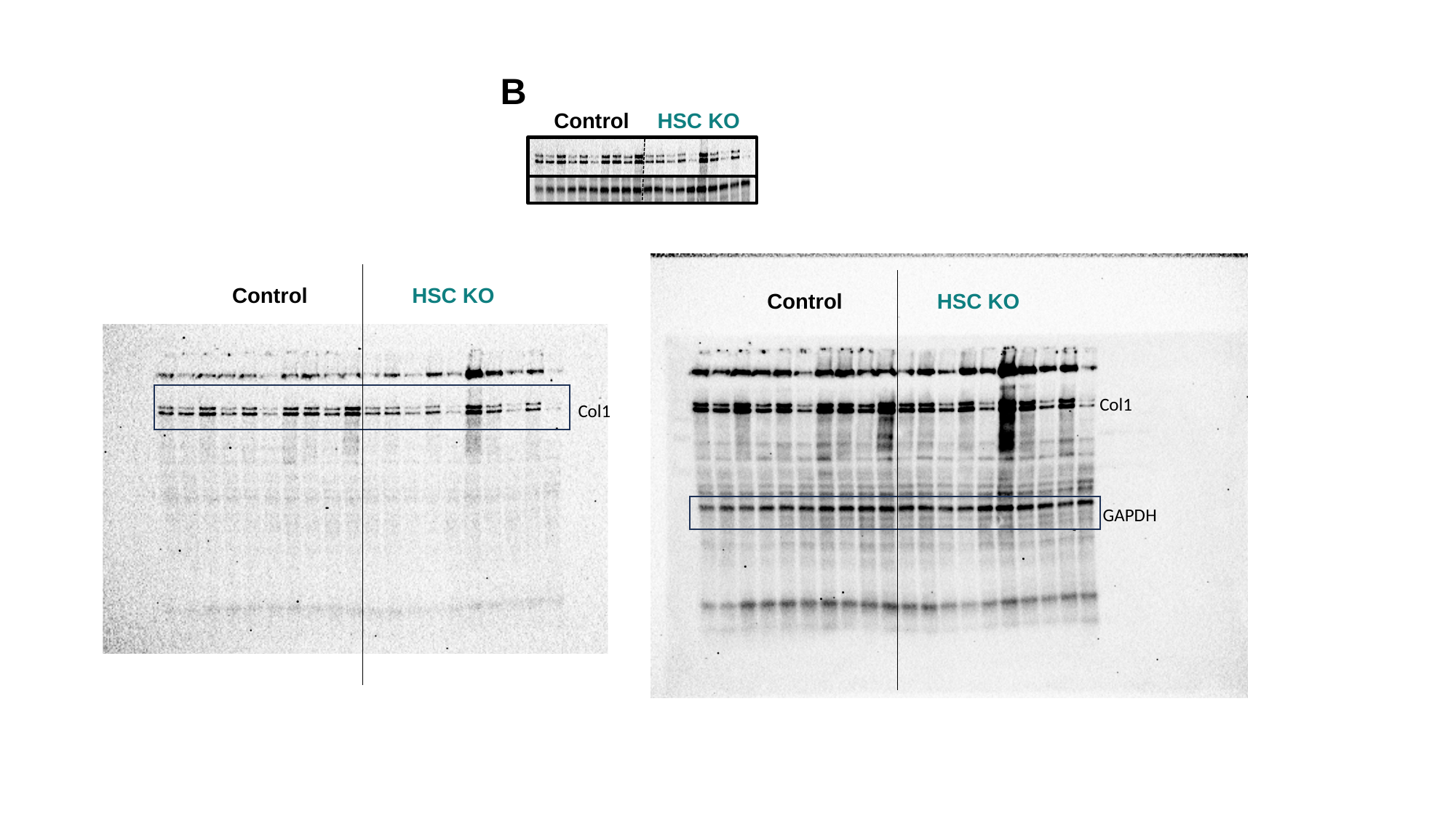

B
HSC KO
Control
Control
HSC KO
Control
HSC KO
Col1
Col1
GAPDH

Supplement: Figure 7—source data 1. [file elife-89136-fig7-data1.zip › Figure 7-Source Data/Figure 7-Source Data-2 (labeled WB images)/Figure 7B.pptx]

## Slide 1
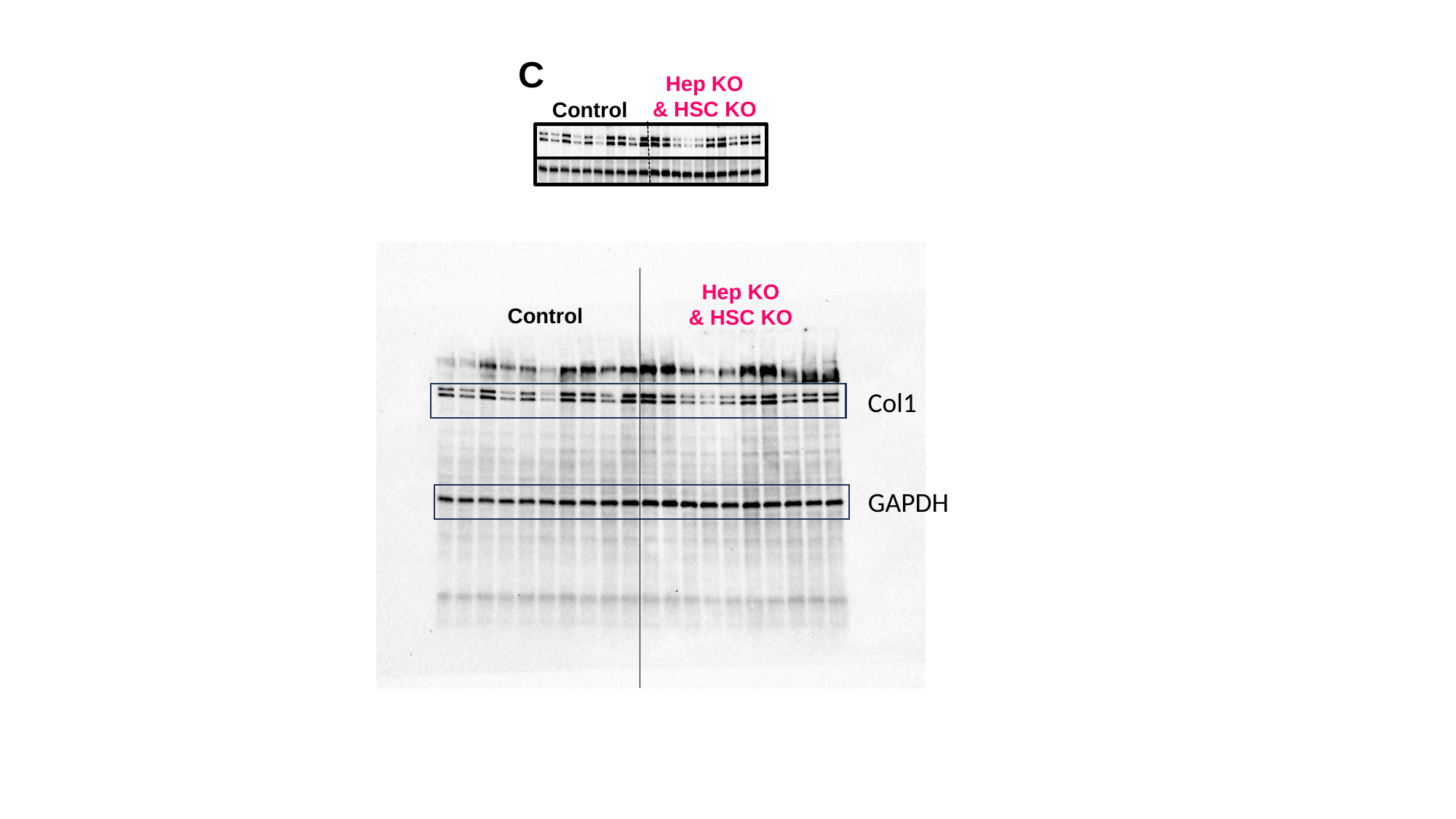

C
Hep KO
& HSC KO
Control
Hep KO
& HSC KO
Control
Col1
GAPDH

Supplement: Figure 7—source data 1. [file elife-89136-fig7-data1.zip › Figure 7-Source Data/Figure 7-Source Data-2 (labeled WB images)/Figure 7C.pptx]

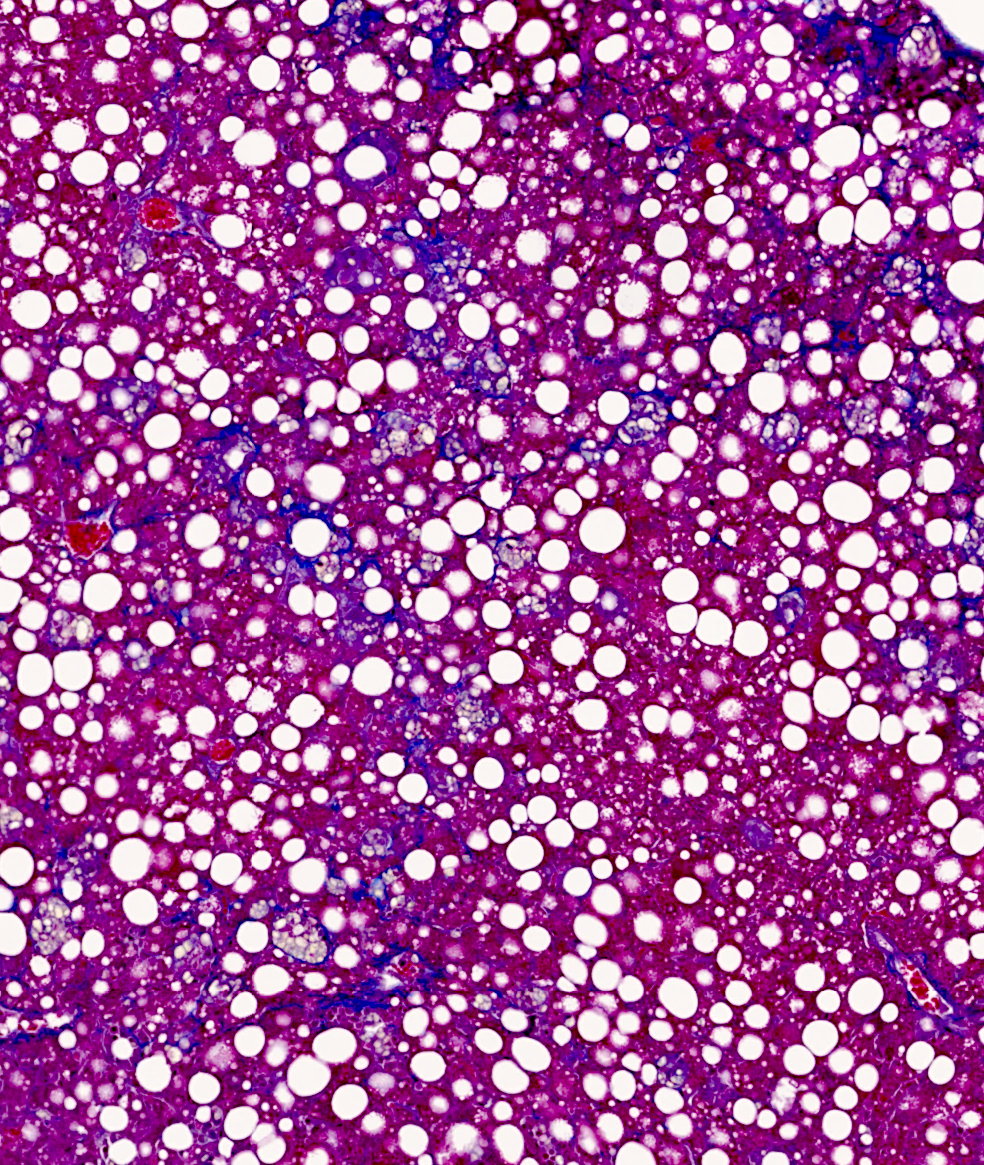

Supplement: Figure 7—source data 1. [file elife-89136-fig7-data1.zip › Figure 7-Source Data/Figure 7-Source Data-3 (raw IHC images)/Figure 7D-Control.tif]

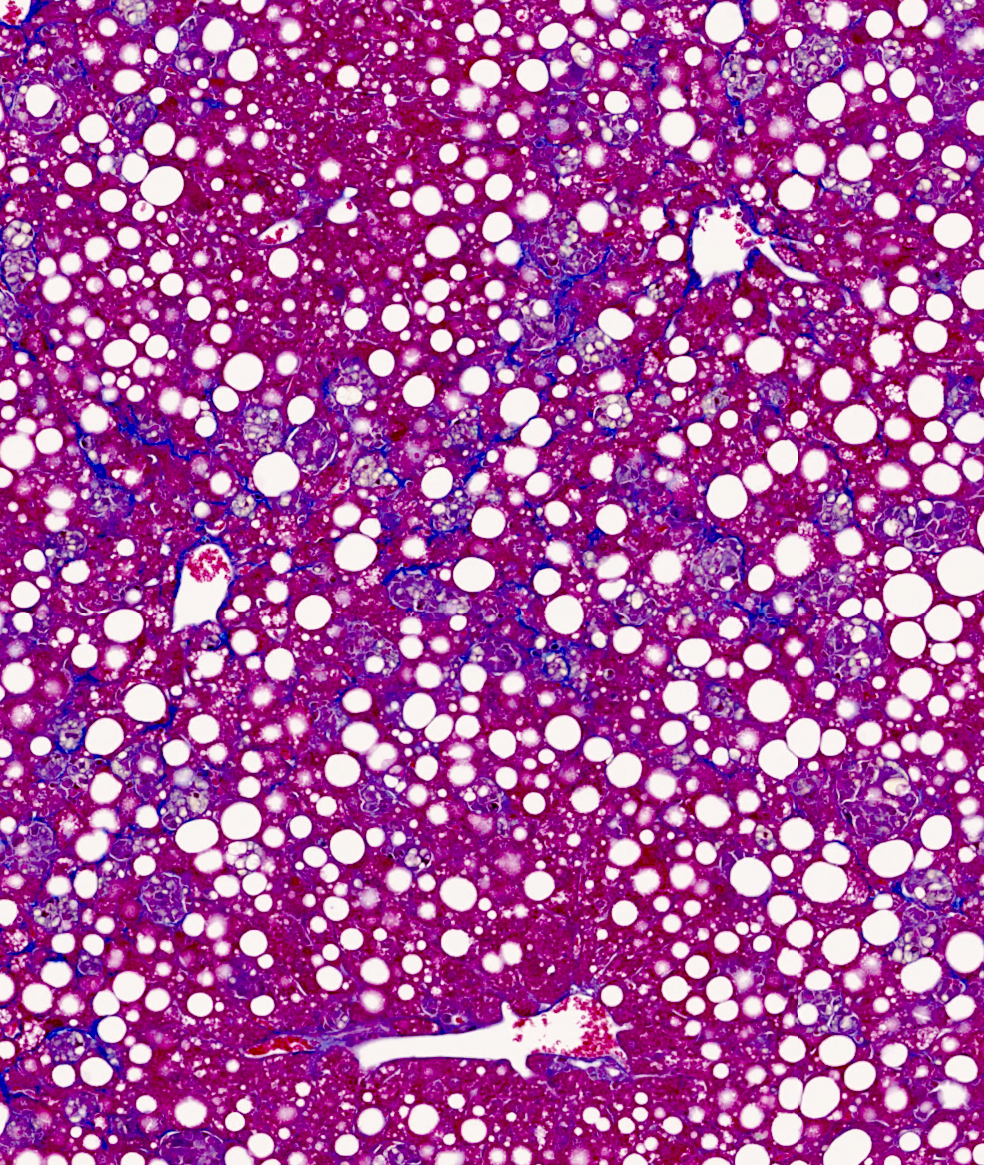

Supplement: Figure 7—source data 1. [file elife-89136-fig7-data1.zip › Figure 7-Source Data/Figure 7-Source Data-3 (raw IHC images)/Figure 7D-Hep KO & HSC KO.tif]

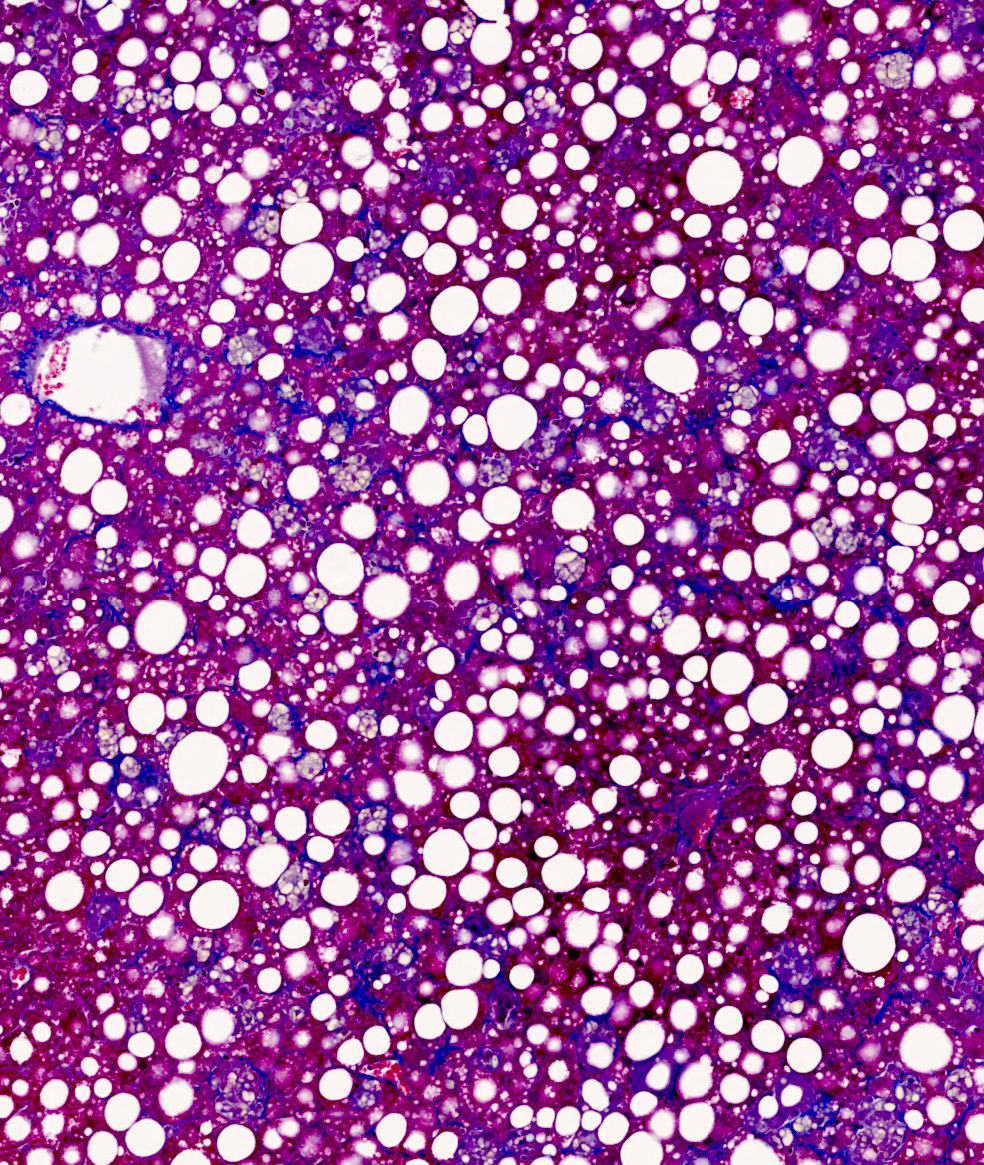

Supplement: Figure 7—source data 1. [file elife-89136-fig7-data1.zip › Figure 7-Source Data/Figure 7-Source Data-3 (raw IHC images)/Figure 7D-Hep KO.tif]

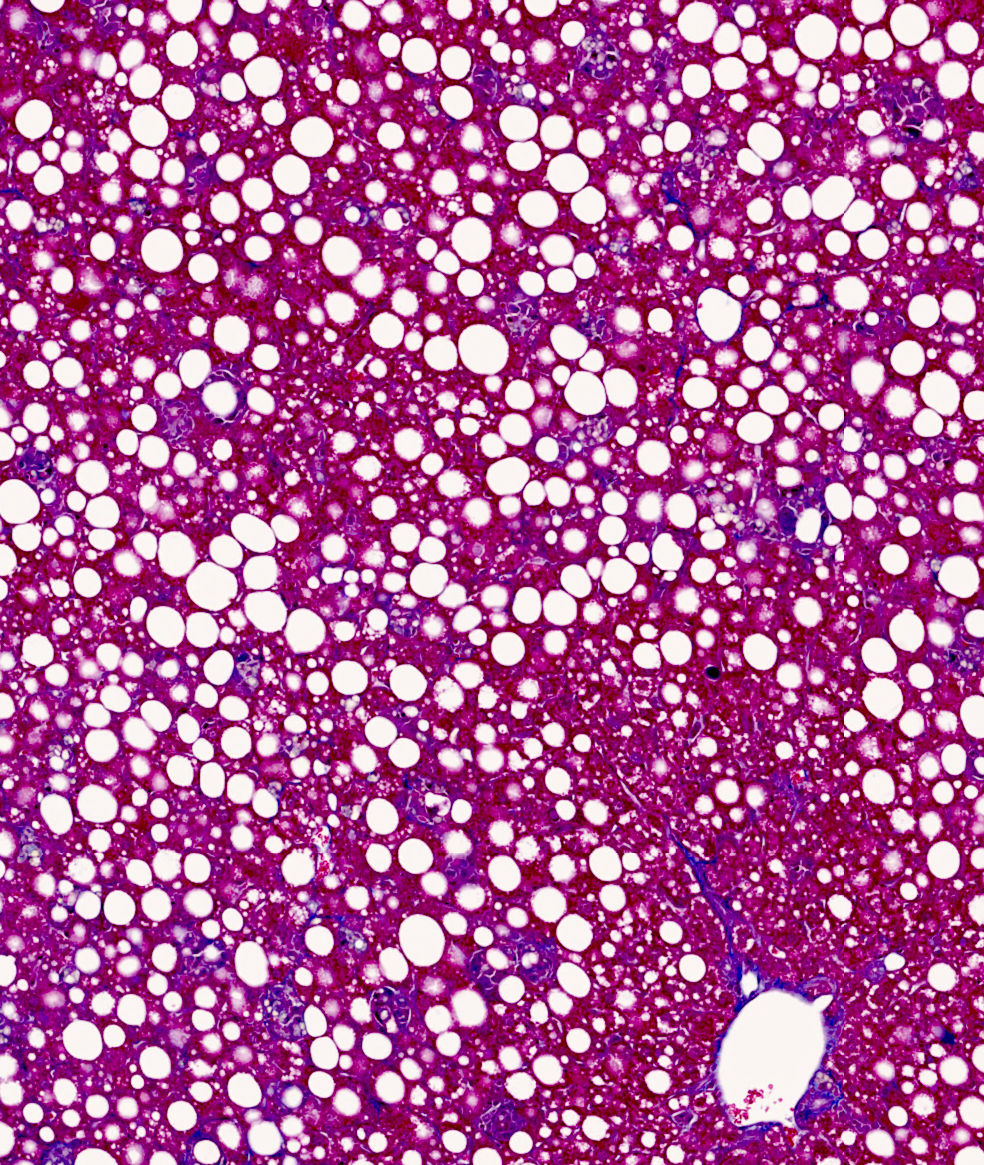

Supplement: Figure 7—source data 1. [file elife-89136-fig7-data1.zip › Figure 7-Source Data/Figure 7-Source Data-3 (raw IHC images)/Figure 7D-HSC KO.tif]

## Slide 1
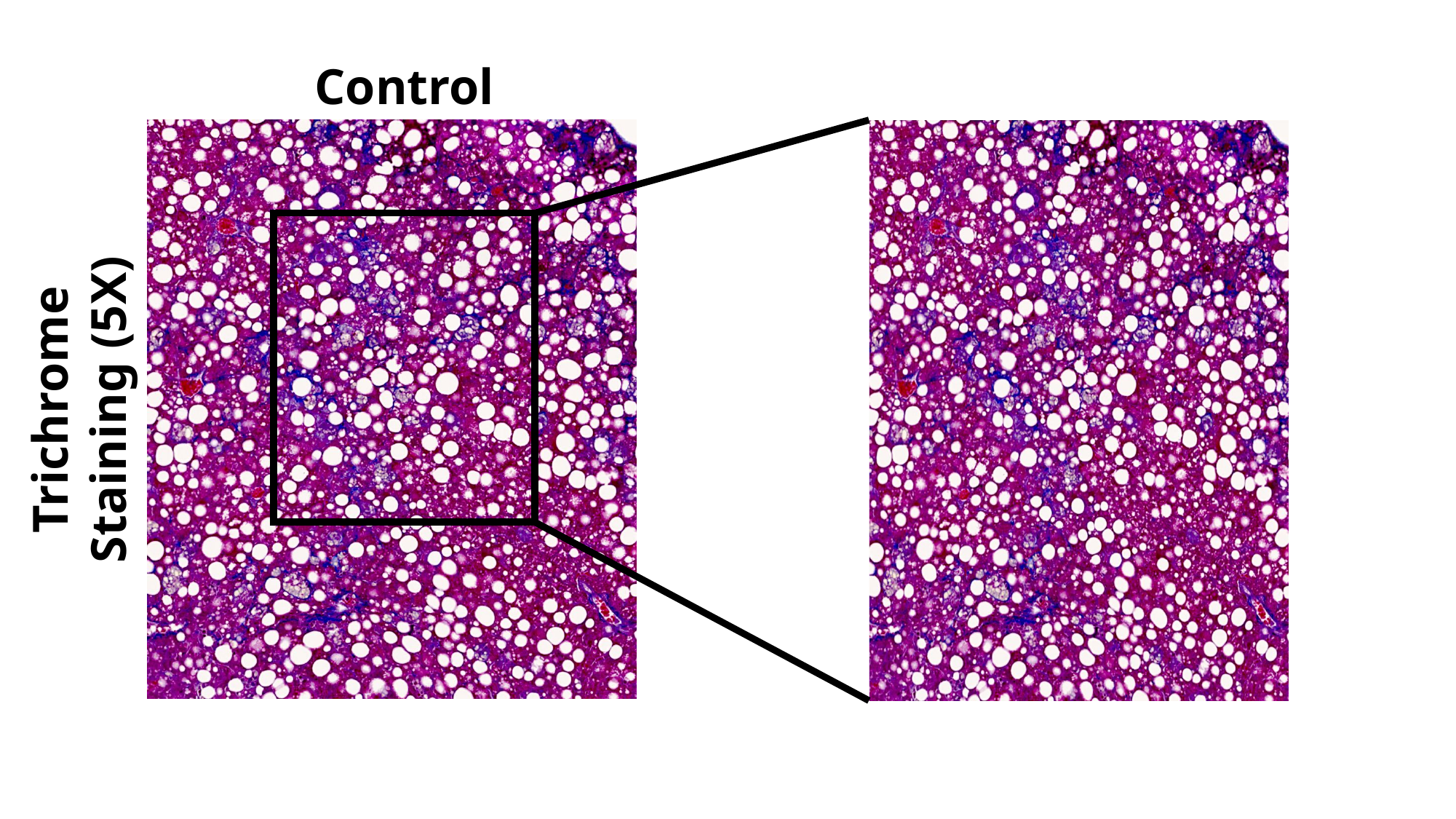

Control
Trichrome
Staining (5X)

Supplement: Figure 7—source data 1. [file elife-89136-fig7-data1.zip › Figure 7-Source Data/Figure 7-Source Data-4 (labeled IHC images)/Figure 7D-Control.pptx]

## Slide 1
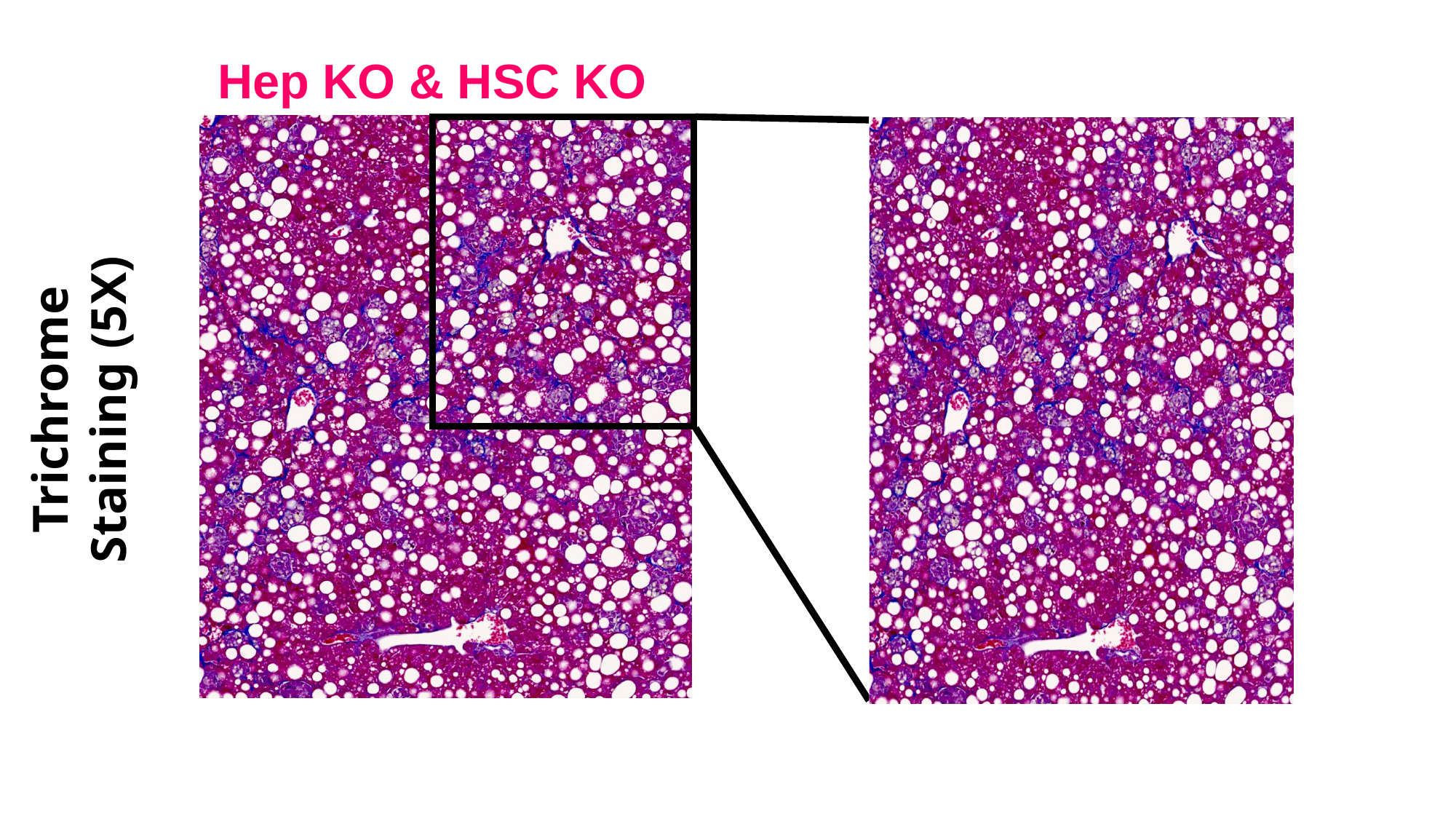

Hep KO & HSC KO
Trichrome
Staining (5X)

Supplement: Figure 7—source data 1. [file elife-89136-fig7-data1.zip › Figure 7-Source Data/Figure 7-Source Data-4 (labeled IHC images)/Figure 7D-Hep KO & HSC KO.pptx]

## Slide 1
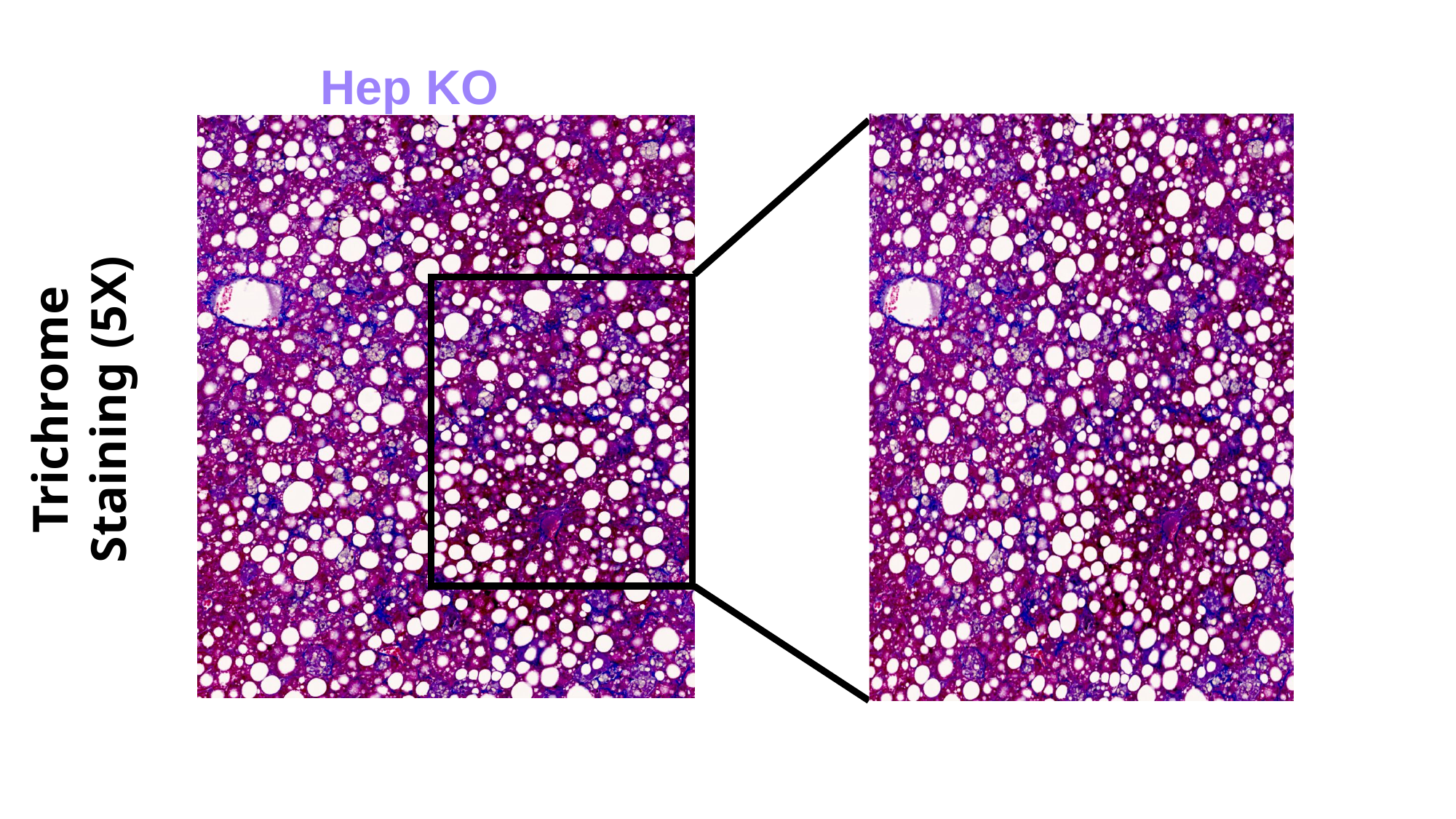

Hep KO
Trichrome
Staining (5X)

Supplement: Figure 7—source data 1. [file elife-89136-fig7-data1.zip › Figure 7-Source Data/Figure 7-Source Data-4 (labeled IHC images)/Figure 7D-Hep KO.pptx]

## Slide 1
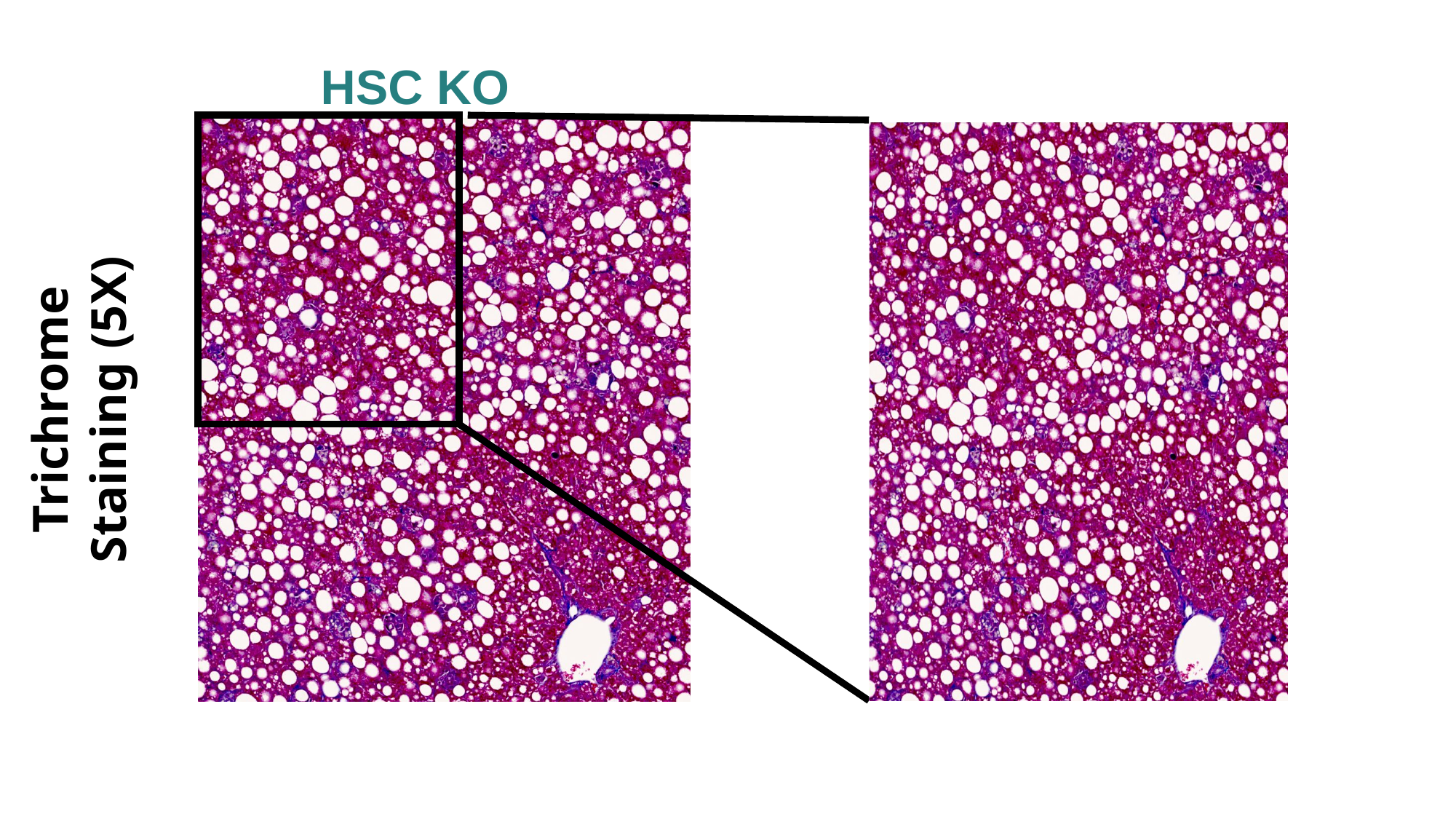

HSC KO
Trichrome
Staining (5X)

Supplement: Figure 7—source data 1. [file elife-89136-fig7-data1.zip › Figure 7-Source Data/Figure 7-Source Data-4 (labeled IHC images)/Figure 7D-HSC KO.pptx]

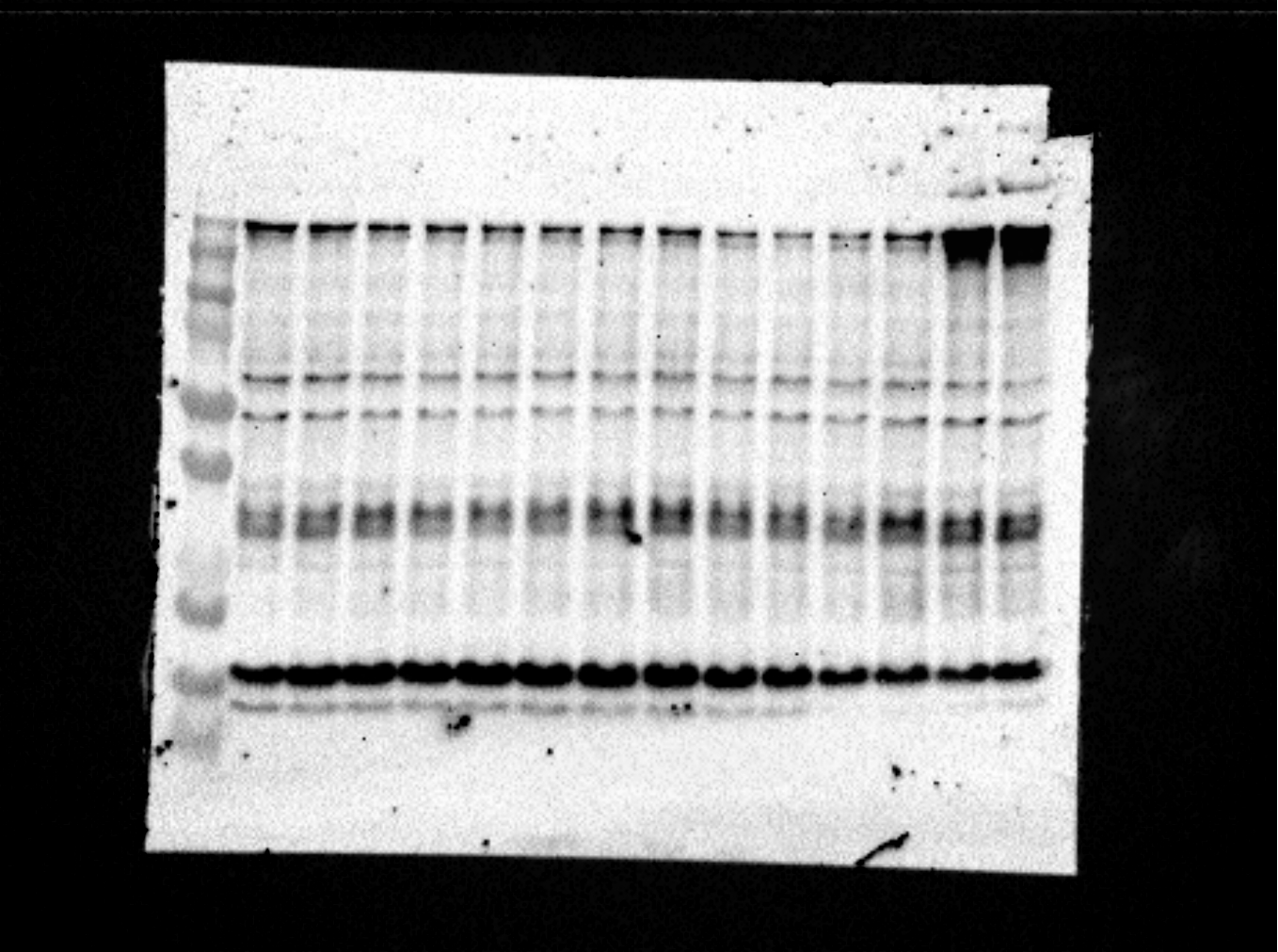

Supplement: Figure 8—source data 1. [file elife-89136-fig8-data1.zip › Figure 8-Source Data/Figure 8-Source Data-2 (raw WB images)/Figure 8B-COL1.jpg]

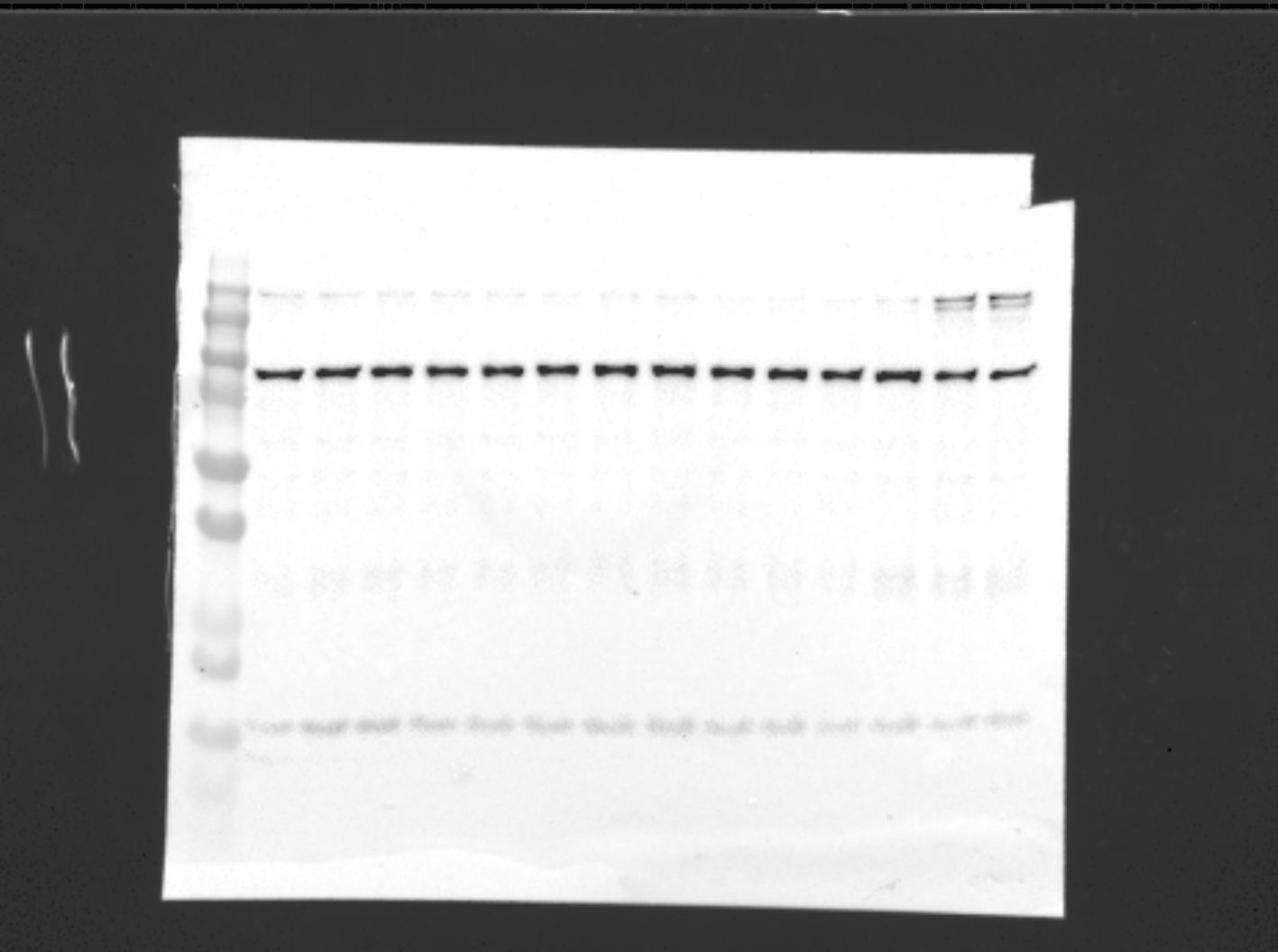

Supplement: Figure 8—source data 1. [file elife-89136-fig8-data1.zip › Figure 8-Source Data/Figure 8-Source Data-2 (raw WB images)/Figure 8B-HSP90.jpg]

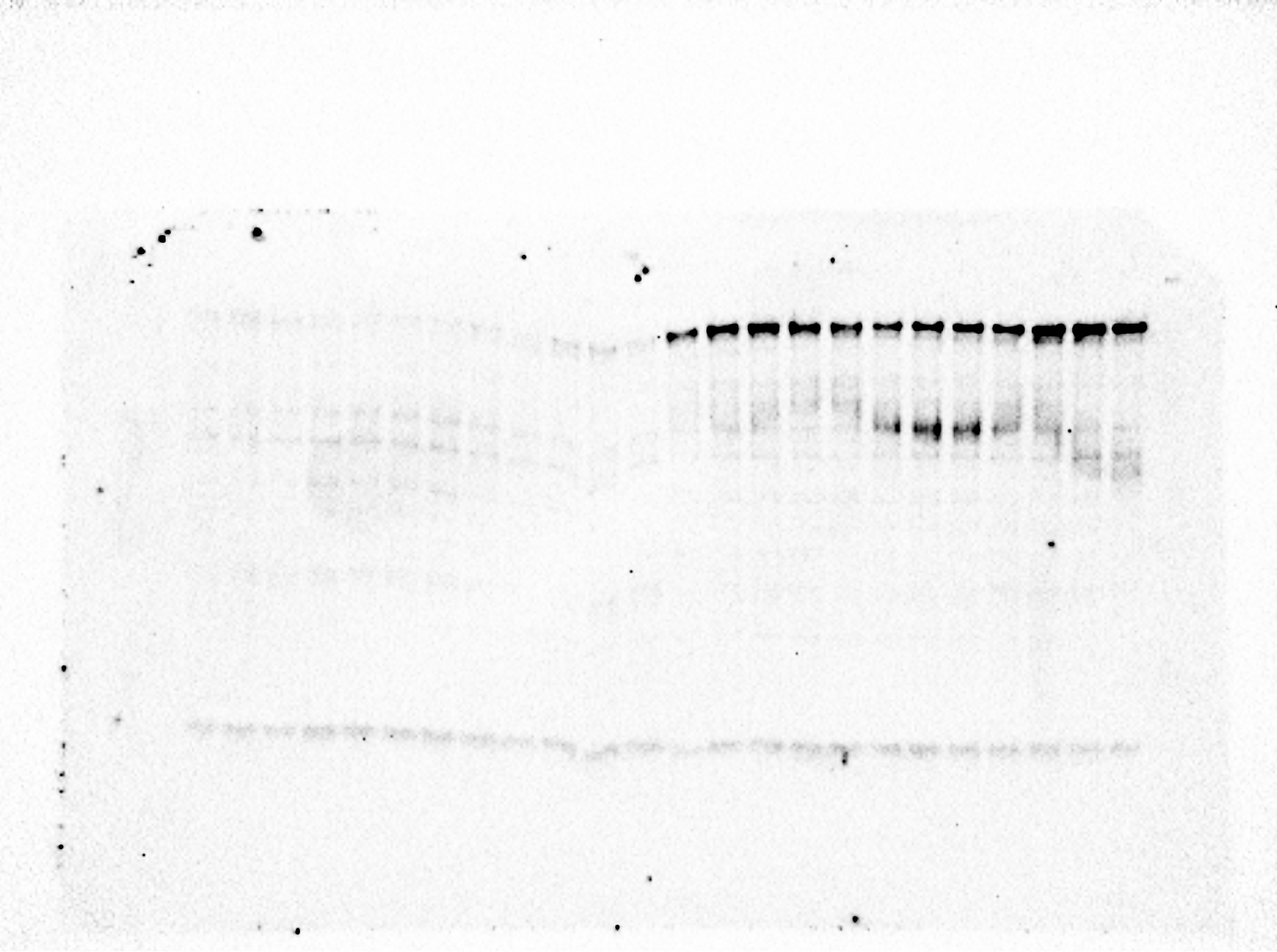

Supplement: Figure 8—source data 1. [file elife-89136-fig8-data1.zip › Figure 8-Source Data/Figure 8-Source Data-2 (raw WB images)/Figure 8C-COL1.jpg]

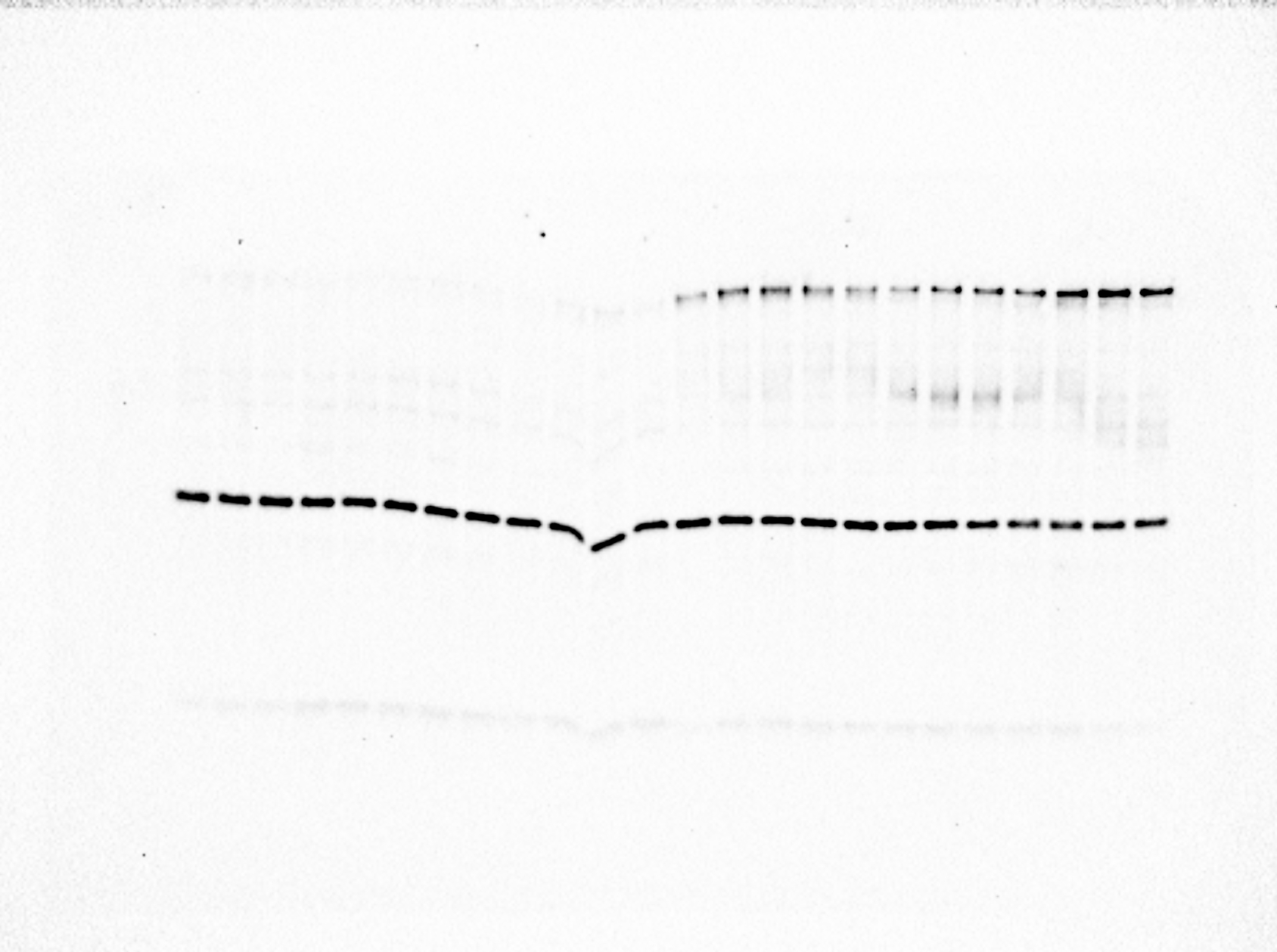

Supplement: Figure 8—source data 1. [file elife-89136-fig8-data1.zip › Figure 8-Source Data/Figure 8-Source Data-2 (raw WB images)/Figure 8C-GAPDH.jpg]

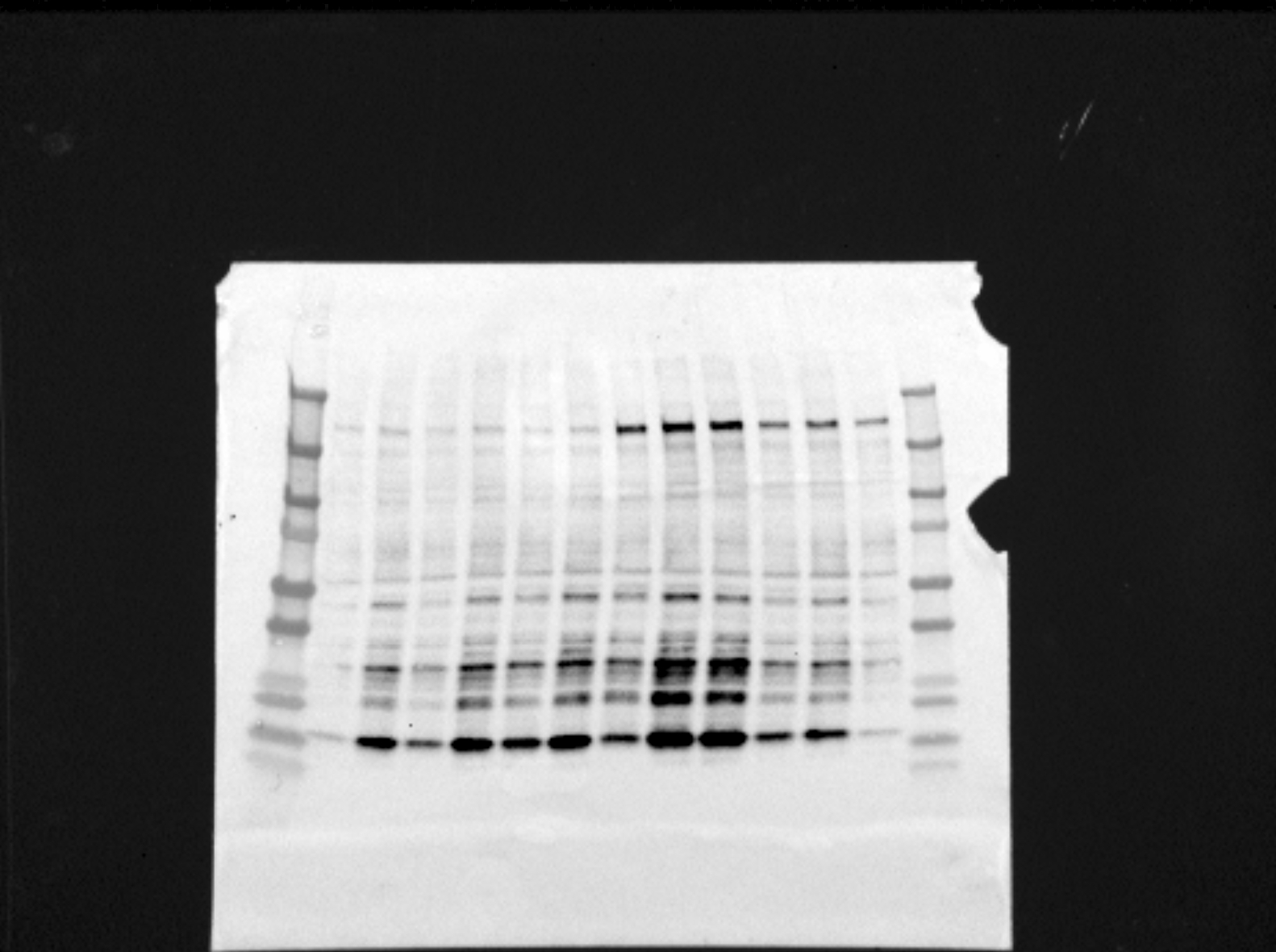

Supplement: Figure 8—source data 1. [file elife-89136-fig8-data1.zip › Figure 8-Source Data/Figure 8-Source Data-2 (raw WB images)/Figure 8F-COL1.jpg]

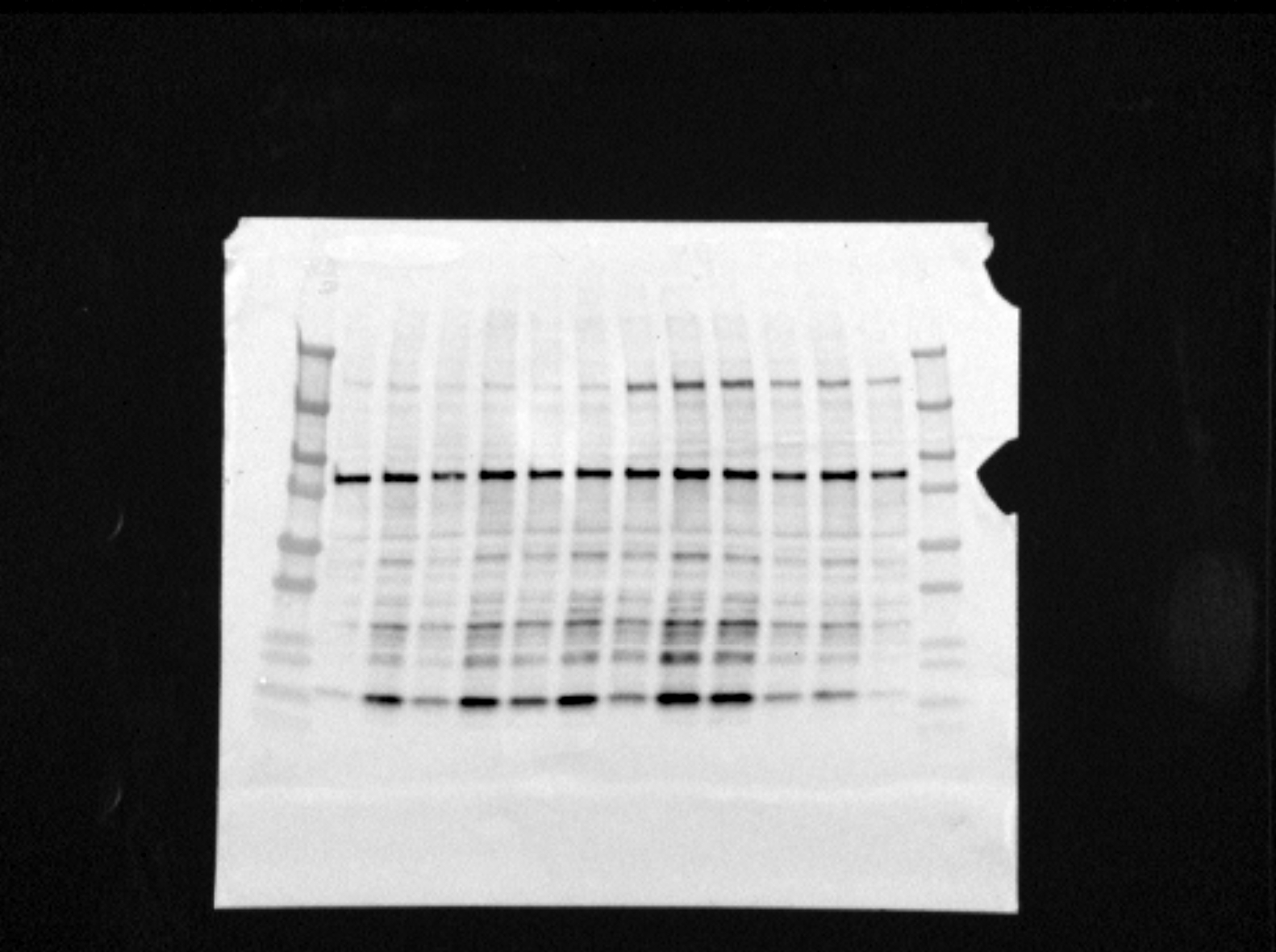

Supplement: Figure 8—source data 1. [file elife-89136-fig8-data1.zip › Figure 8-Source Data/Figure 8-Source Data-2 (raw WB images)/Figure 8F-HSP90.jpg]

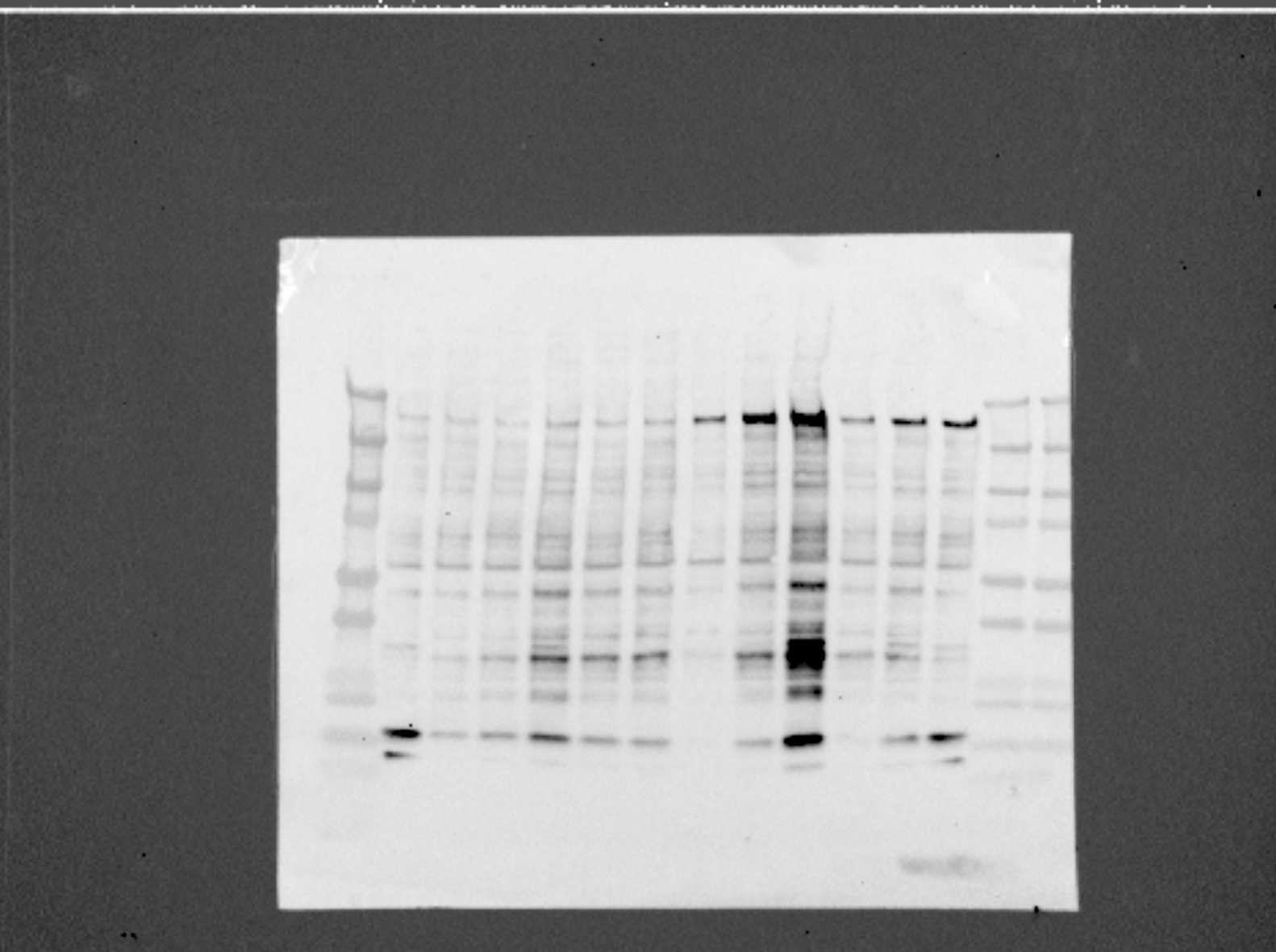

Supplement: Figure 8—source data 1. [file elife-89136-fig8-data1.zip › Figure 8-Source Data/Figure 8-Source Data-2 (raw WB images)/Figure 8G-COL1.jpg]

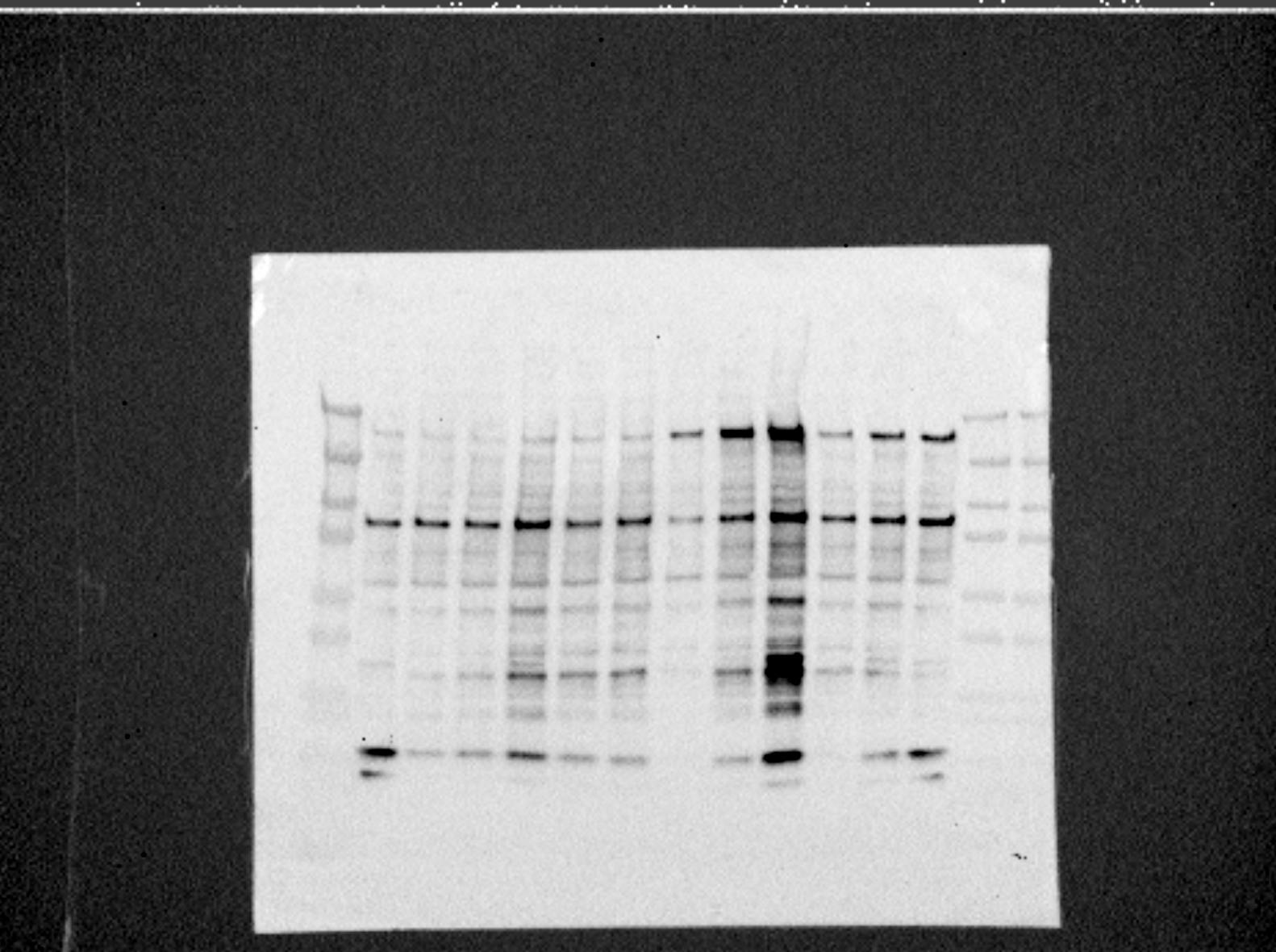

Supplement: Figure 8—source data 1. [file elife-89136-fig8-data1.zip › Figure 8-Source Data/Figure 8-Source Data-2 (raw WB images)/Figure 8G-HSP90.jpg]

## Slide 1
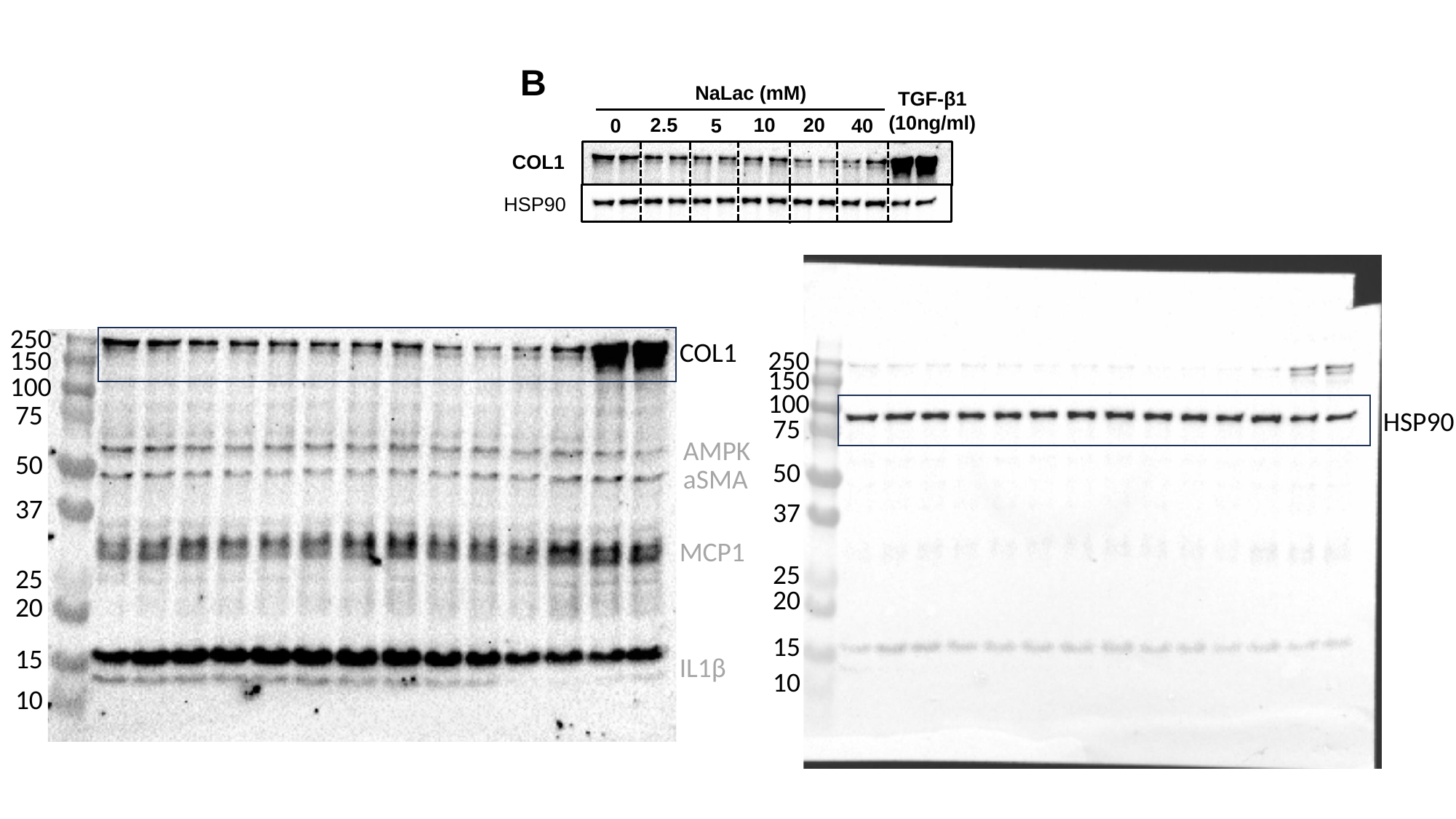

B
NaLac (mM)
TGF-β1
(10ng/ml)
2.5
10
20
5
0
40
COL1
HSP90
250
150
100
75
50
37
25
20
15
10
HSP90
250
COL1
150
100
75
AMPK
50
aSMA
37
MCP1
25
20
15
IL1β
10

Supplement: Figure 8—source data 1. [file elife-89136-fig8-data1.zip › Figure 8-Source Data/Figure 8-Source Data-3 (labeled WB images)/Figuer 8B.pptx]

## Slide 1
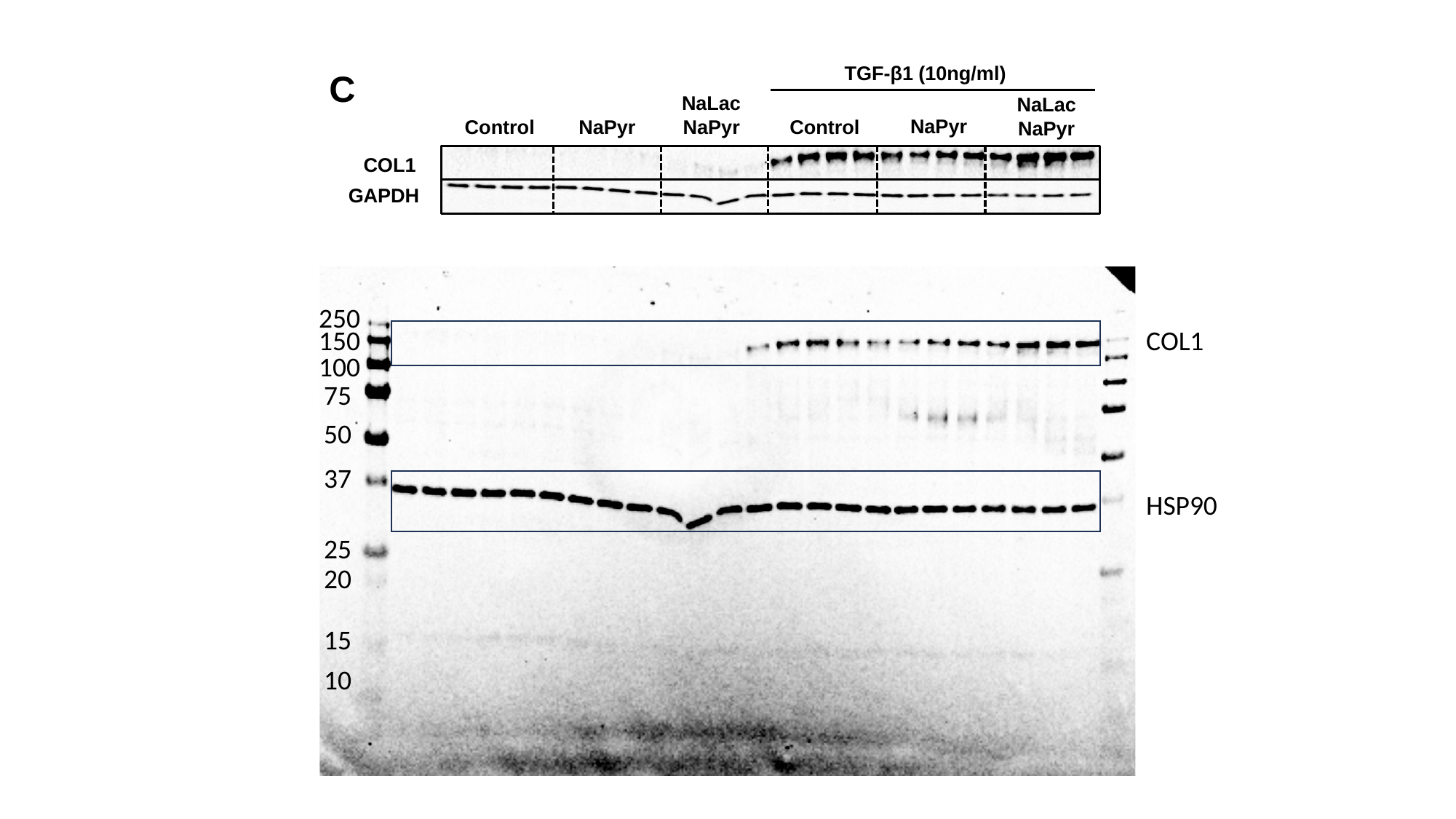

TGF-β1 (10ng/ml)
NaLac
NaPyr
NaLac
NaPyr
NaPyr
NaPyr
Control
Control
COL1
GAPDH
C
250
COL1
150
100
75
50
37
HSP90
25
20
15
10

Supplement: Figure 8—source data 1. [file elife-89136-fig8-data1.zip › Figure 8-Source Data/Figure 8-Source Data-3 (labeled WB images)/Figuer 8C.pptx]

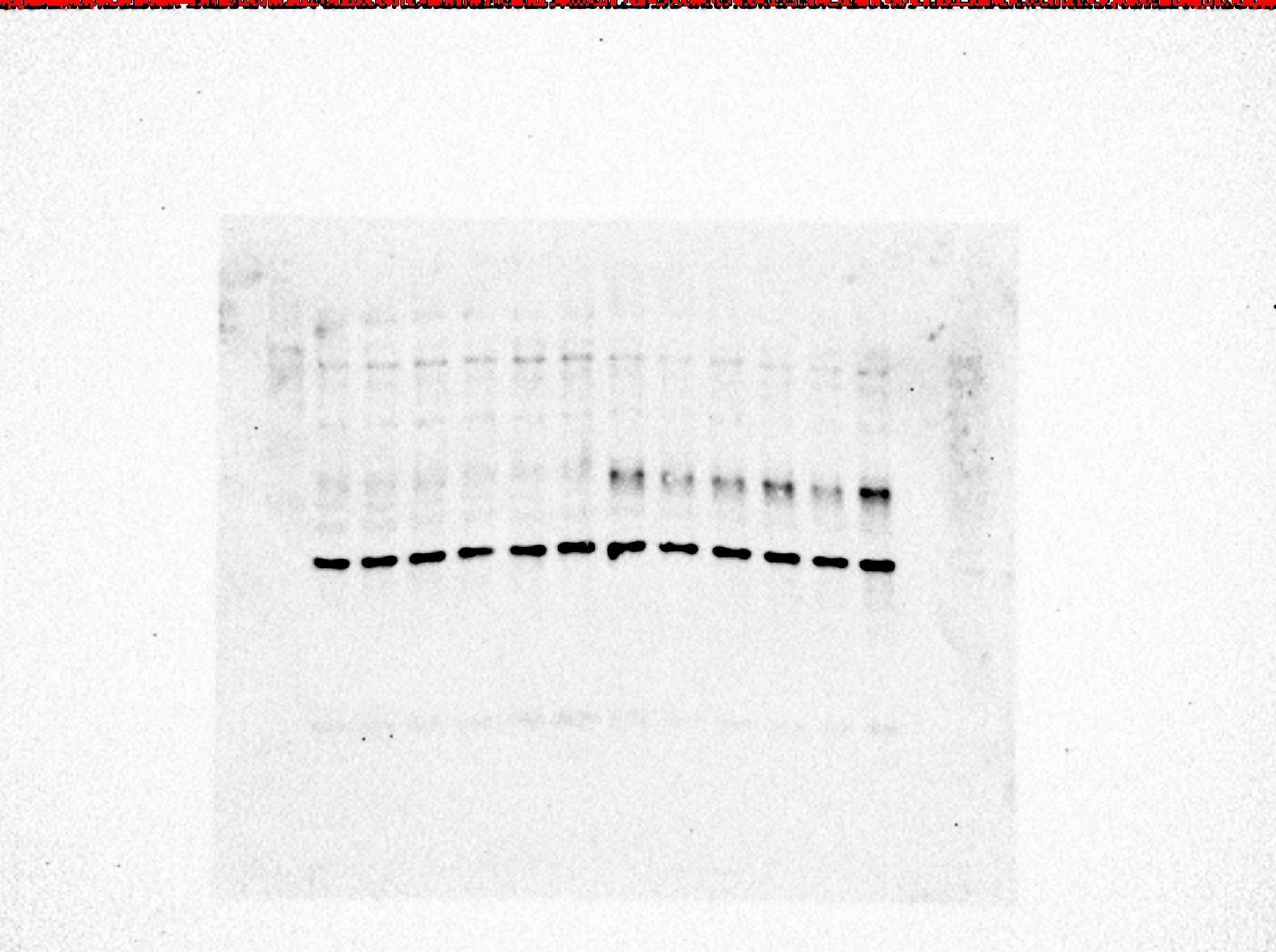

Supplement: Figure 8—figure supplement 1—source data 1. [file elife-89136-fig8-figsupp1-data1.zip › Figure 8-figure supplement 1-Source Data/Figure 8-figure supplement 1-Source Data-1 (raw WB images)/Figure 8-figure supplement 1A-GAPDH.jpg]

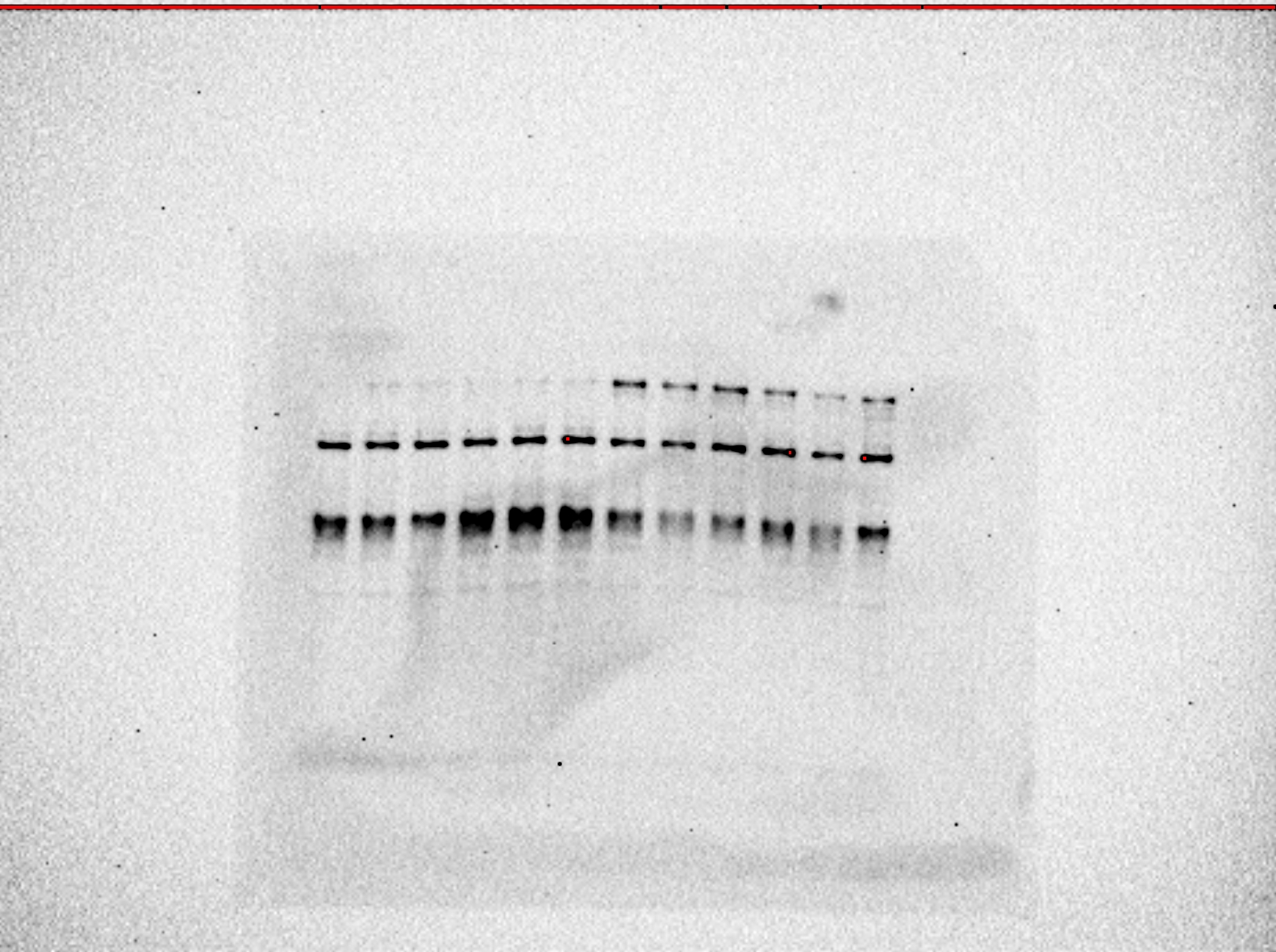

Supplement: Figure 8—figure supplement 1—source data 1. [file elife-89136-fig8-figsupp1-data1.zip › Figure 8-figure supplement 1-Source Data/Figure 8-figure supplement 1-Source Data-1 (raw WB images)/Figure 8-figure supplement 1A-HSP90-SMAD3.jpg]

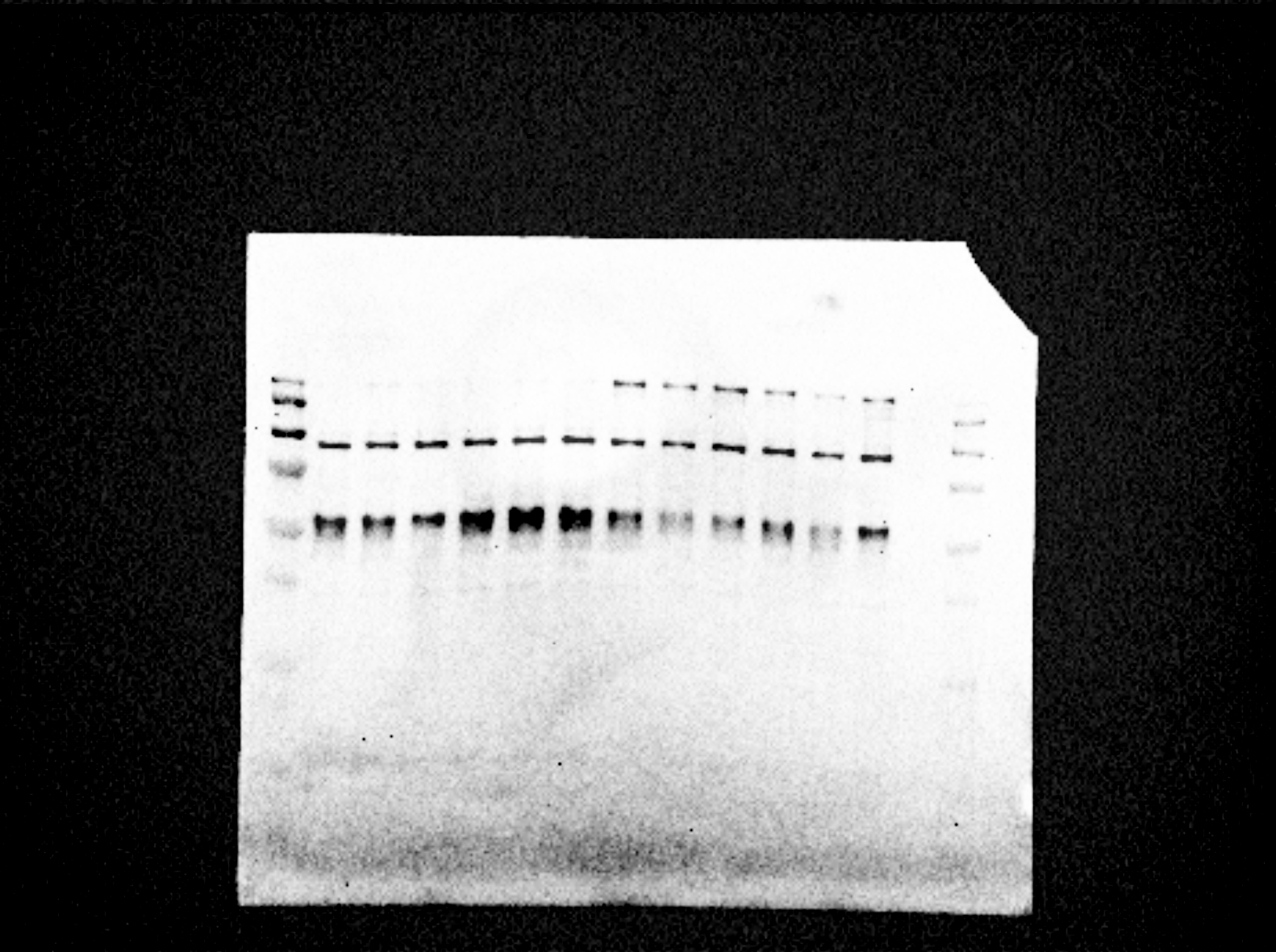

Supplement: Figure 8—figure supplement 1—source data 1. [file elife-89136-fig8-figsupp1-data1.zip › Figure 8-figure supplement 1-Source Data/Figure 8-figure supplement 1-Source Data-1 (raw WB images)/Figure 8-figure supplement 1A-HSP90-SMAD3-ladder merged.jpg]

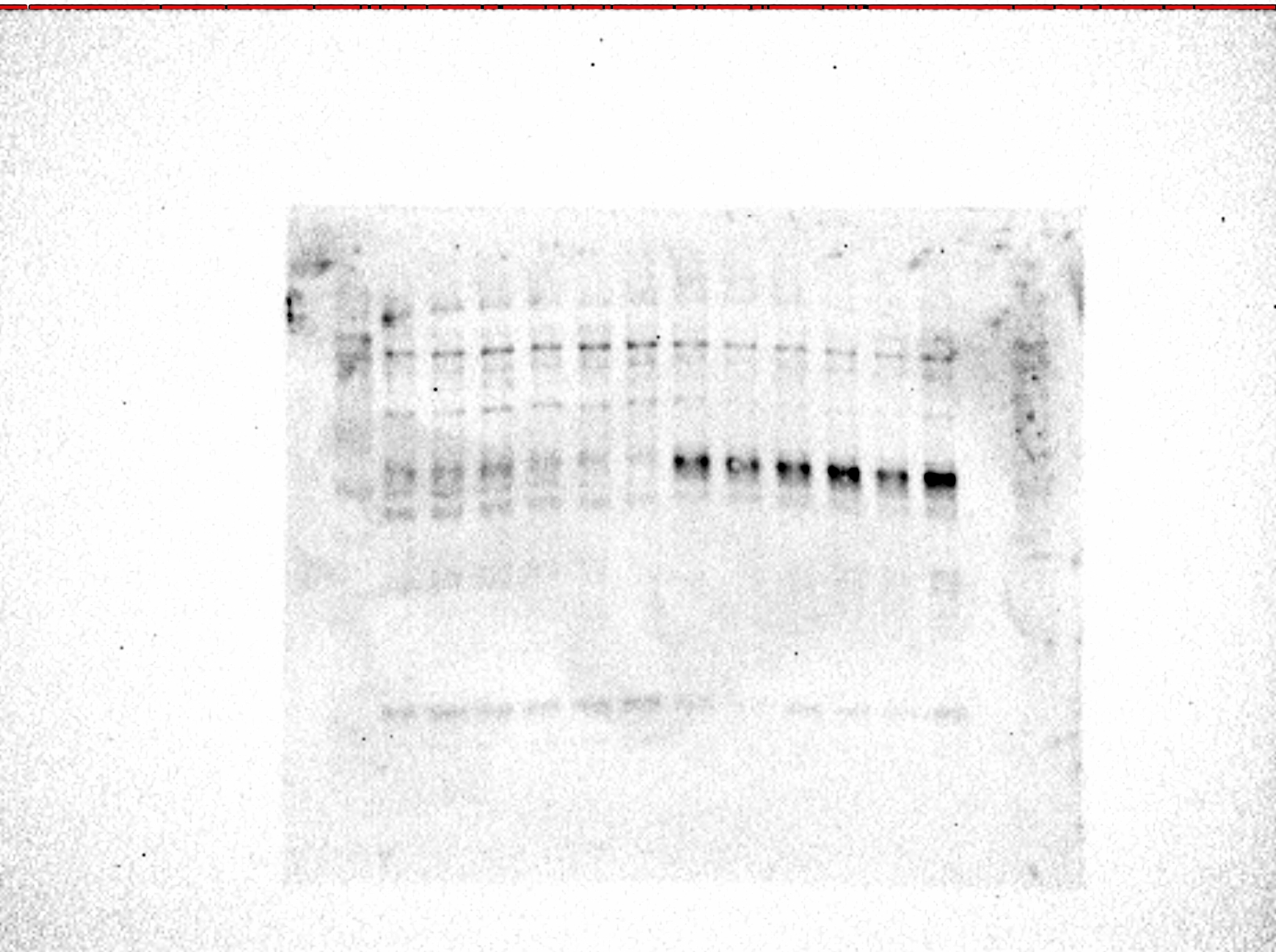

Supplement: Figure 8—figure supplement 1—source data 1. [file elife-89136-fig8-figsupp1-data1.zip › Figure 8-figure supplement 1-Source Data/Figure 8-figure supplement 1-Source Data-1 (raw WB images)/Figure 8-figure supplement 1A-pSMAD3.jpg]

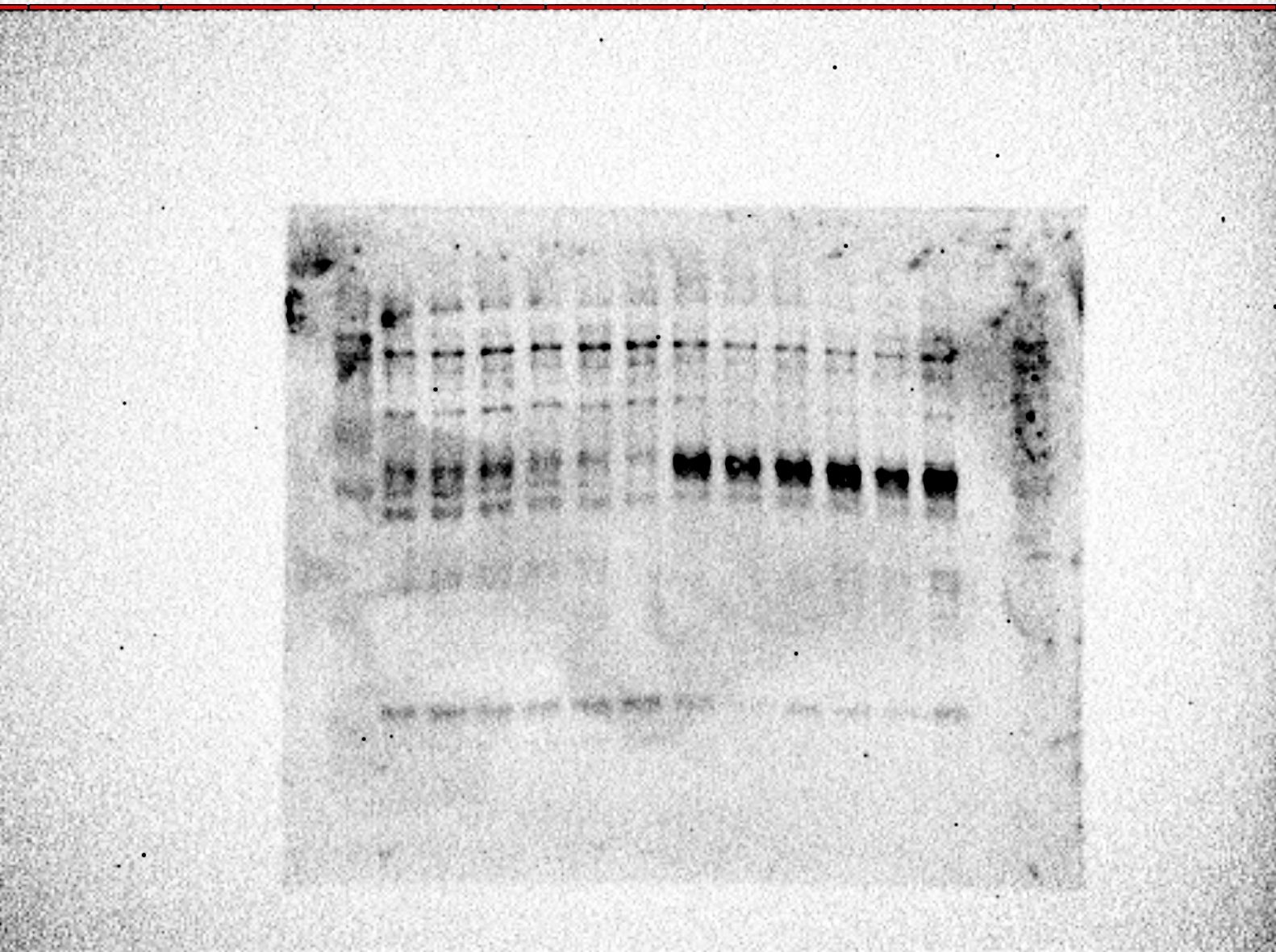

Supplement: Figure 8—figure supplement 1—source data 1. [file elife-89136-fig8-figsupp1-data1.zip › Figure 8-figure supplement 1-Source Data/Figure 8-figure supplement 1-Source Data-1 (raw WB images)/Figure 8-figure supplement 1A-pSMAD3-ladder-merged.jpg]

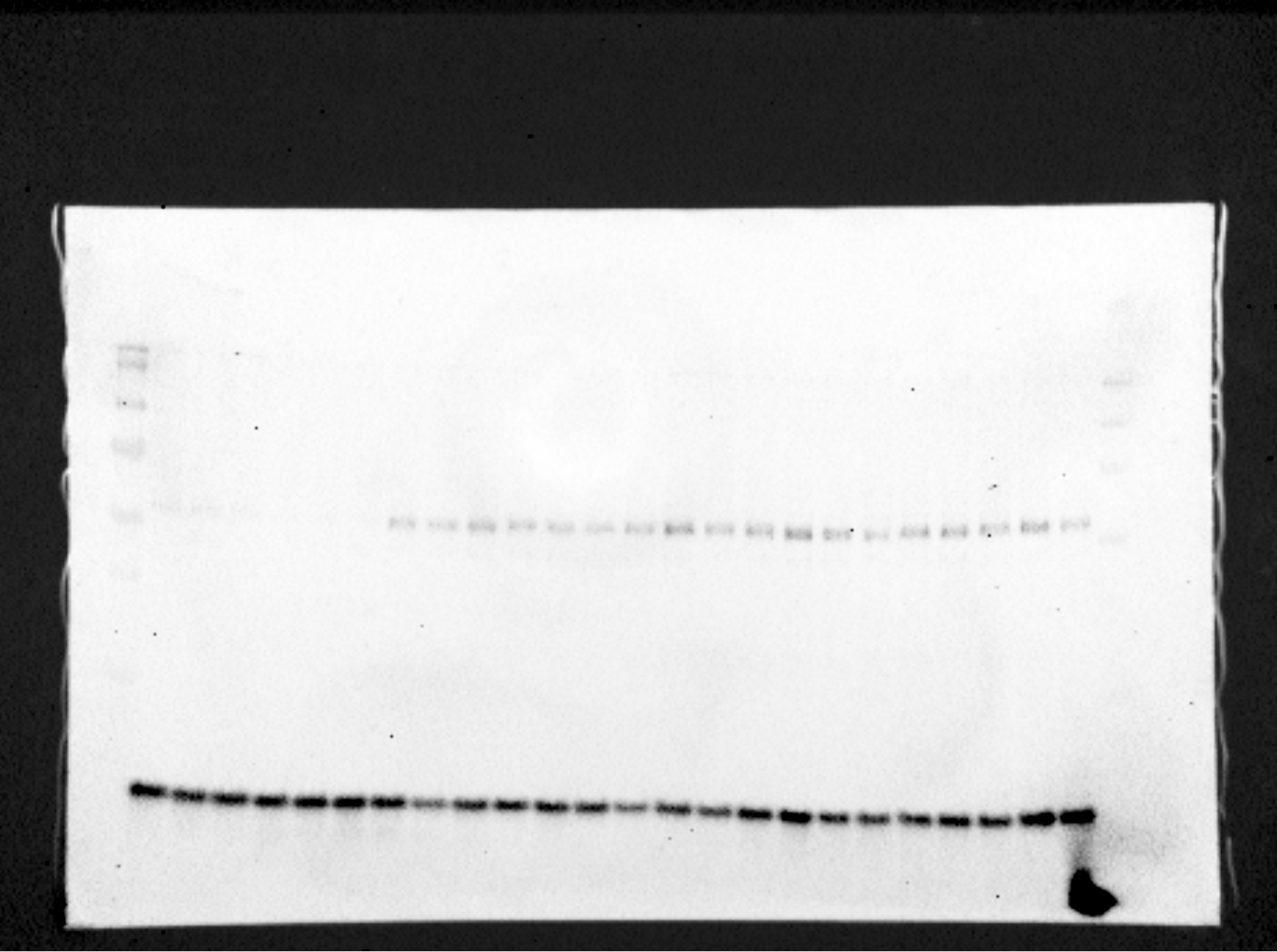

Supplement: Figure 8—figure supplement 1—source data 1. [file elife-89136-fig8-figsupp1-data1.zip › Figure 8-figure supplement 1-Source Data/Figure 8-figure supplement 1-Source Data-1 (raw WB images)/Figure 8-figure supplement 1C-(pSMAD3)-H3.jpg]

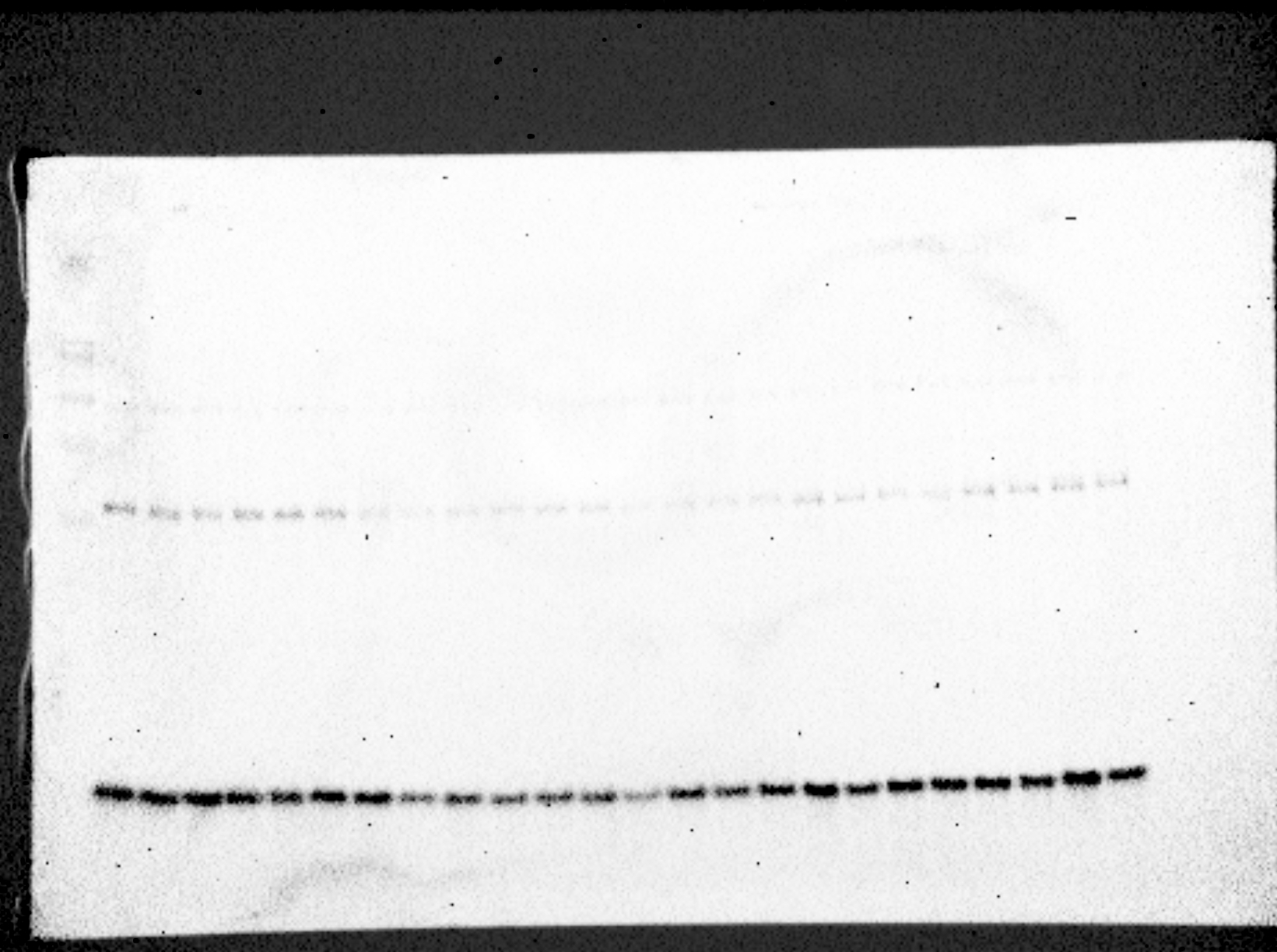

Supplement: Figure 8—figure supplement 1—source data 1. [file elife-89136-fig8-figsupp1-data1.zip › Figure 8-figure supplement 1-Source Data/Figure 8-figure supplement 1-Source Data-1 (raw WB images)/Figure 8-figure supplement 1C-(SMAD3)-H3.jpg]

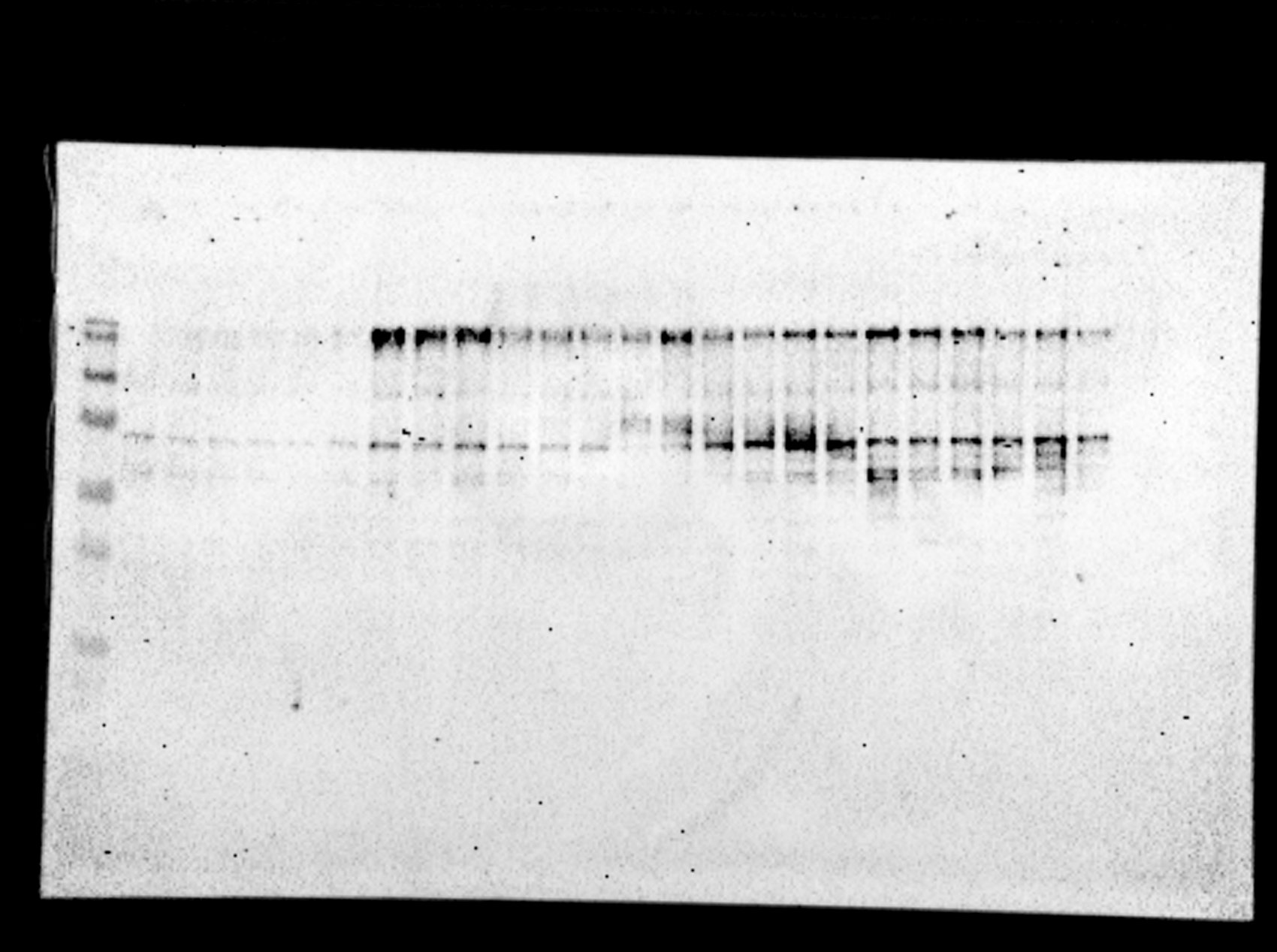

Supplement: Figure 8—figure supplement 1—source data 1. [file elife-89136-fig8-figsupp1-data1.zip › Figure 8-figure supplement 1-Source Data/Figure 8-figure supplement 1-Source Data-1 (raw WB images)/Figure 8-figure supplement 1C-COL1.jpg]

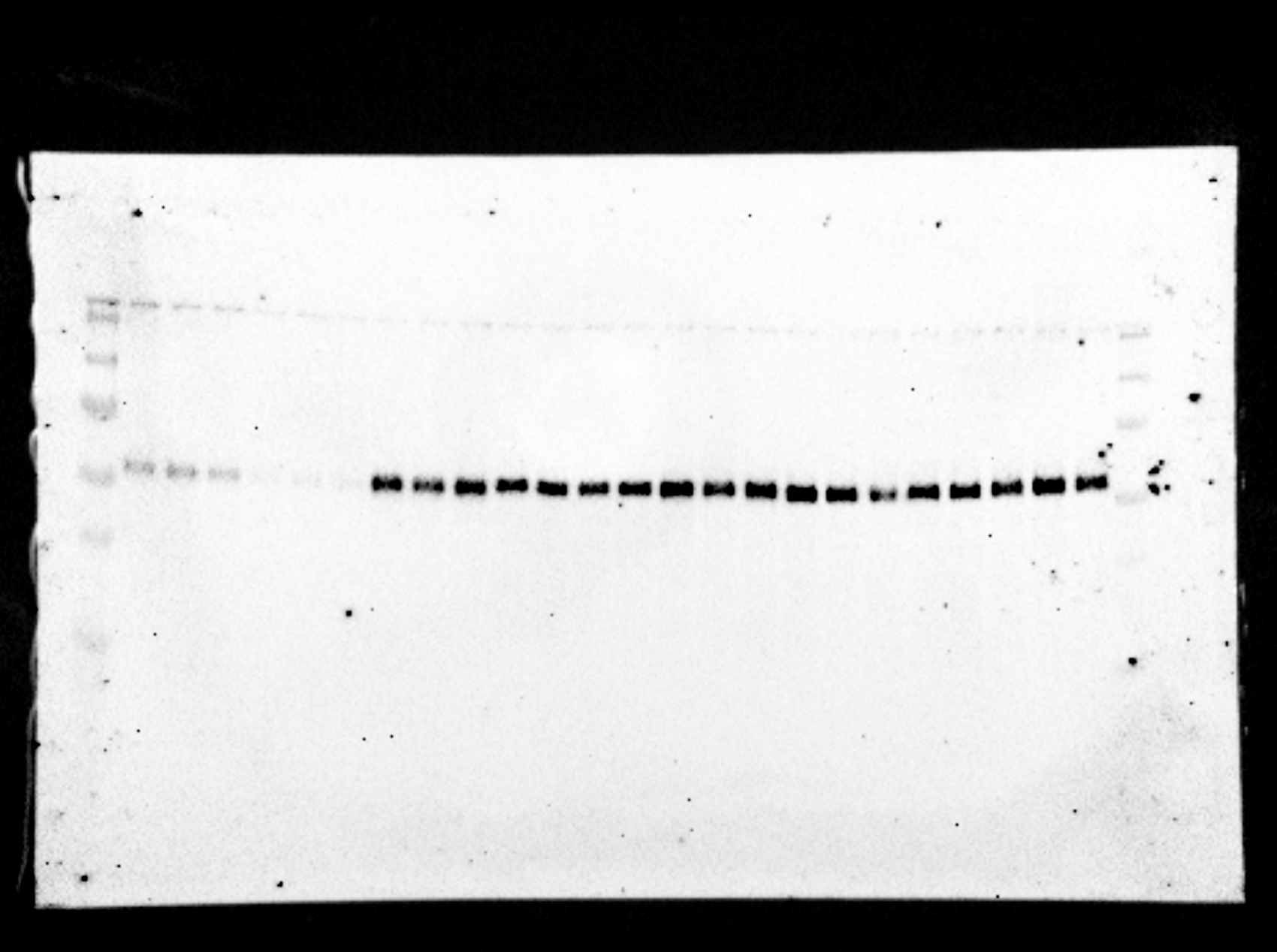

Supplement: Figure 8—figure supplement 1—source data 1. [file elife-89136-fig8-figsupp1-data1.zip › Figure 8-figure supplement 1-Source Data/Figure 8-figure supplement 1-Source Data-1 (raw WB images)/Figure 8-figure supplement 1C-pSMAD3.jpg]

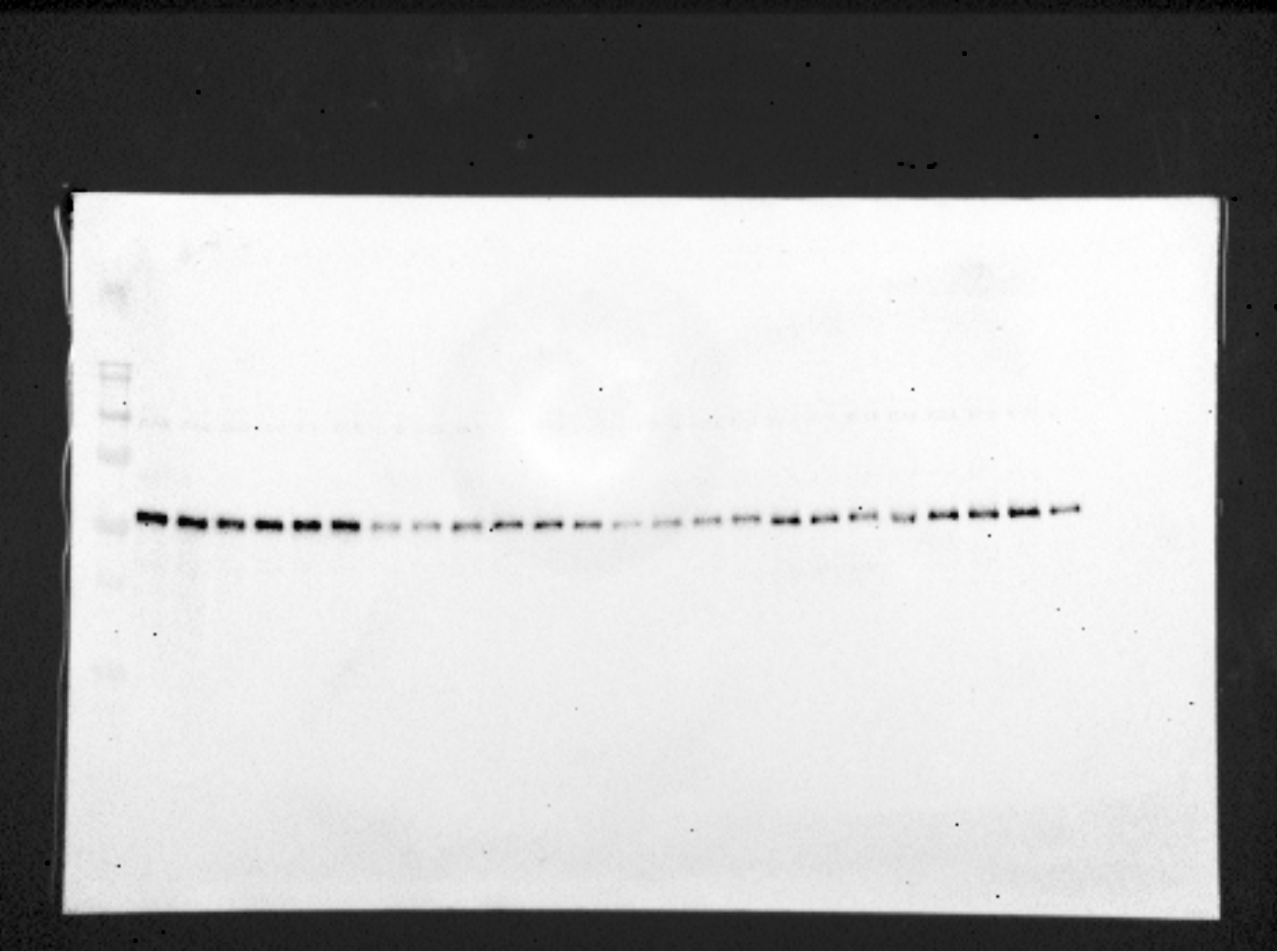

Supplement: Figure 8—figure supplement 1—source data 1. [file elife-89136-fig8-figsupp1-data1.zip › Figure 8-figure supplement 1-Source Data/Figure 8-figure supplement 1-Source Data-1 (raw WB images)/Figure 8-figure supplement 1C-SMAD3.jpg]

## Slide 1
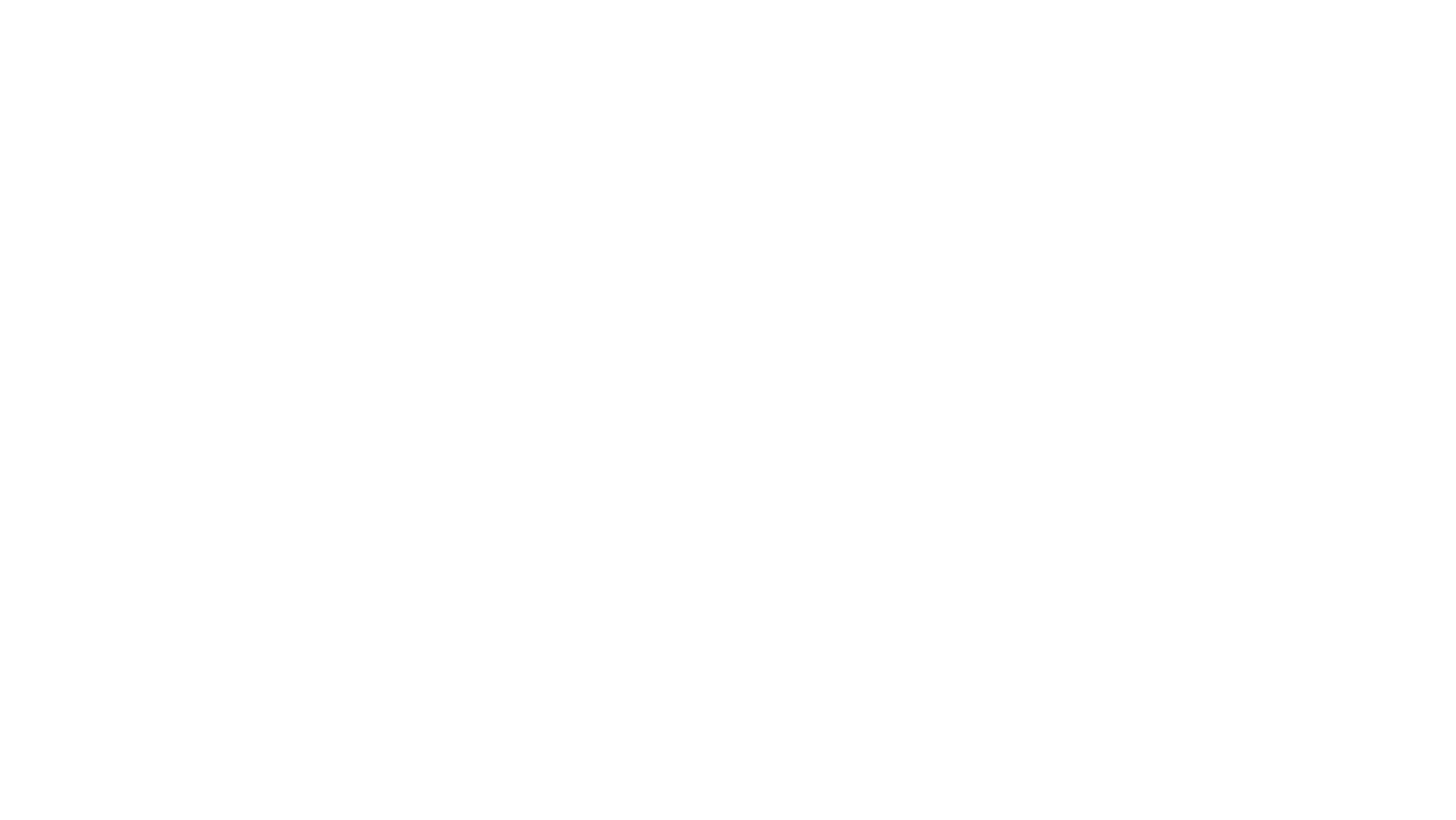

Supplement: Figure 8—figure supplement 1—source data 1. [file elife-89136-fig8-figsupp1-data1.zip › Figure 8-figure supplement 1-Source Data/Figure 8-figure supplement 1-Source Data-2 (labeled WB images)/Figure 8-figure supplement 1-Source Data 7(1).pptx]
