# Supplementary material for: Lactate transporter MCT1 in hepatic stellate cells promotes fibrotic collagen expression in nonalcoholic steatohepatitis
Source: eLife. 2024 Apr 2;12:RP89136. doi: 10.7554/eLife.89136 (PMC10987092; doi:10.7554/eLife.89136)
Supplement: Figure 2—source data 1. [file elife-89136-fig2-data1.zip › Figure 2-Source Data/Figure 2-Source Data-3 (labeled IF images).pptx]

## Slide 1
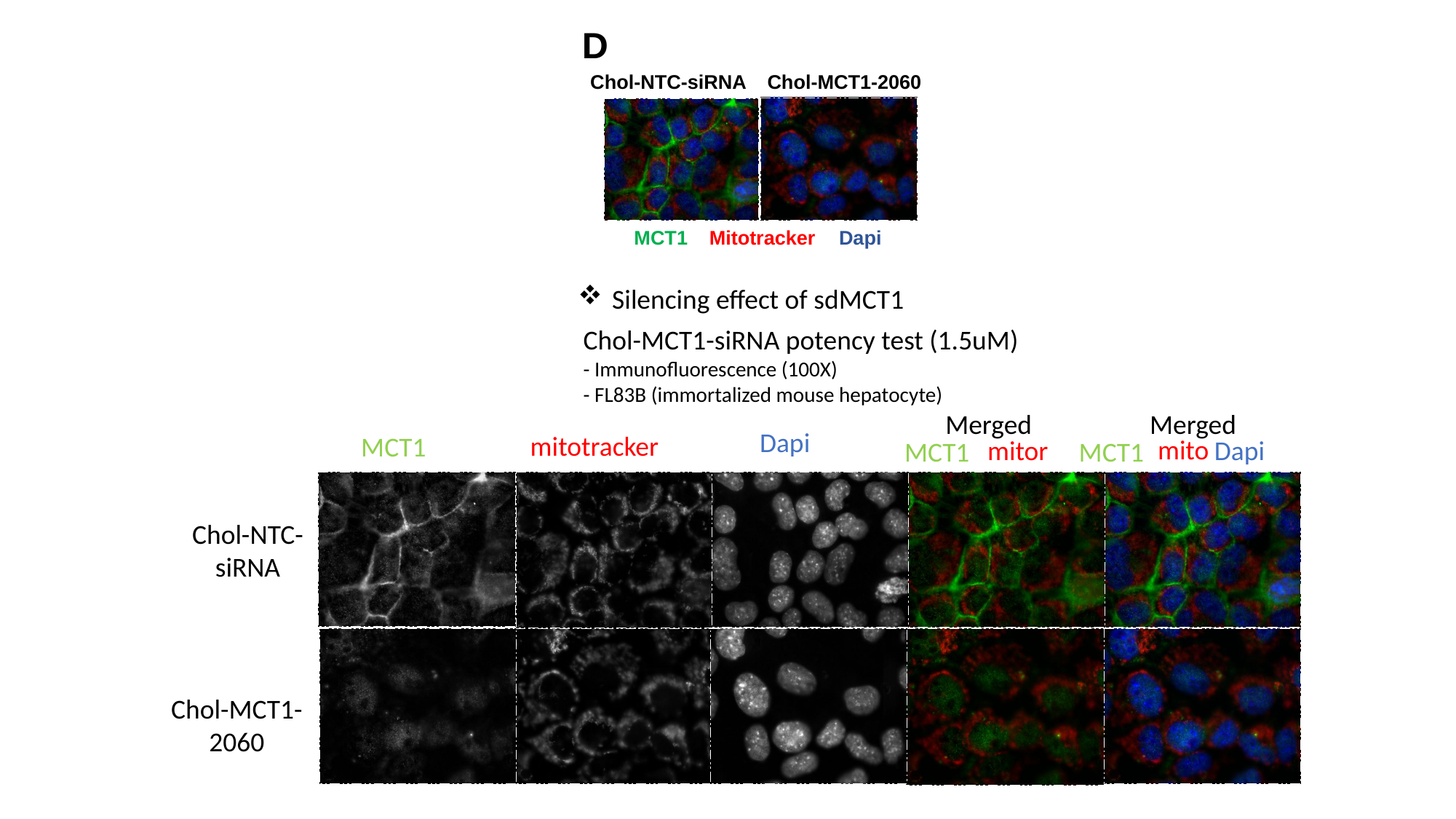

D
Chol-NTC-siRNA
Chol-MCT1-2060
MCT1
Mitotracker
Dapi
Silencing effect of sdMCT1
Chol-MCT1-siRNA potency test (1.5uM)
- Immunofluorescence (100X)
- FL83B (immortalized mouse hepatocyte)
Merged
Merged
Dapi
mitotracker
MCT1
mito
mitor
Dapi
MCT1
MCT1
Chol-NTC-siRNA
Chol-MCT1-2060
