# Supplementary material for: Lactate transporter MCT1 in hepatic stellate cells promotes fibrotic collagen expression in nonalcoholic steatohepatitis
Source: eLife. 2024 Apr 2;12:RP89136. doi: 10.7554/eLife.89136 (PMC10987092; doi:10.7554/eLife.89136)
Supplement: Figure 2—source data 1. [file elife-89136-fig2-data1.zip › Figure 2-Source Data/Figure 2-Source Data-5 (labeled WB images).pptx]

## Slide 1
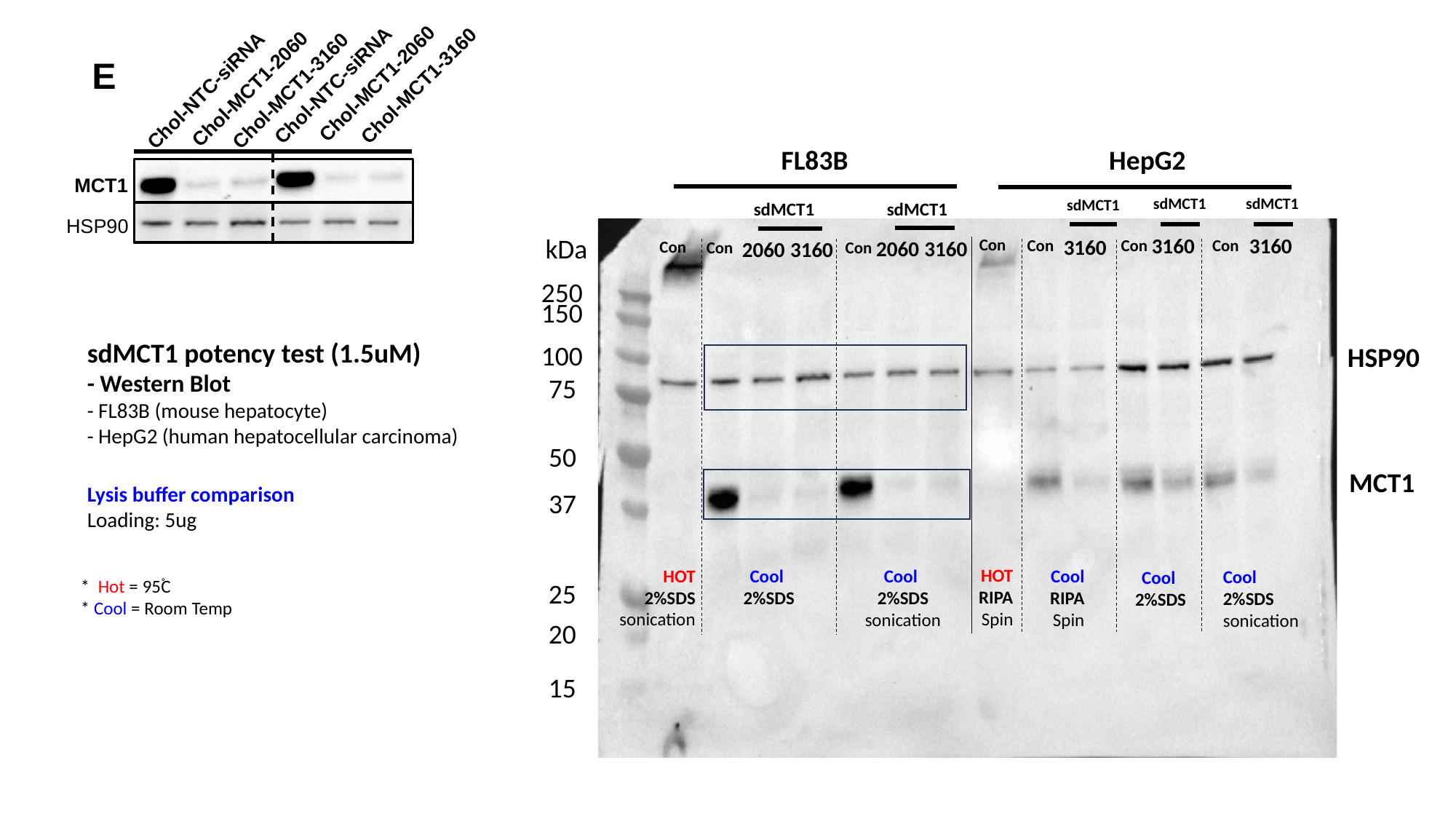

E
Chol-MCT1-2060
Chol-MCT1-3160
Chol-NTC-siRNA
Chol-MCT1-2060
Chol-MCT1-3160
Chol-NTC-siRNA
MCT1
HSP90
FL83B
HepG2
sdMCT1
sdMCT1
sdMCT1
sdMCT1
sdMCT1
Con
Con
Con
Con
2060
3160
Con
Con
Con
HOT
RIPA
Spin
HOT
2%SDS
sonication
Cool
2%SDS
Cool
2%SDS
sonication
Cool
RIPA
Spin
Cool
2%SDS
sonication
Cool
2%SDS
3160
3160
3160
2060
3160
MCT1
HSP90
* Hot = 95֯C
* Cool = Room Temp
kDa
250
150
sdMCT1 potency test (1.5uM)
- Western Blot
- FL83B (mouse hepatocyte)
- HepG2 (human hepatocellular carcinoma)
Lysis buffer comparison
Loading: 5ug
100
75
50
37
25
20
15
