# Supplementary material for: Lactate transporter MCT1 in hepatic stellate cells promotes fibrotic collagen expression in nonalcoholic steatohepatitis
Source: eLife. 2024 Apr 2;12:RP89136. doi: 10.7554/eLife.89136 (PMC10987092; doi:10.7554/eLife.89136)
Supplement: Figure 4—figure supplement 2—source data 1. [file elife-89136-fig4-figsupp2-data1.zip › Figure 4-figure supplement 2-Source Data/Figure 4-figure supplement 2-Source Data-3 (labeled WB images)/Figure 4-figure supplement 2D.pptx]

## Slide 1
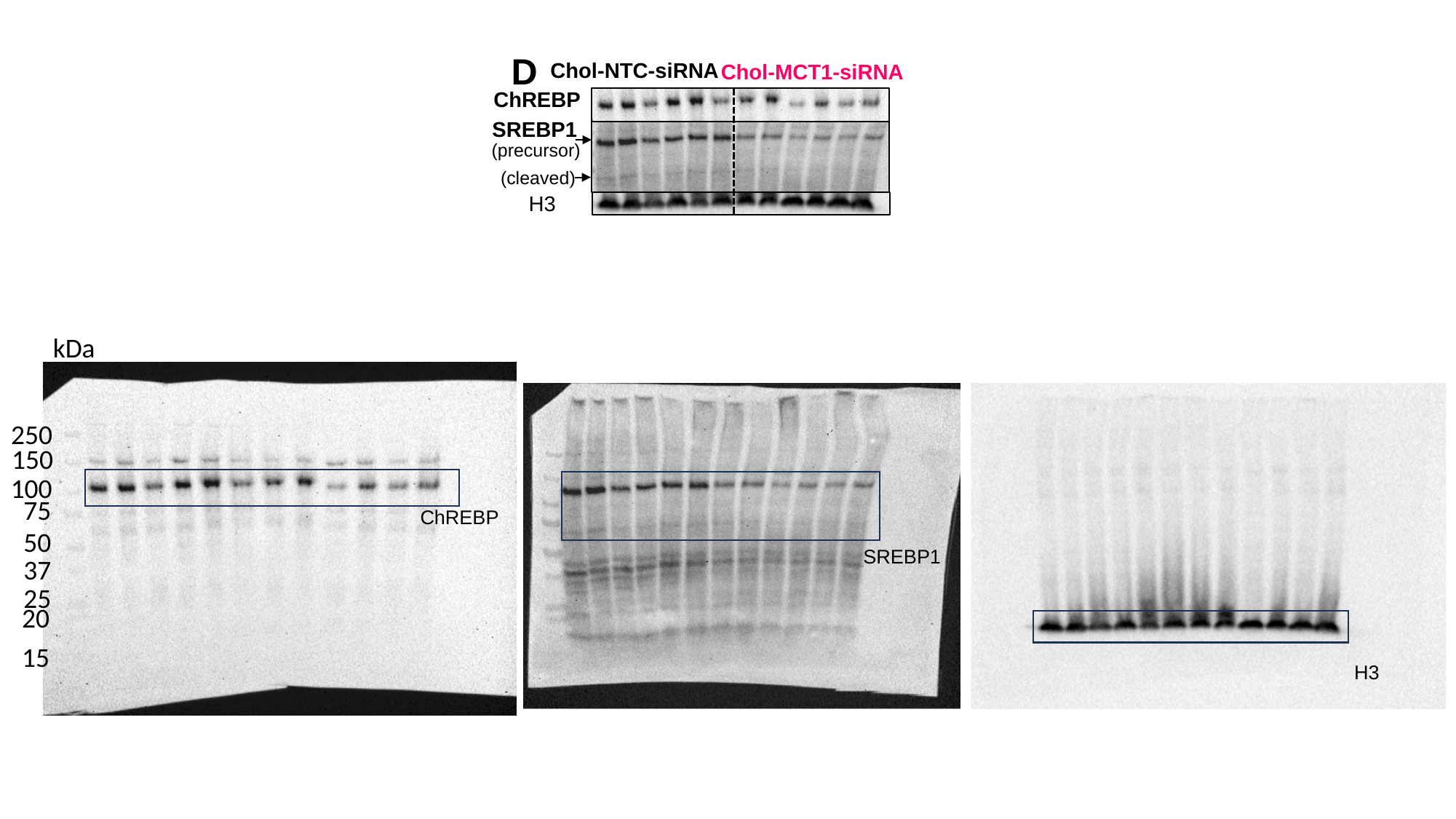

D
Chol-NTC-siRNA
Chol-MCT1-siRNA
ChREBP
SREBP1
H3
(precursor)
(cleaved)
kDa
250
150
100
75
50
37
25
20
15
SREBP1
ChREBP
H3

## Slide 2
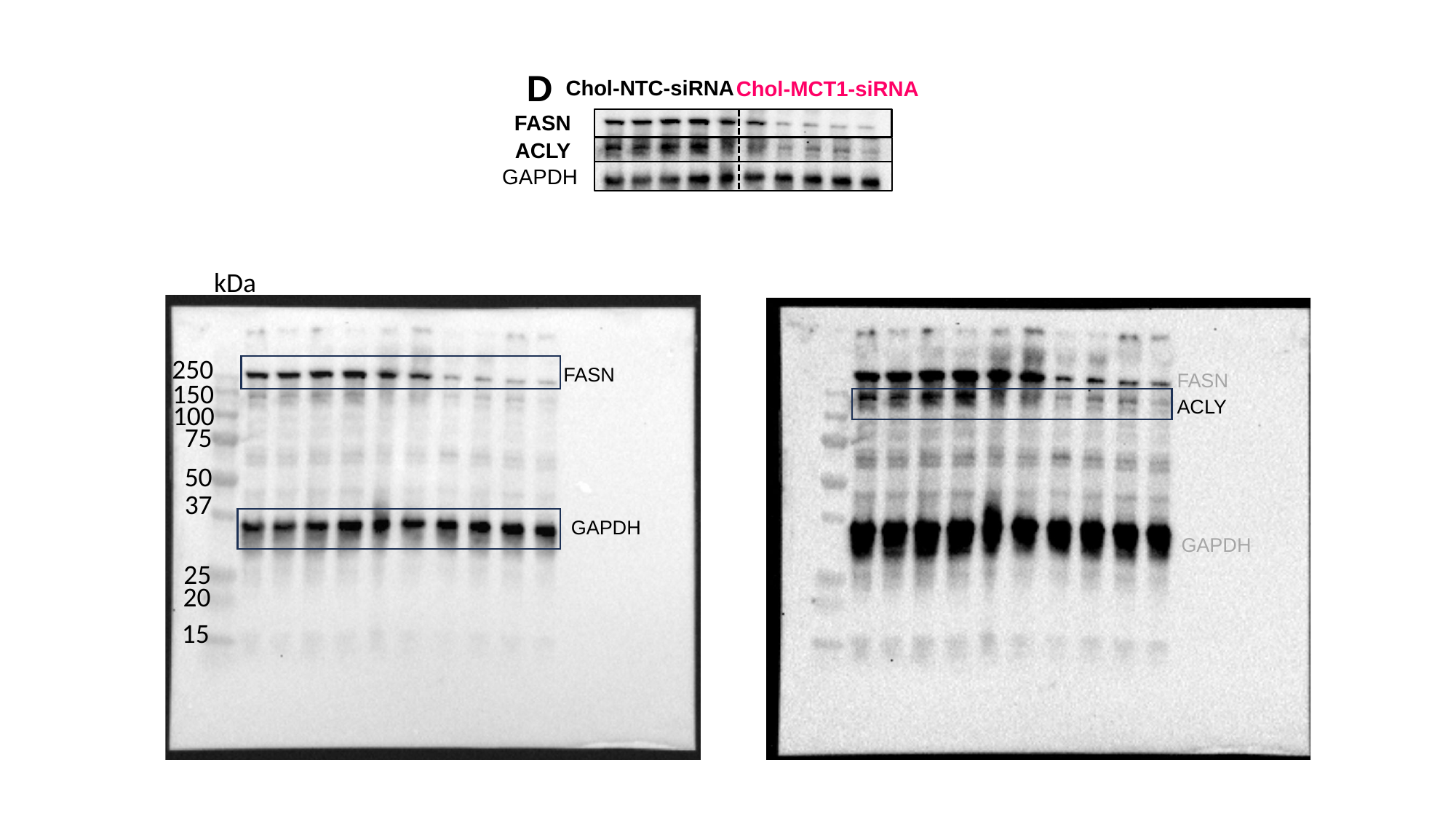

D
Chol-NTC-siRNA
Chol-MCT1-siRNA
FASN
ACLY
GAPDH
kDa
250
150
100
75
50
37
25
20
15
FASN
FASN
ACLY
GAPDH
GAPDH

## Slide 3
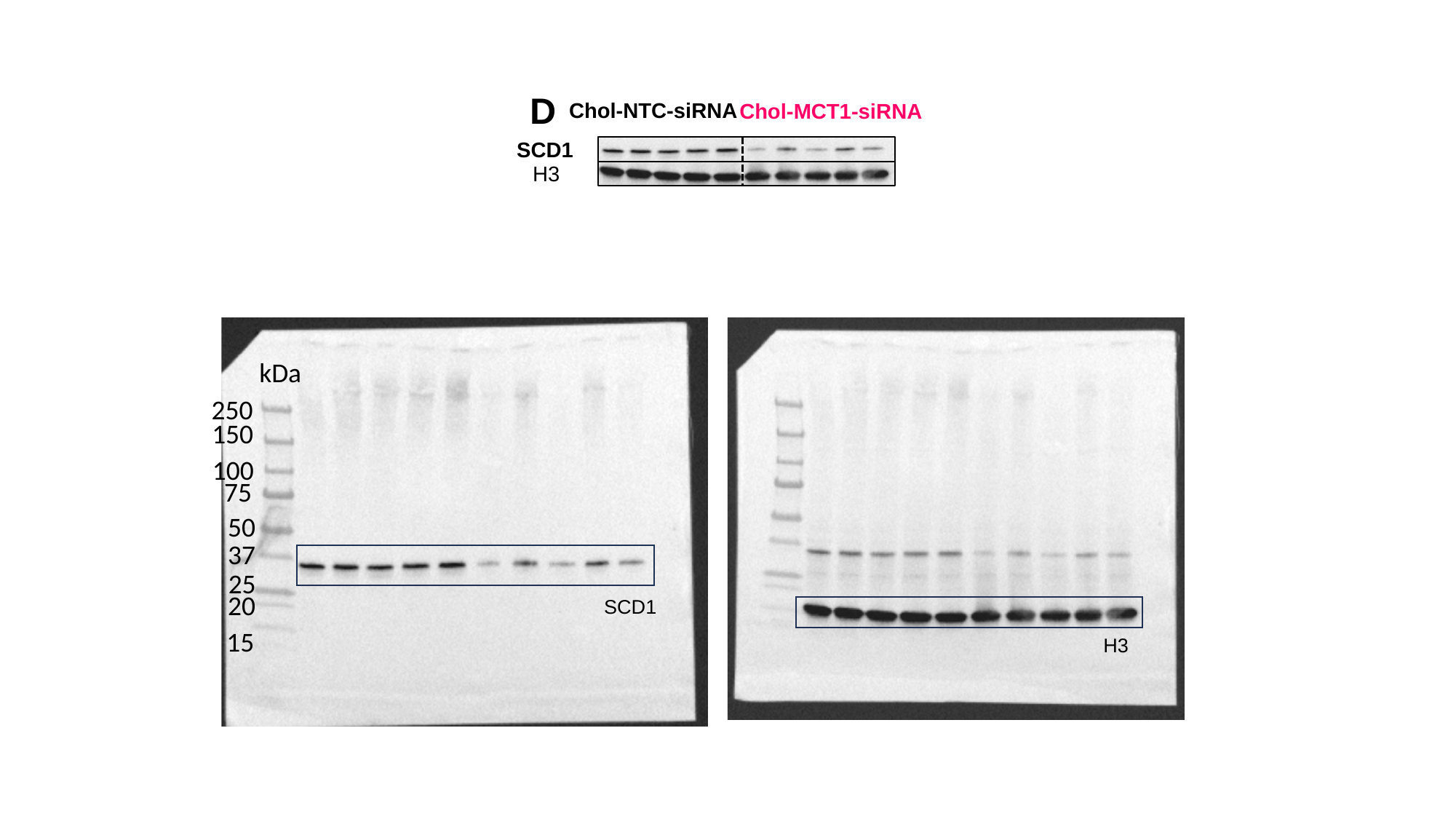

D
Chol-NTC-siRNA
Chol-MCT1-siRNA
SCD1
H3
kDa
250
150
100
75
50
37
25
20
15
SCD1
H3
