# Supplementary material for: Lactate transporter MCT1 in hepatic stellate cells promotes fibrotic collagen expression in nonalcoholic steatohepatitis
Source: eLife. 2024 Apr 2;12:RP89136. doi: 10.7554/eLife.89136 (PMC10987092; doi:10.7554/eLife.89136)
Supplement: Figure 5—source data 1. [file elife-89136-fig5-data1.zip › Figure 5-Source Data/Figure 5-Source Data-2 (labeled WB images).pptx]

## Slide 1
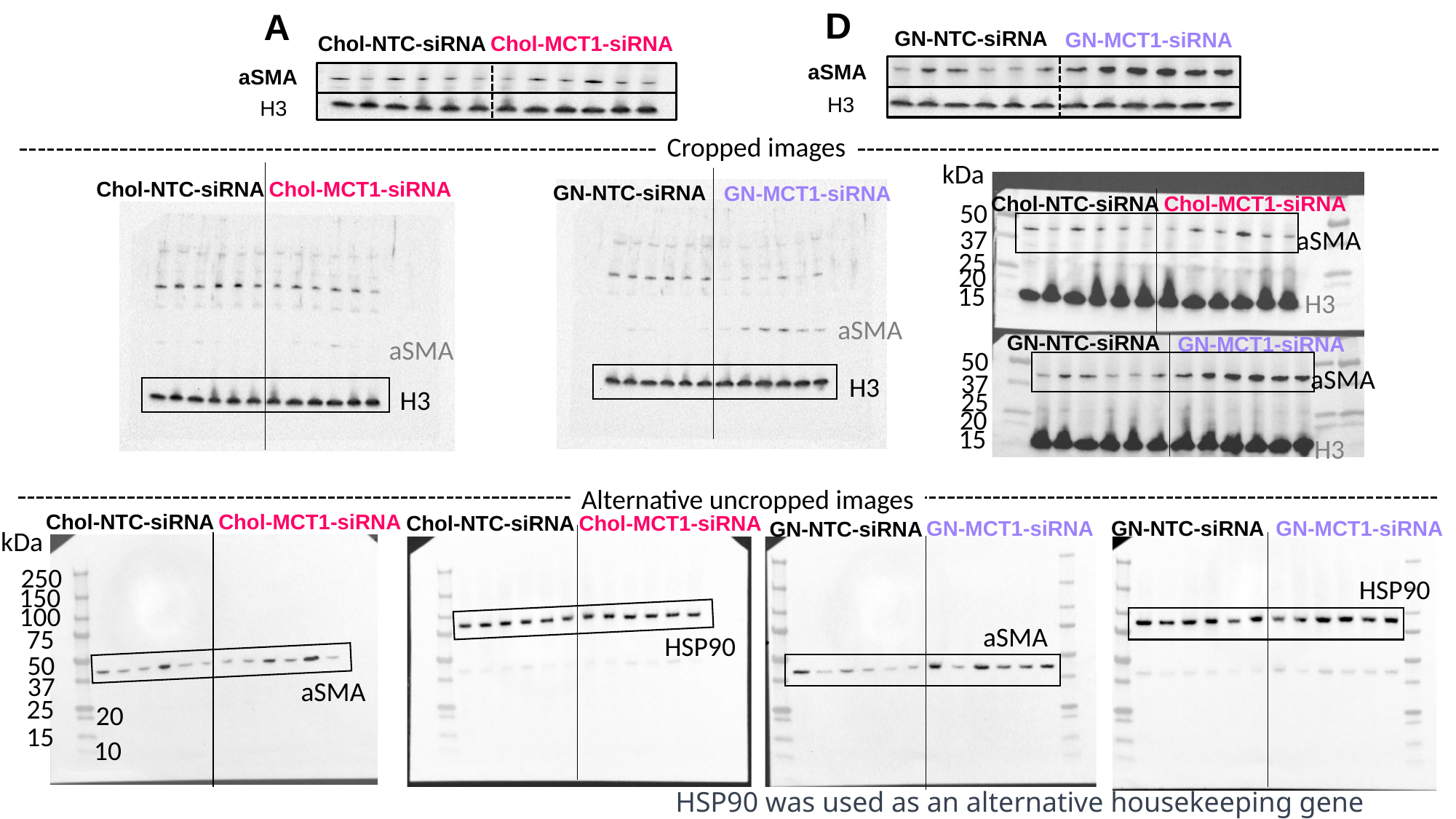

D
GN-NTC-siRNA
GN-MCT1-siRNA
aSMA
H3
A
Chol-NTC-siRNA
Chol-MCT1-siRNA
aSMA
H3
Cropped images
kDa
aSMA
H3
aSMA
H3
H3
Chol-NTC-siRNA
Chol-MCT1-siRNA
GN-NTC-siRNA
GN-MCT1-siRNA
Chol-NTC-siRNA
Chol-MCT1-siRNA
50
37
aSMA
25
20
15
H3
GN-NTC-siRNA
GN-MCT1-siRNA
50
aSMA
37
25
20
15
H3
Alternative uncropped images
Chol-NTC-siRNA
Chol-MCT1-siRNA
Chol-NTC-siRNA
Chol-MCT1-siRNA
GN-MCT1-siRNA
GN-MCT1-siRNA
GN-NTC-siRNA
GN-NTC-siRNA
kDa
250
HSP90
150
100
aSMA
75
HSP90
50
37
aSMA
25
20
15
10
HSP90 was used as an alternative housekeeping gene instead of H3

## Slide 2
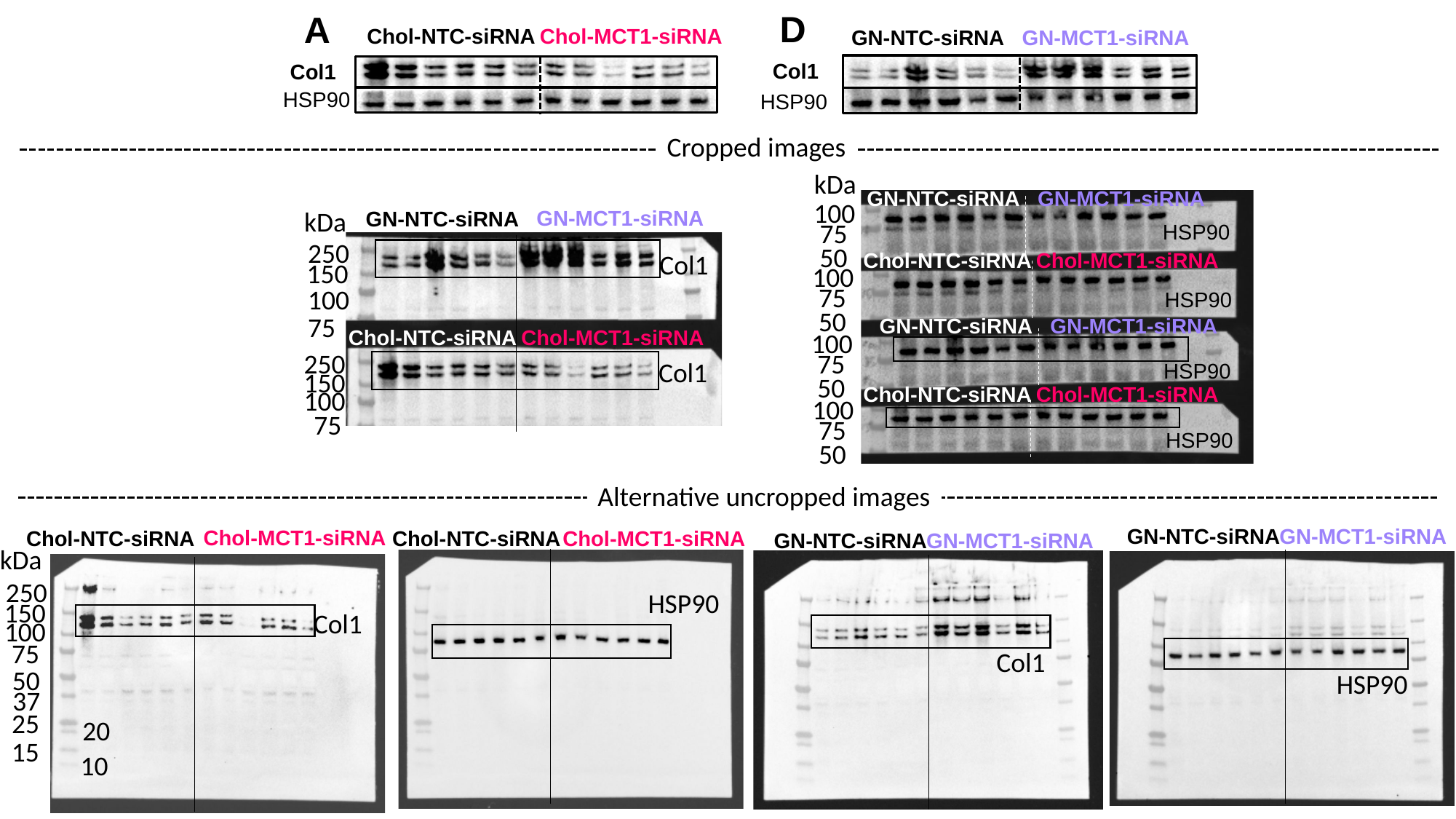

D
GN-MCT1-siRNA
GN-NTC-siRNA
HSP90
Col1
A
Chol-NTC-siRNA
Chol-MCT1-siRNA
Col1
HSP90
Cropped images
kDa
GN-MCT1-siRNA
GN-NTC-siRNA
100
75
HSP90
50
Chol-NTC-siRNA
Chol-MCT1-siRNA
100
75
HSP90
50
GN-MCT1-siRNA
GN-NTC-siRNA
100
75
HSP90
50
Chol-NTC-siRNA
Chol-MCT1-siRNA
100
75
HSP90
50
kDa
250
Col1
150
100
75
250
Col1
150
100
75
GN-MCT1-siRNA
GN-NTC-siRNA
Chol-NTC-siRNA
Chol-MCT1-siRNA
Alternative uncropped images
GN-MCT1-siRNA
GN-NTC-siRNA
HSP90
Chol-MCT1-siRNA
Chol-NTC-siRNA
Chol-NTC-siRNA
Chol-MCT1-siRNA
HSP90
GN-MCT1-siRNA
GN-NTC-siRNA
Col1
kDa
250
150
Col1
100
75
50
37
25
20
15
10
