# Supplementary material for: Lactate transporter MCT1 in hepatic stellate cells promotes fibrotic collagen expression in nonalcoholic steatohepatitis
Source: eLife. 2024 Apr 2;12:RP89136. doi: 10.7554/eLife.89136 (PMC10987092; doi:10.7554/eLife.89136)
Supplement: Figure 8—source data 1. [file elife-89136-fig8-data1.zip › Figure 8-Source Data/Figure 8-Source Data-3 (labeled WB images)/Figuer 8F.pptx]

## Slide 1
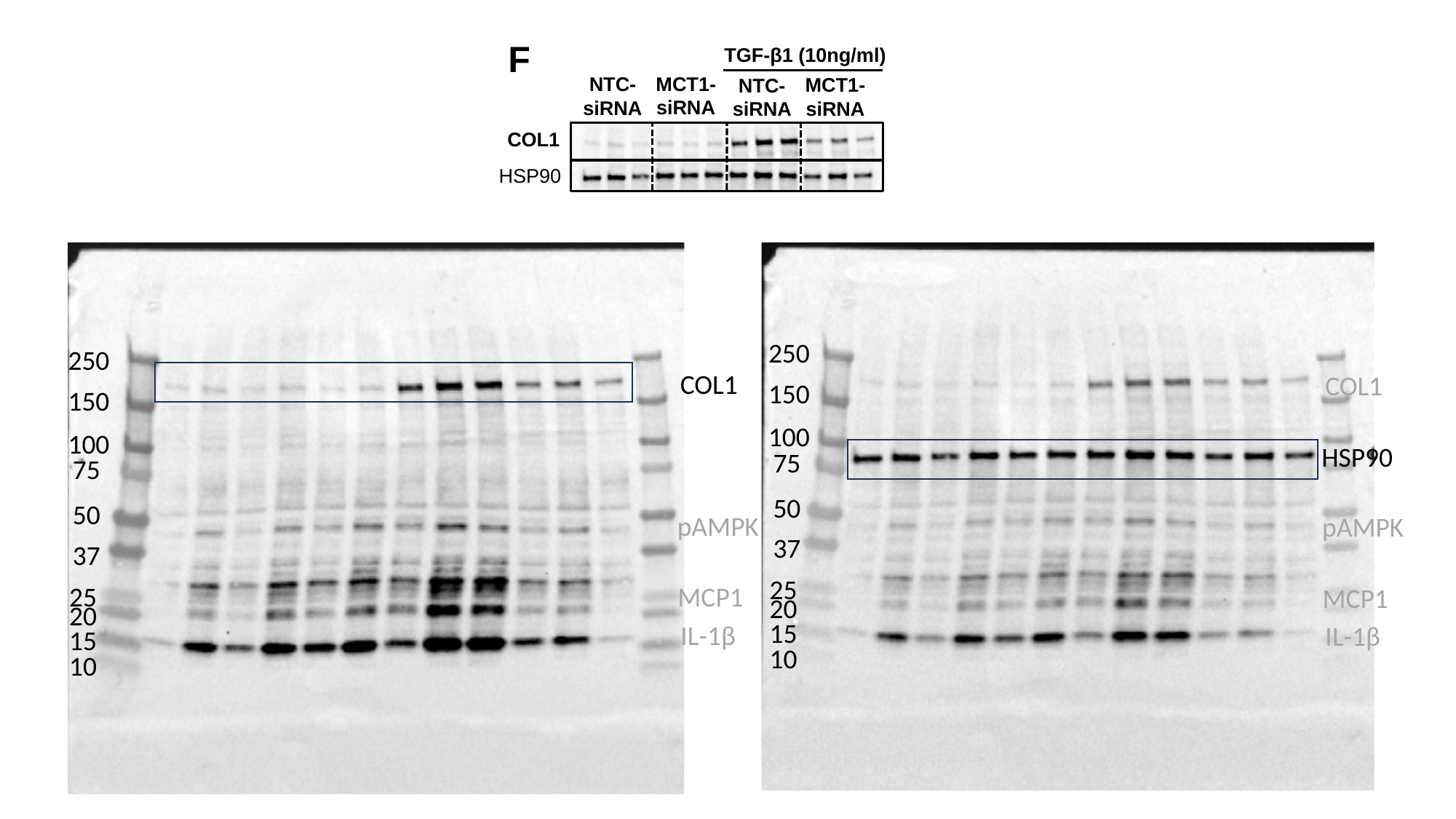

F
TGF-β1 (10ng/ml)
MCT1-
siRNA
NTC-
siRNA
COL1
HSP90
MCT1-
siRNA
NTC-
siRNA
250
250
COL1
COL1
150
150
100
100
HSP90
75
75
50
50
pAMPK
pAMPK
37
37
25
25
MCP1
MCP1
20
20
15
IL-1β
IL-1β
15
10
10
