# Supplementary material for: Lactate transporter MCT1 in hepatic stellate cells promotes fibrotic collagen expression in nonalcoholic steatohepatitis
Source: eLife. 2024 Apr 2;12:RP89136. doi: 10.7554/eLife.89136 (PMC10987092; doi:10.7554/eLife.89136)
Supplement: Figure 8—figure supplement 1—source data 1. [file elife-89136-fig8-figsupp1-data1.zip › Figure 8-figure supplement 1-Source Data/Figure 8-figure supplement 1-Source Data-2 (labeled WB images)/Figure 8-figure supplement 1-Source Data 1C.pptx]

## Slide 1
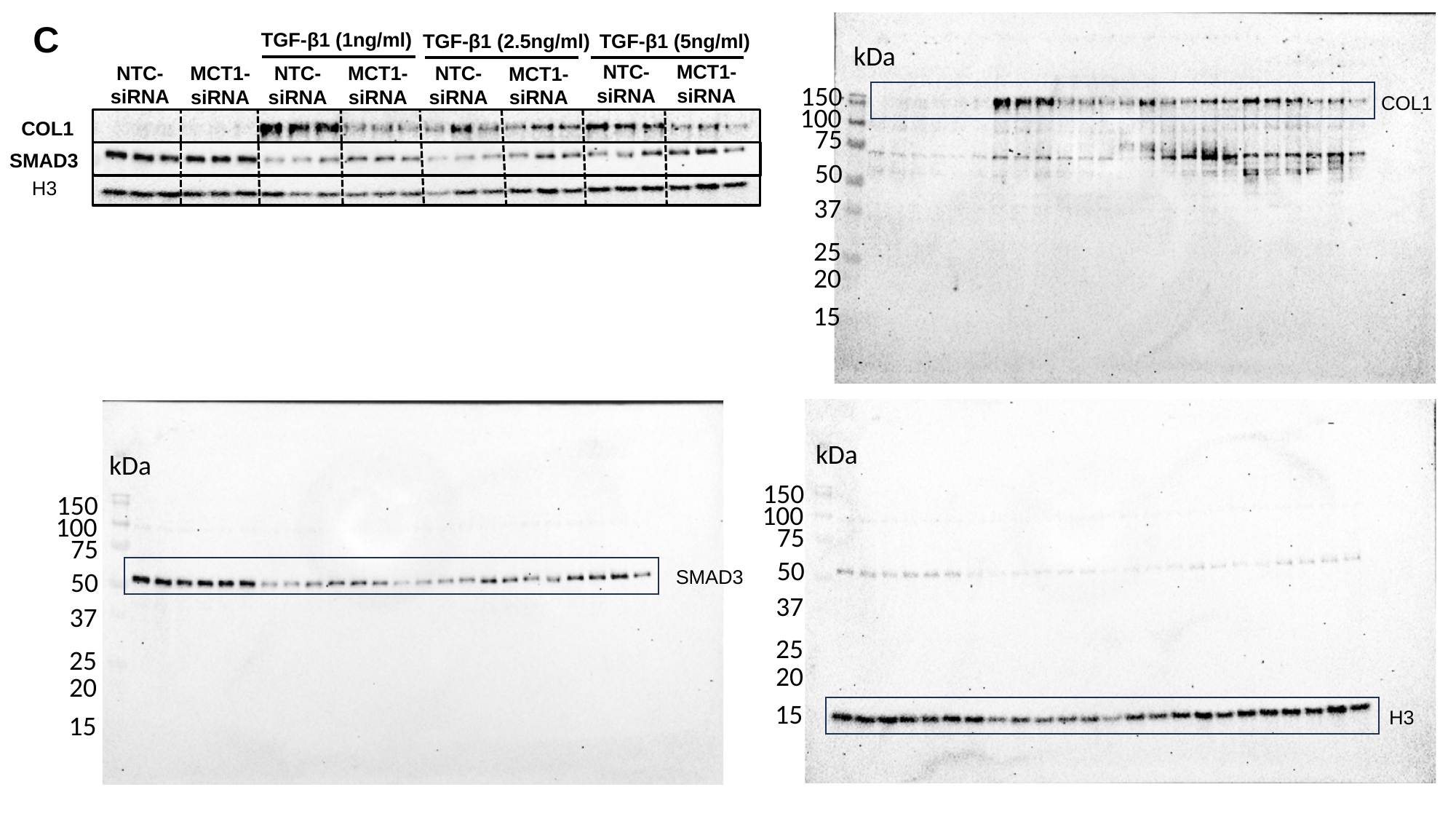

C
TGF-β1 (1ng/ml)
TGF-β1 (2.5ng/ml)
TGF-β1 (5ng/ml)
NTC-siRNA
MCT1-siRNA
NTC-siRNA
NTC-siRNA
MCT1-siRNA
NTC-siRNA
MCT1-siRNA
MCT1-siRNA
COL1
SMAD3
H3
kDa
150
COL1
100
75
50
37
25
20
15
kDa
kDa
150
150
100
100
75
75
50
SMAD3
50
37
37
25
25
20
20
15
H3
15

## Slide 2
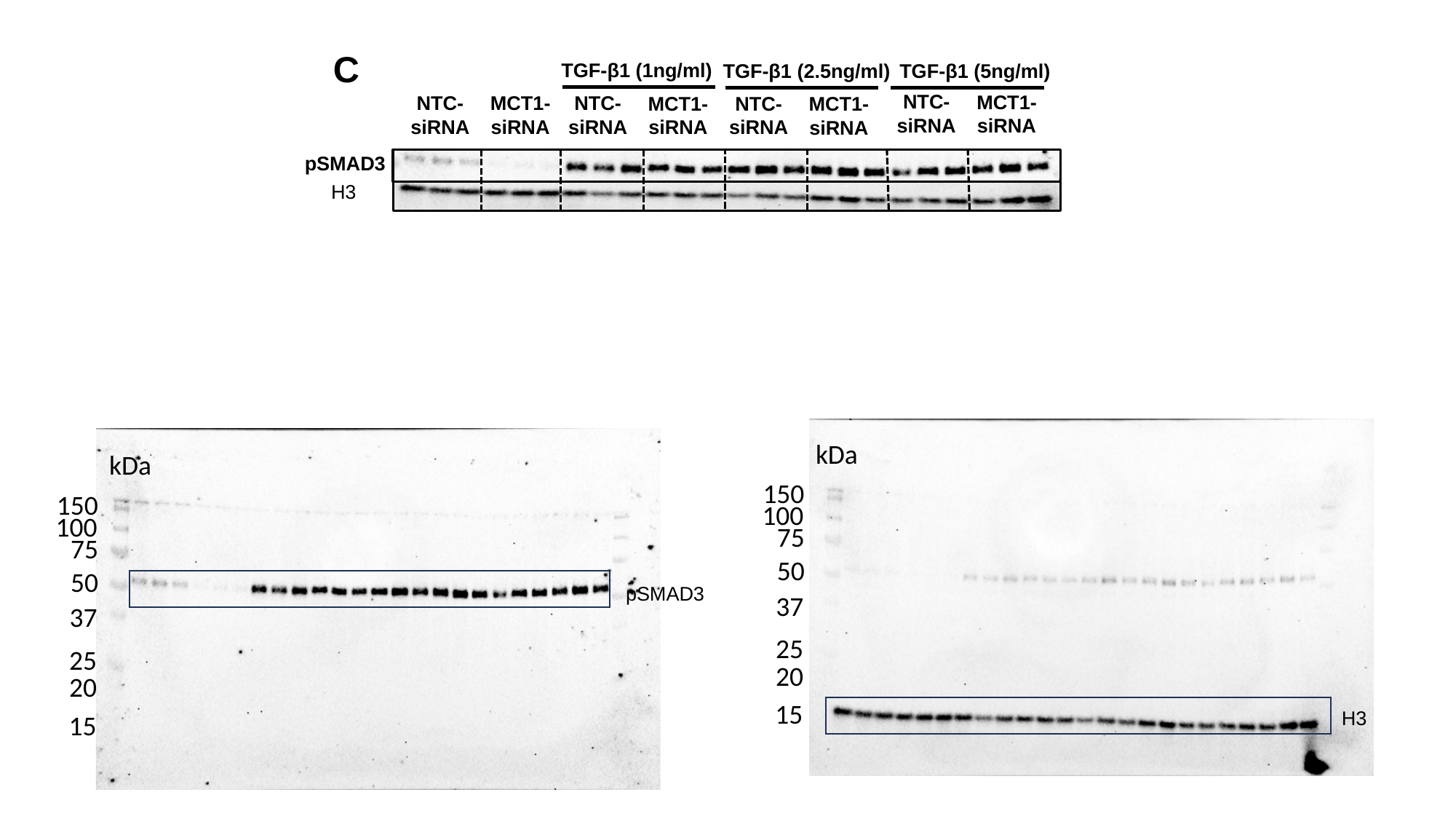

C
TGF-β1 (1ng/ml)
TGF-β1 (2.5ng/ml)
TGF-β1 (5ng/ml)
NTC-siRNA
MCT1-siRNA
NTC-siRNA
NTC-siRNA
MCT1-siRNA
NTC-siRNA
MCT1-siRNA
MCT1-siRNA
pSMAD3
H3
kDa
kDa
150
150
100
100
75
75
50
50
pSMAD3
37
37
25
25
20
20
15
H3
15
